# Supplementary material for: Common dolphin whistle responses to experimental mid-frequency sonar
Source: PLoS One. 2024 Apr 26;19(4):e0302035. doi: 10.1371/journal.pone.0302035 (PMC11051594; doi:10.1371/journal.pone.0302035)
Supplement: S1 Fig — Detection settings for the PAMGuard Whistle and Moan Detector. (PDF) [file pone.0302035.s001.pdf]

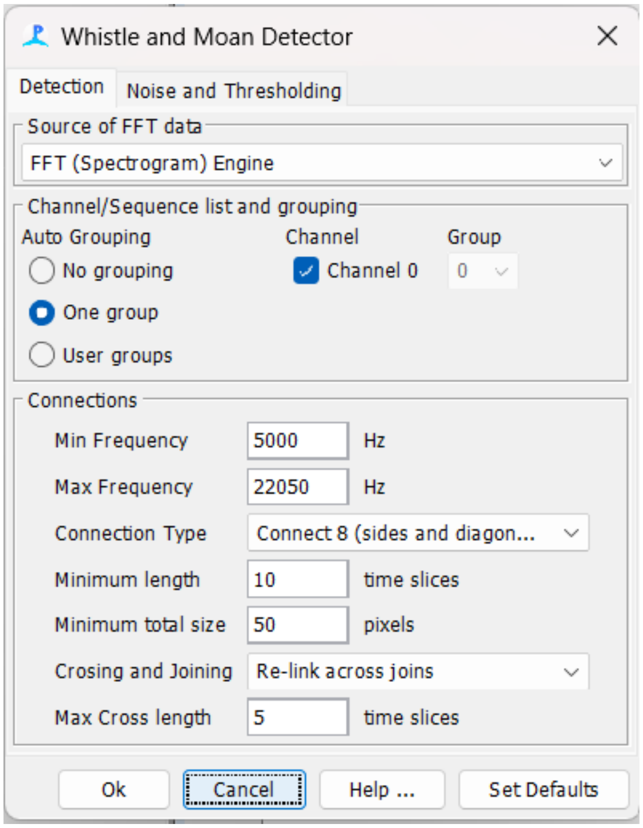
Whistle and Moan Detector
Detection
Noise and Thresholding
Source of FFT data
FFT (Spectrogram) Engine
Channel/Sequence list and grouping
Auto Grouping
Channel
Group
No grouping
Channel 0
0
One group
User groups
Connections
Min Frequency
5000
Hz
Max Frequency
22050
Hz
Connection Type
Connect 8 (sides and diagon...
Minimum length
10
time slices
Minimum total size
50
pixels
Crosing and Joining
Re-link across joins
Max Cross length
5
time slices
Ok
Cancel
Help ...
Set Defaults
