# Supplementary material for: Common dolphin whistle responses to experimental mid-frequency sonar
Source: PLoS One. 2024 Apr 26;19(4):e0302035. doi: 10.1371/journal.pone.0302035 (PMC11051594; doi:10.1371/journal.pone.0302035)
Supplement: S2 Fig — CEE-ID (year and number) is given in the bottom right of each page. The red dashed line indicates the start of the exposure period, with dashed grey lines indicating the timing of each ping (in an MFAS exposure) or control treatment. The solid gray vertical line indicates the start of the post-exposure period. (PDF) [file pone.0302035.s002.pdf]

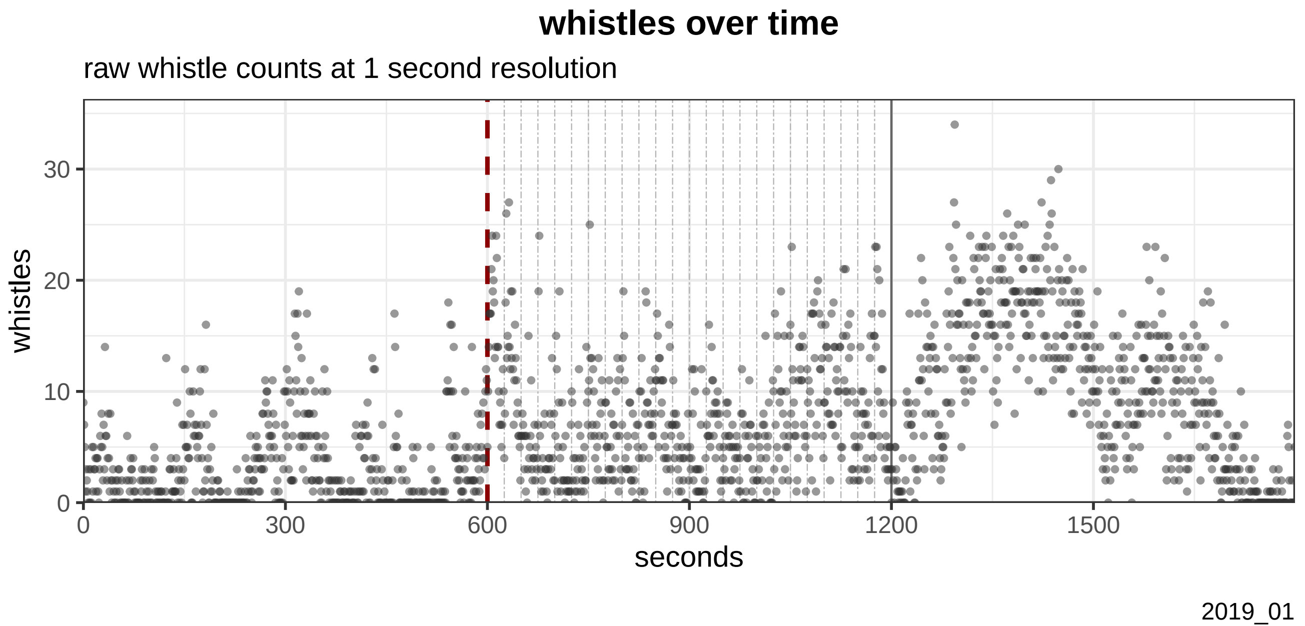

whistles over time
raw whistle counts at 1 second resolution
whistles
30
20
10
0
0
300
600
900
1200
1500
seconds
2019_01

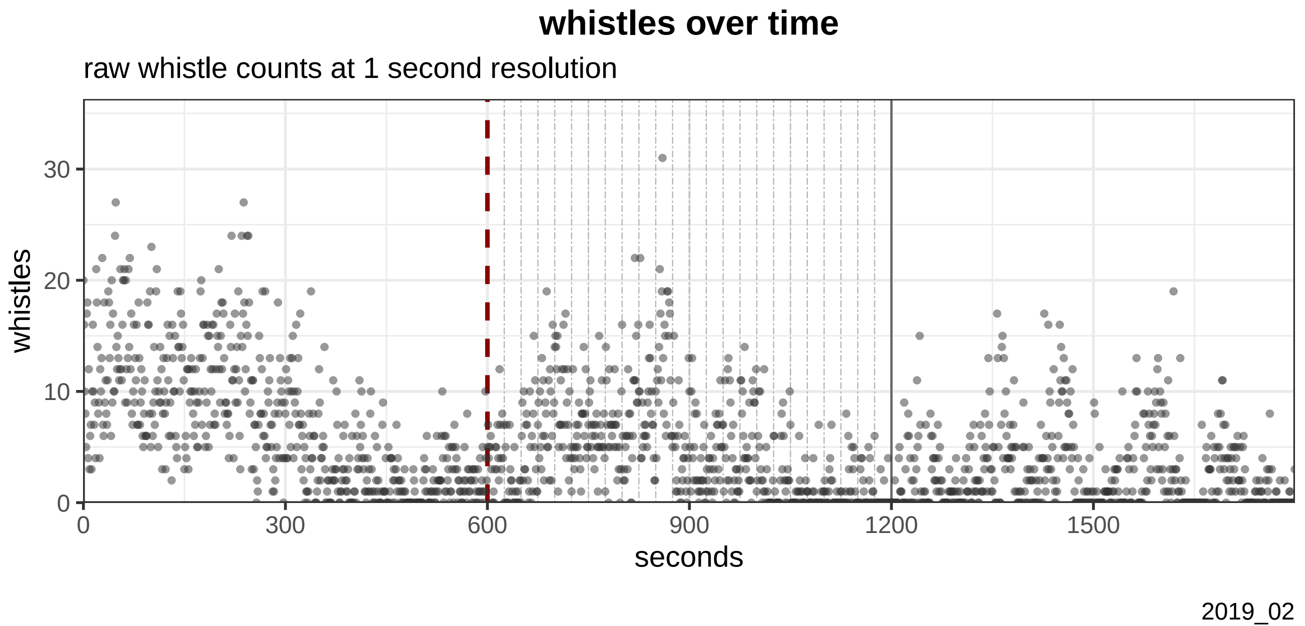

whistles over time
raw whistle counts at 1 second resolution
whistles
30
20
10
0
0
300
600
900
1200
1500
seconds
2019_02

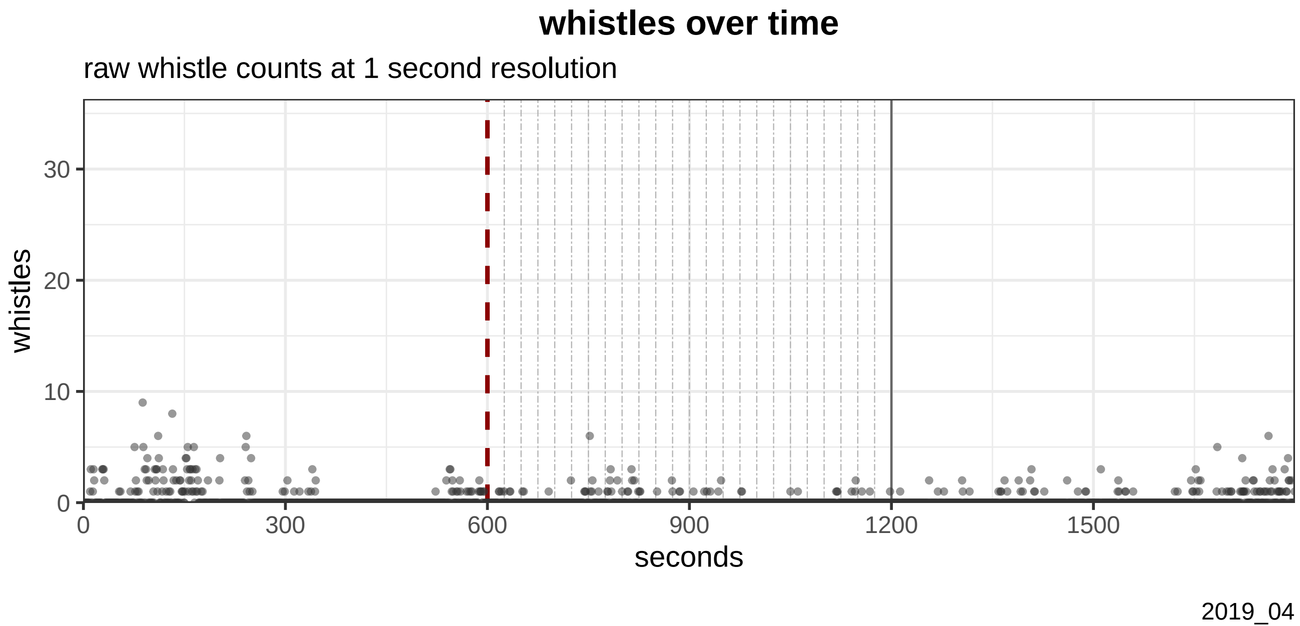
whistles over time
raw whistle counts at 1 second resolution
whistles
30
20
10
0
0
300
600
900
1200
1500
seconds
2019_04

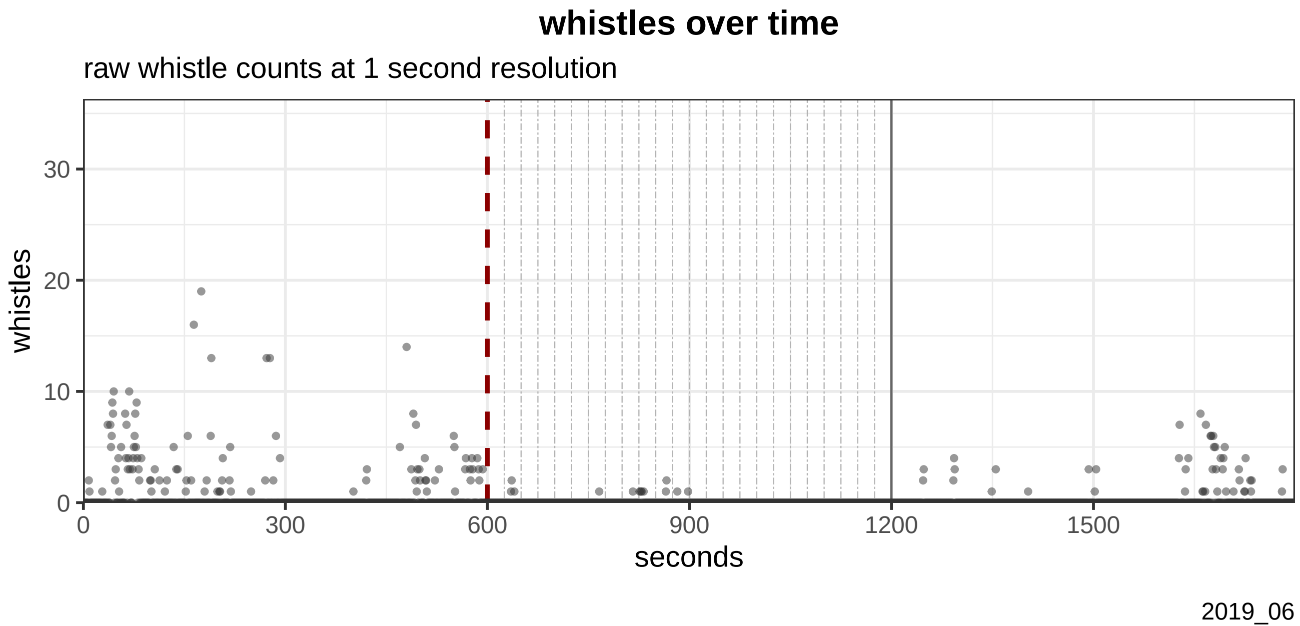
whistles over time
raw whistle counts at 1 second resolution
whistles
30
20
10
0
0
300
600
900
1200
1500
seconds
2019_06

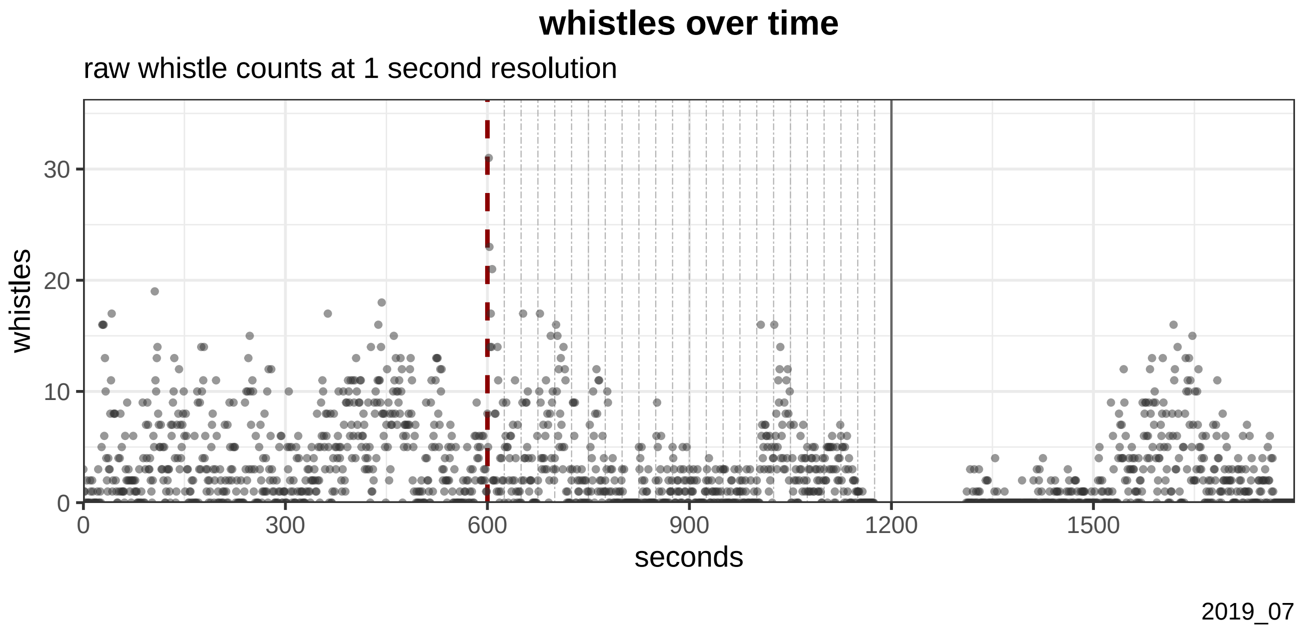

whistles over time
raw whistle counts at 1 second resolution
whistles
30
20
10
0
0
300
600
900
1200
1500
seconds
2019_07

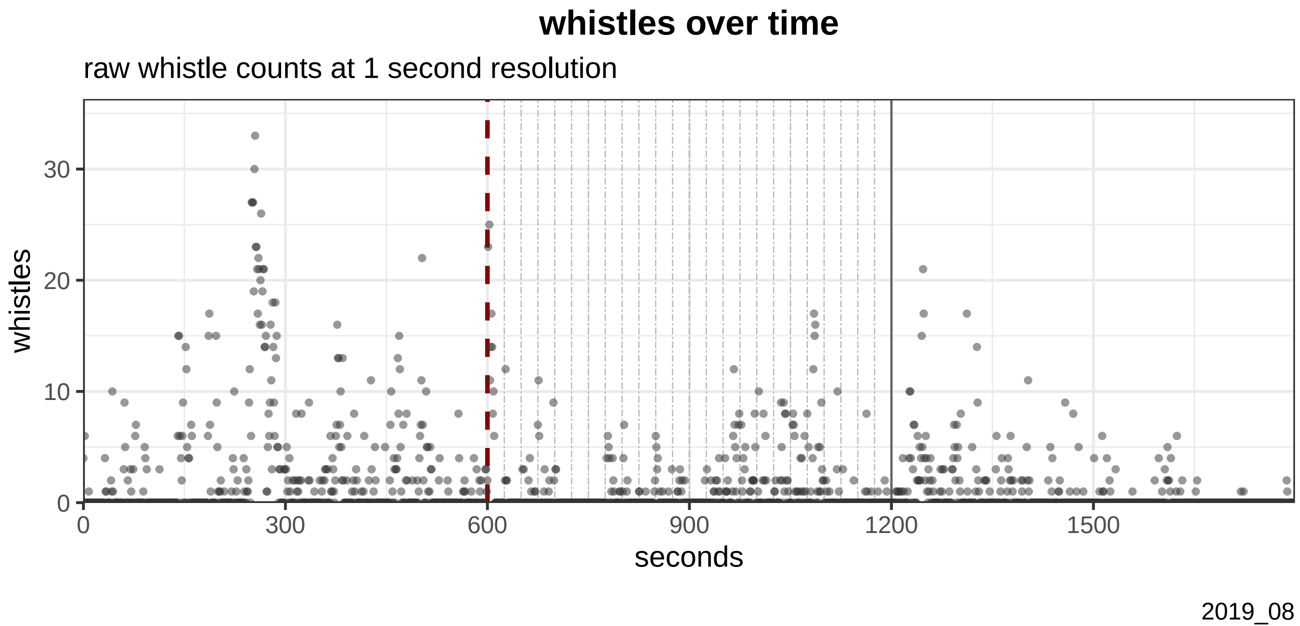

whistles over time
raw whistle counts at 1 second resolution
whistles
30
20
10
0
0
300
600
900
1200
1500
seconds
2019_08

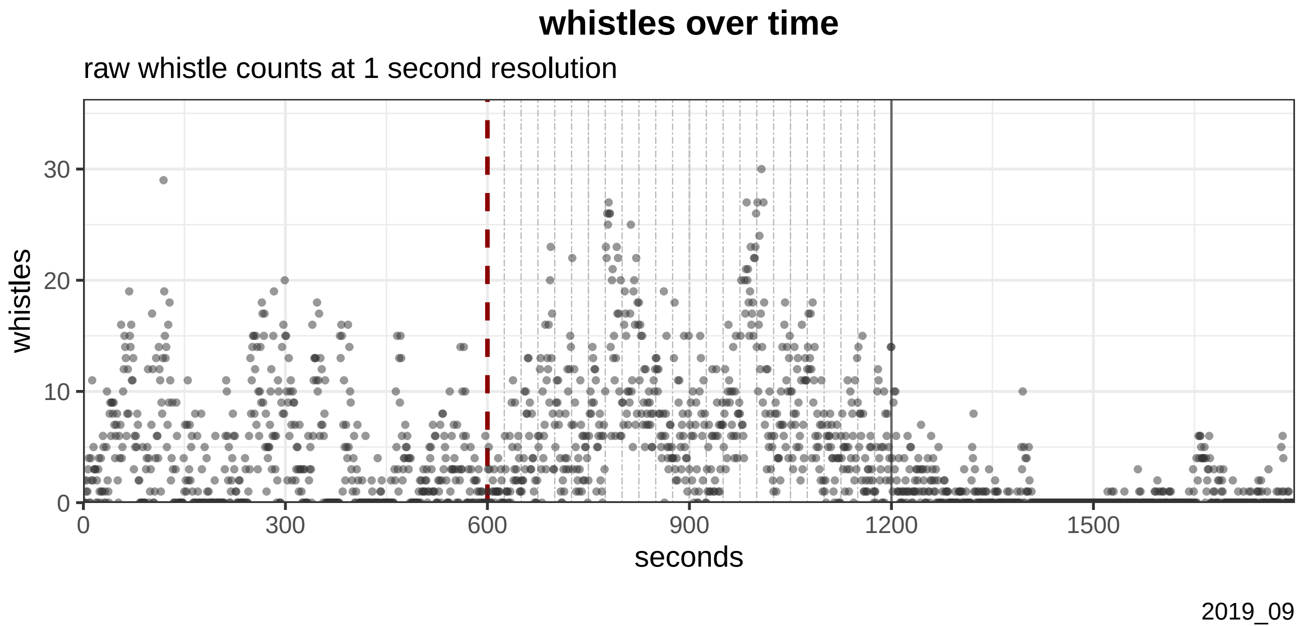
whistles over time
raw whistle counts at 1 second resolution
whistles
30
20
10
0
0
300
600
900
1200
1500
seconds
2019_09

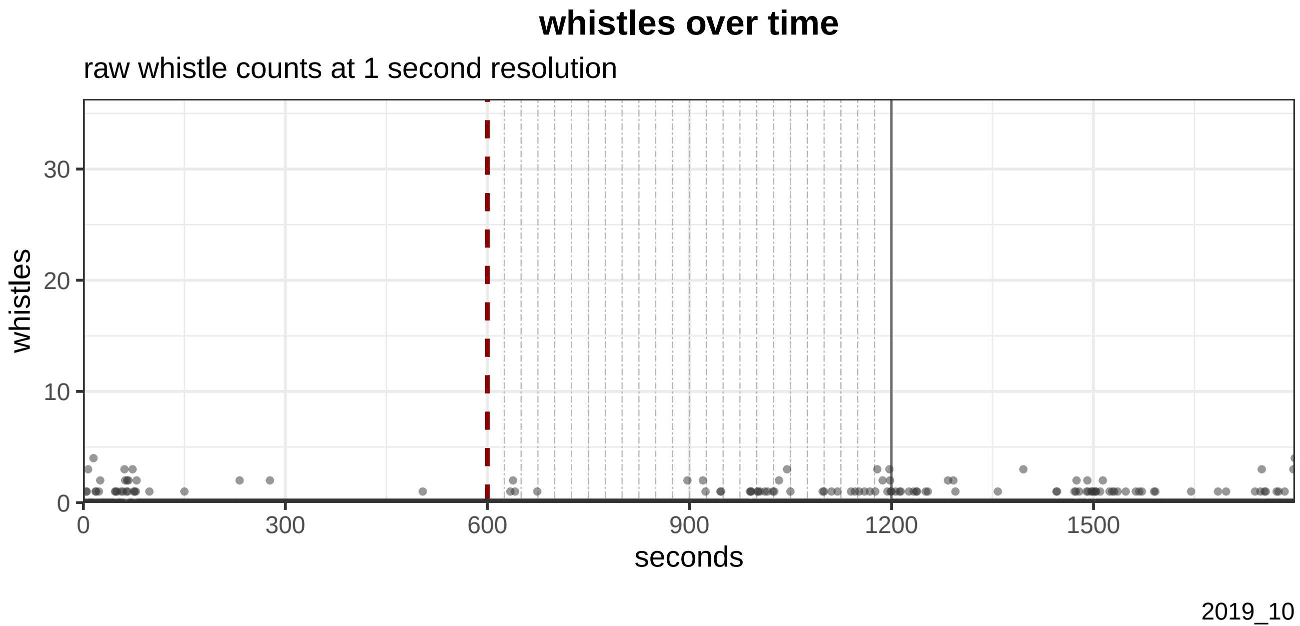
whistles over time
raw whistle counts at 1 second resolution
whistles
30
20
10
0
0
300
600
900
1200
1500
seconds
2019_10

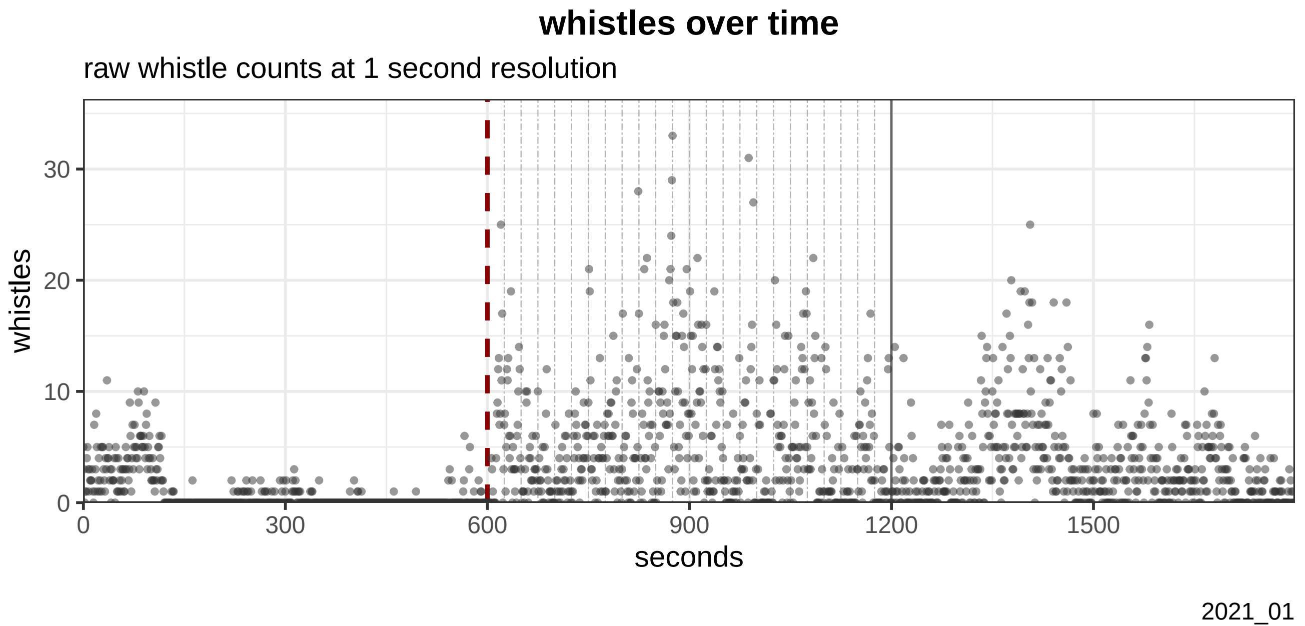
whistles over time
raw whistle counts at 1 second resolution
whistles
seconds
0
10
20
30
0
300
600
900
1200
1500
2021_01

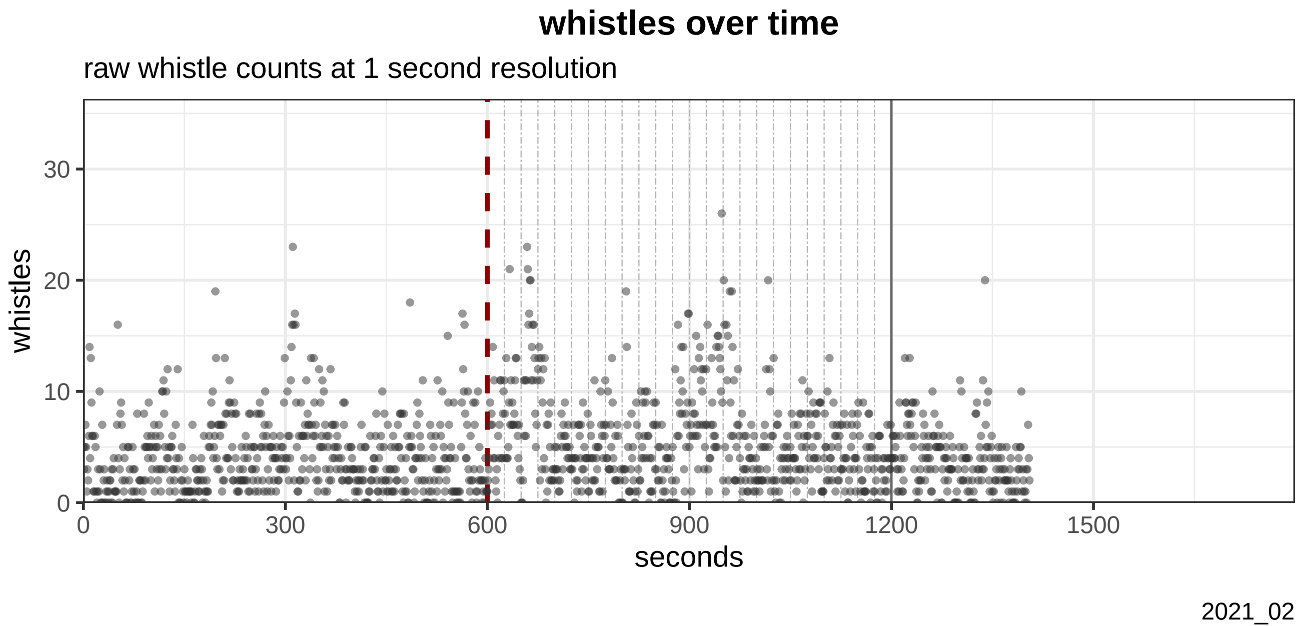

whistles over time
raw whistle counts at 1 second resolution
whistles
seconds
0
10
20
30
0
300
600
900
1200
1500
2021_02

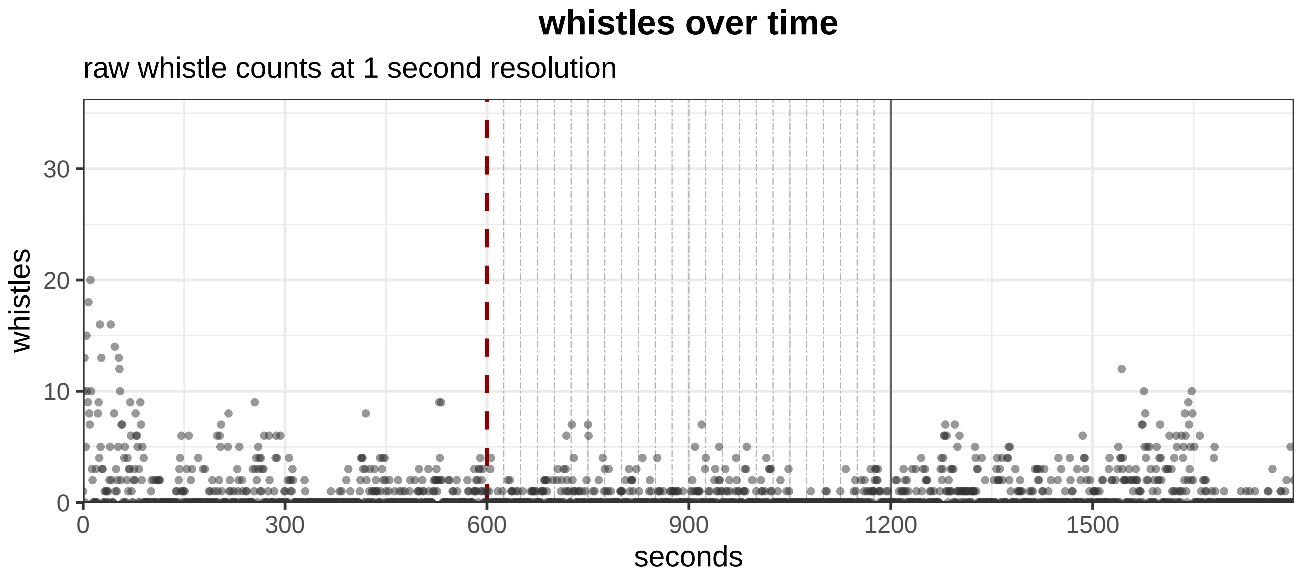

whistles over time
raw whistle counts at 1 second resolution
whistles
30
20
10
0
300
600
900
1200
1500
seconds

2021_03

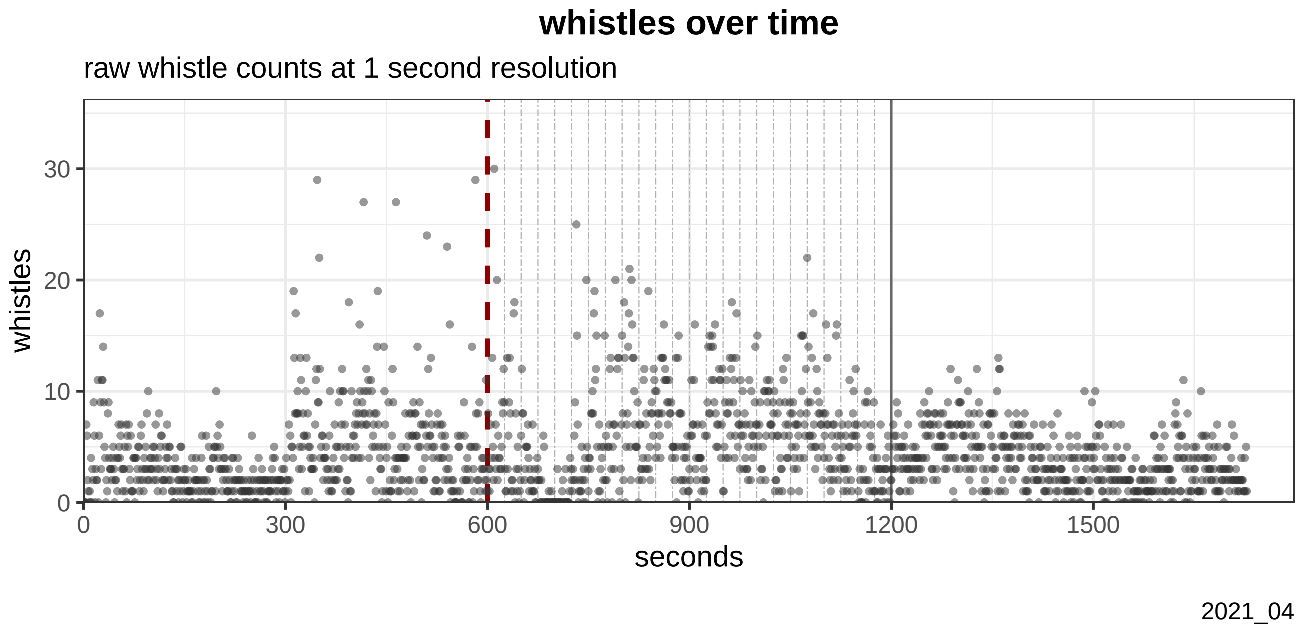

whistles over time
raw whistle counts at 1 second resolution
whistles
30
20
10
0
0
300
600
900
1200
1500
seconds
2021_04

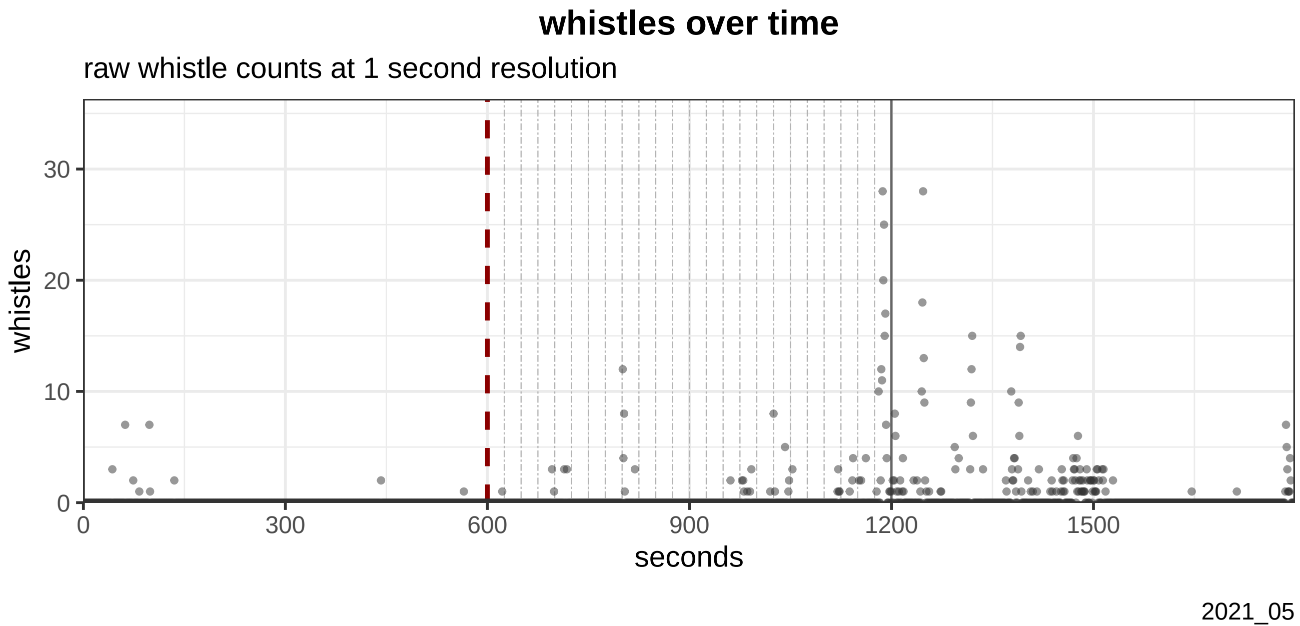
whistles over time
raw whistle counts at 1 second resolution
whistles
30
20
10
0
0
300
600
900
1200
1500
seconds
2021_05

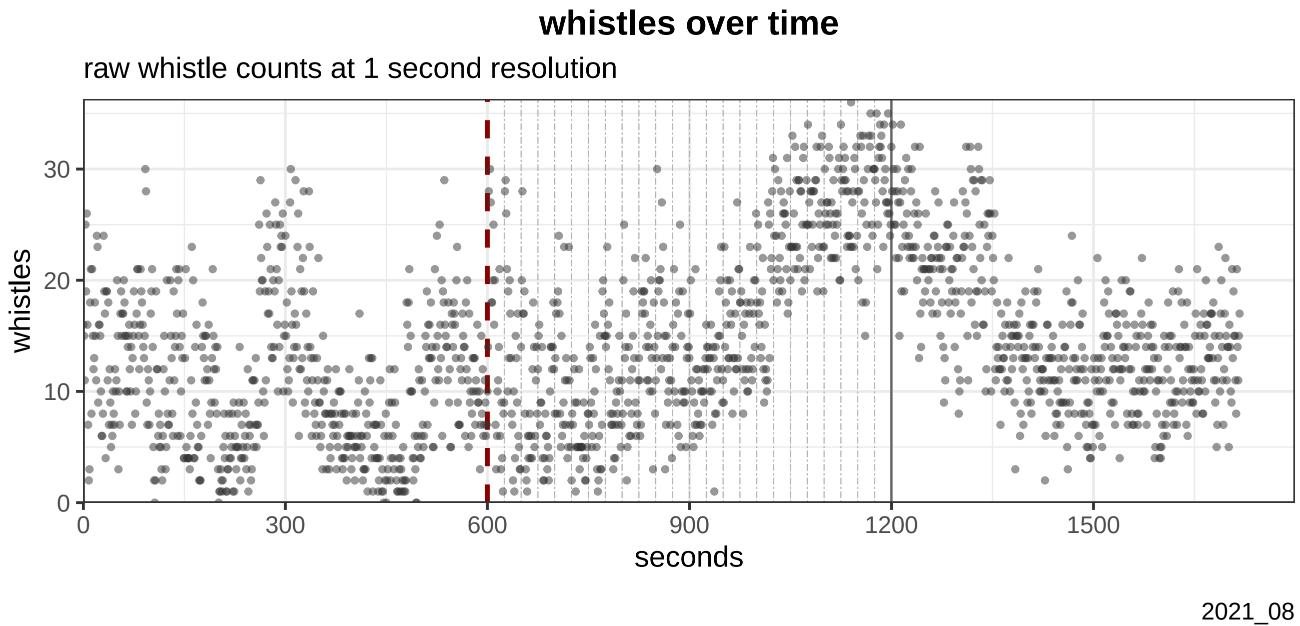
whistles over time
raw whistle counts at 1 second resolution
whistles
seconds
0
10
20
30
0
300
600
900
1200
1500
2021_08

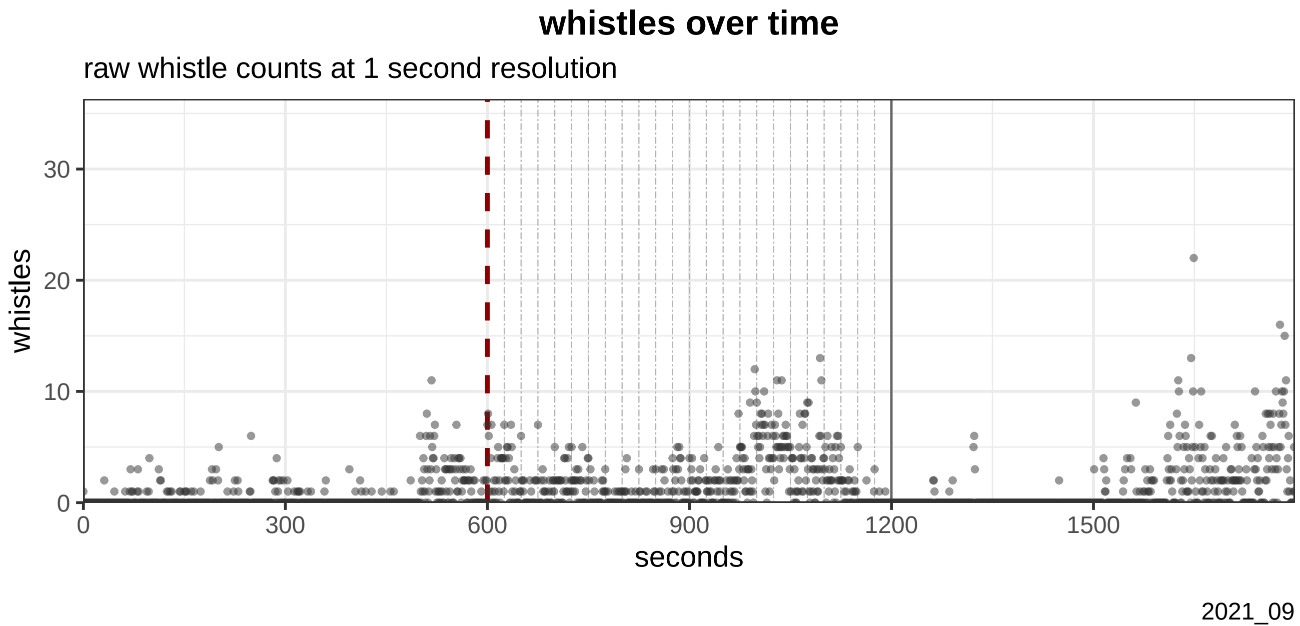

whistles over time
raw whistle counts at 1 second resolution
whistles
30
20
10
0
0
300
600
900
1200
1500
seconds
2021_09

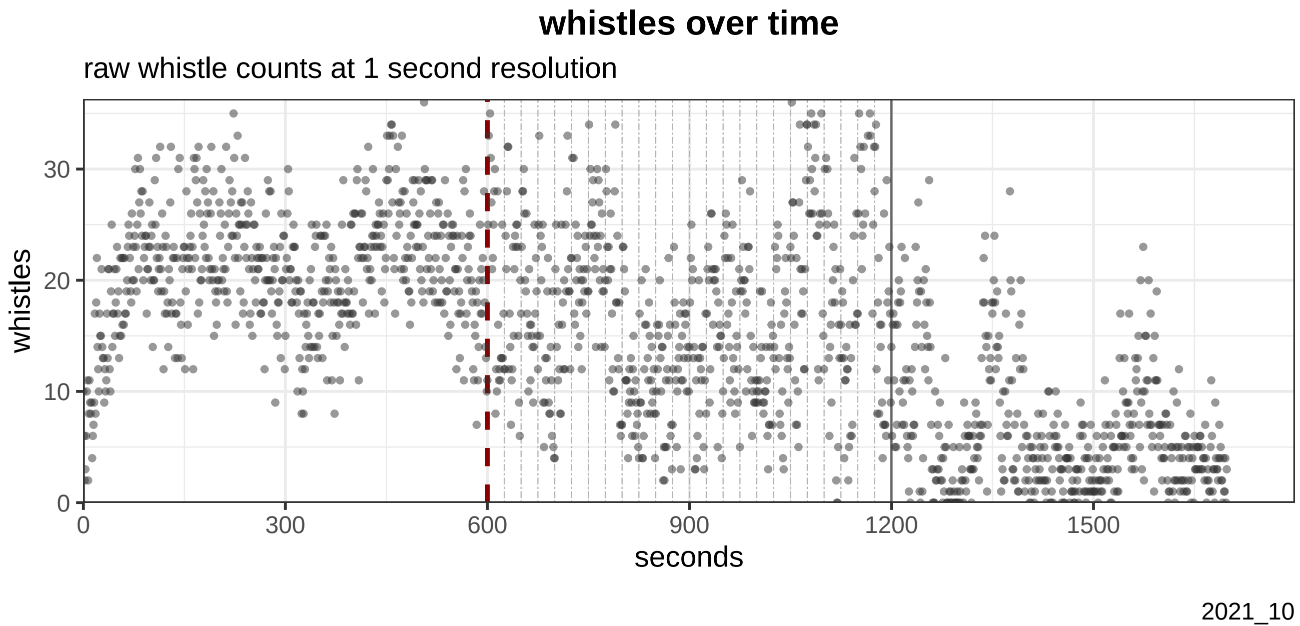

whistles over time
raw whistle counts at 1 second resolution
whistles
30
20
10
0
0
300
600
900
1200
1500
seconds
2021_10

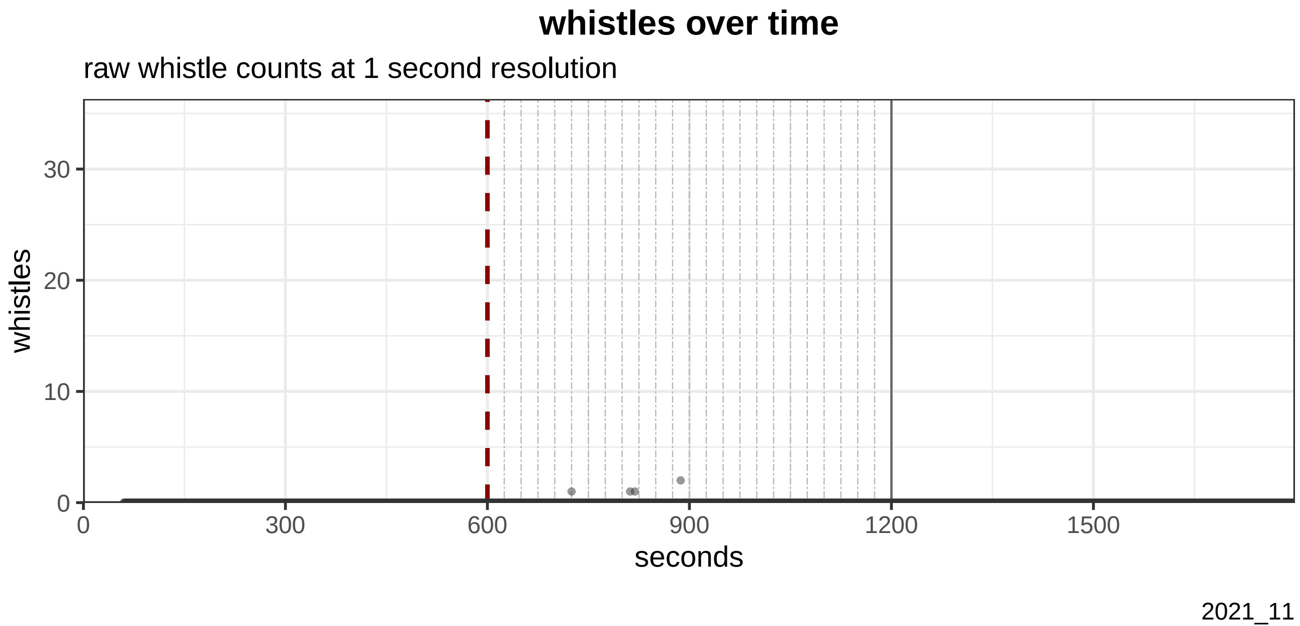

whistles over time
raw whistle counts at 1 second resolution
whistles
30
20
10
0
0
300
600
900
1200
1500
seconds
2021_11

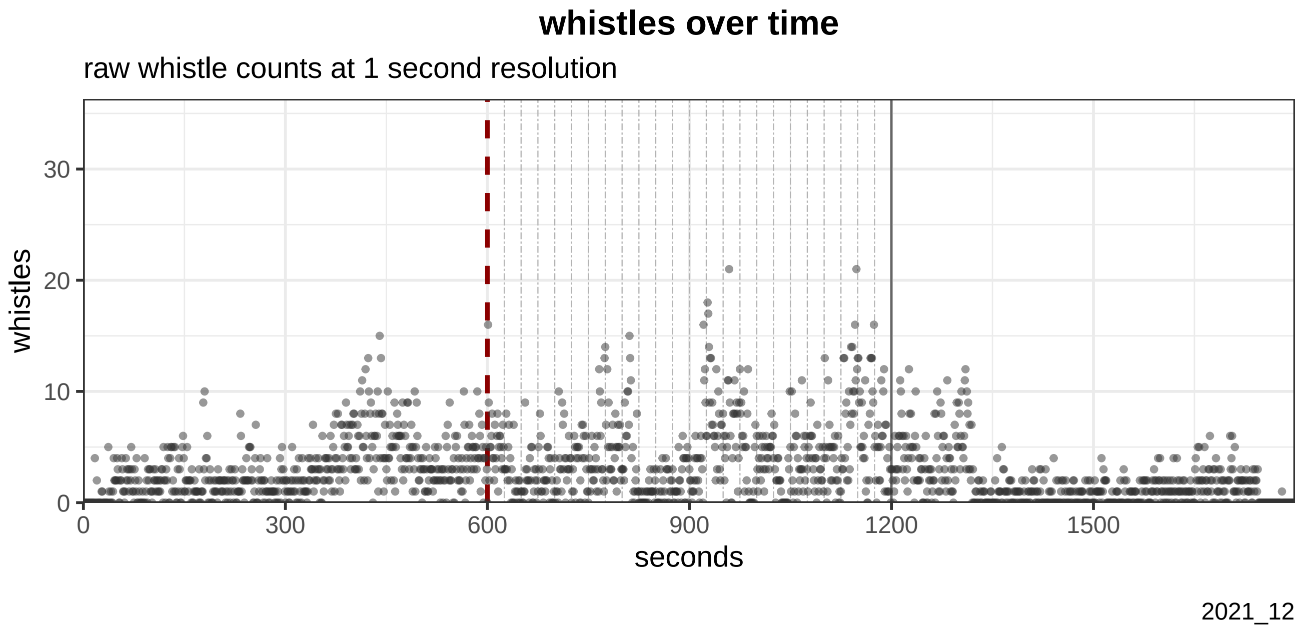

whistles over time
raw whistle counts at 1 second resolution
whistles
30
20
10
0
0
300
600
900
1200
1500
seconds
2021_12

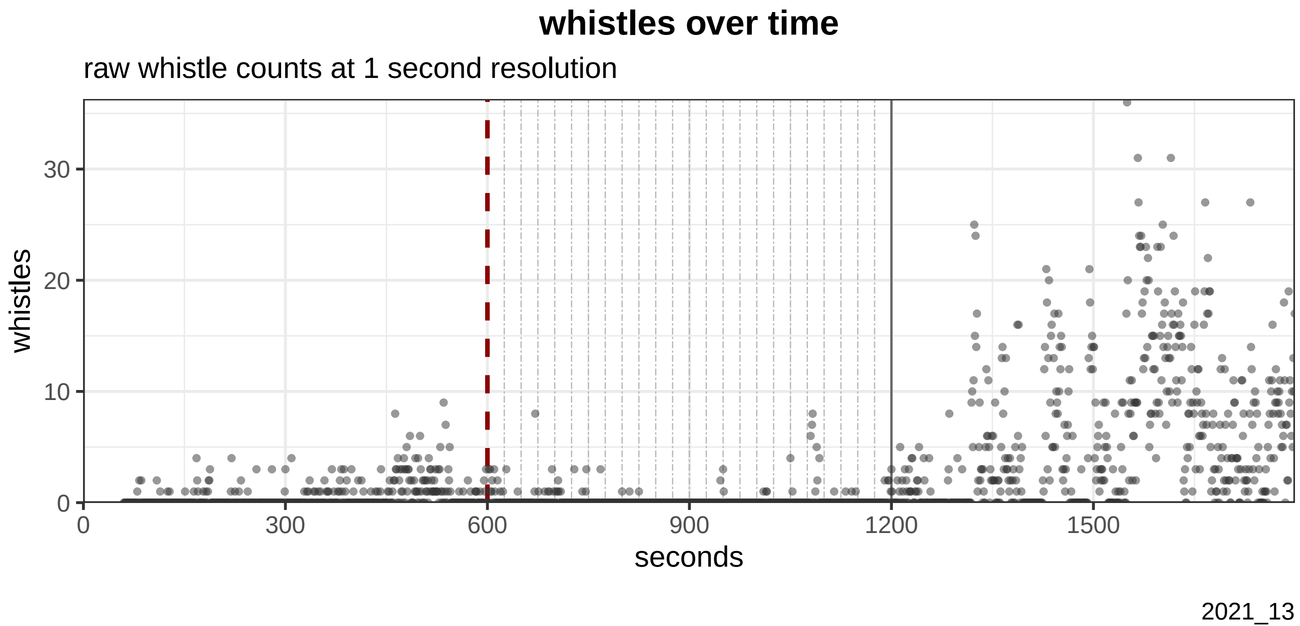

whistles over time
raw whistle counts at 1 second resolution
whistles
30
20
10
0
0
300
600
900
1200
1500
seconds
2021_13
