# Supplementary figures and images for: Common dolphin whistle responses to experimental mid-frequency sonar
Source: PLoS One. 2024 Apr 26;19(4):e0302035. doi: 10.1371/journal.pone.0302035 (PMC11051594; doi:10.1371/journal.pone.0302035)

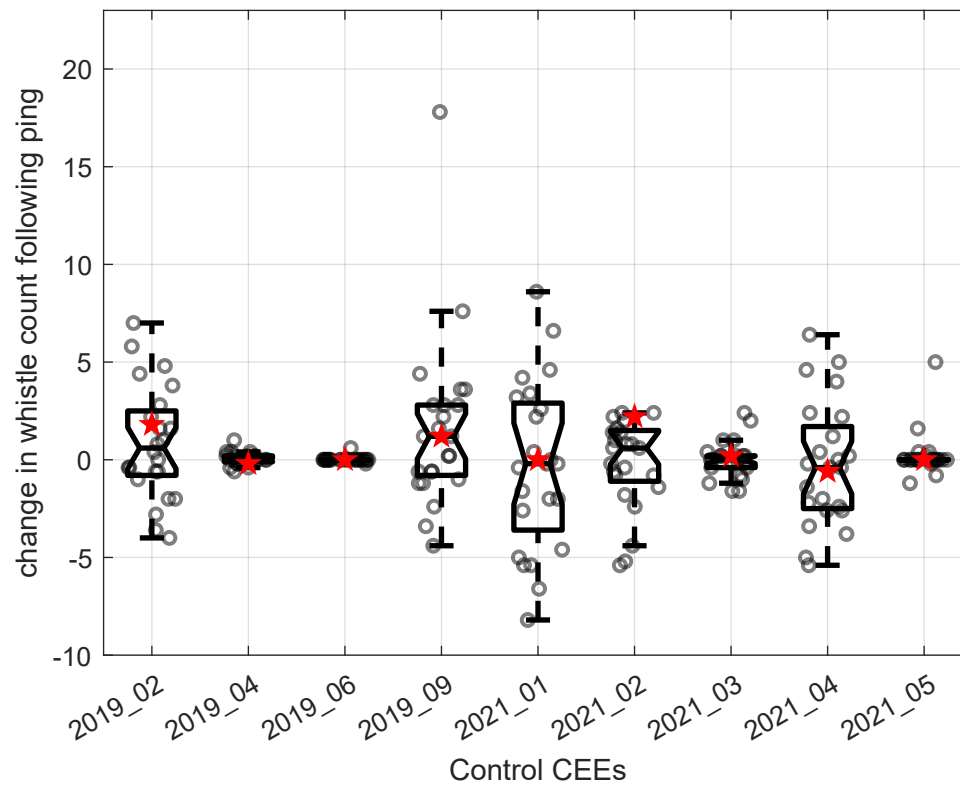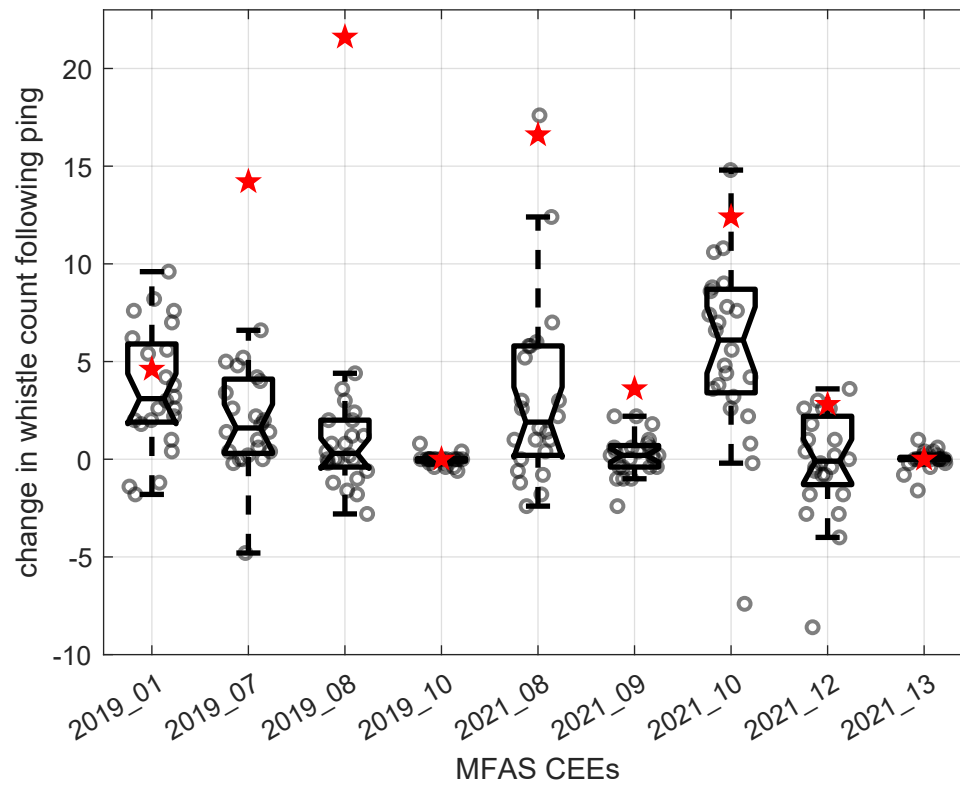

Supplement: S3 Fig — Boxplot shows median, 25th, and 75th percentiles, with raw whistle count changes as open gray circles. The change following the first ping is shown as a red star. (PDF) [file pone.0302035.s003.pdf]
