# Supplementary material for: Common dolphin whistle responses to experimental mid-frequency sonar
Source: PLoS One. 2024 Apr 26;19(4):e0302035. doi: 10.1371/journal.pone.0302035 (PMC11051594; doi:10.1371/journal.pone.0302035)
Supplement: S1 File — The main manuscript presents methods and results for analyzing common dolphin vocal response to simulated mid-frequency sonar, pooling data from all CEEs. We acknowledge that some readers may be interested in additional information about each of the 19 CEEs (10 MFAS exposures and nine controls), so we have included additional methods and results for each CEE. (PDF) [file pone.0302035.s004.pdf]

Methods

In the CEE-by-CEE analysis, potential differences between controls vs. MFAS CEEs were not compared, but rather, comparisons were made within individual CEEs between the pre-exposure vs. the exposure period. Like the pooled response analysis, the CEE-by-CEE analysis was conducted at three scales using three different analysis window sizes: 10 minutes (the entire pre-exposure period compared to the entire exposure period), 20 seconds (changes between pairs of 20-sec duration bins), and 5 seconds (changes between pairs of 5-sec duration bins).

Differences in pre-exposure vs exposure period whistle counts: 10-minute time scale

Similar to the aggregate model approach presented in the main manuscript, we used a Generalized Linear Mixed Model (GLMM) approach. We evaluated raw whistle counts (per second), *count*, as a function of experimental period (either pre or exposure), *period*, and the distance from the closest buoy to the focal group (interpolated every second), *buoyDistance*, by fitting a negative binomial model using R package `glmmTMB`. This is a suitable approach for count data that are overdispersed relative to a Poisson distribution (1). Of the two negative binomial fits available, the fit with the lowest AIC was chosen on a CEE-by-CEE basis. To account for temporal autocorrelation in this time series dataset, we also included a covariance structure (*ar1(time)*) which improved model performance.

$$count \sim period + buoyDistance + ar1(time) \qquad (S1)$$

One of the 10 MFAS experiments had to be excluded from this analysis (and all remaining within CEE analyses) due to an overall low (near zero) rate of whistle production throughout the experiment (CEE 2021_11). Note that this analysis compares whether the animals changed their average whistle production per second before and after the treatment, treating each second as an independent data point. This is an attempt to judge individual trials as having significant effects. However, it is important to remember that each CEE has only one exposure sequence and therefore significant results could be in response to another co-occurring event (e.g. natural

behavioural state change). Only the grouped analysis in the main manuscript accounts for such accidental variation by pooling CEEs into a larger sample size.

Differences in whistle counts in sequential 20-second time bin pairs

To characterize changes in whistle production over a shorter time window, we compared whistle counts 20 seconds before and 20 seconds after each ping (n = 24 1-second pings total over a 10-minute experimental period, ~25 seconds between each ping) and then used linear regression to model the magnitude of these changes between bin pairs as a function of experimental period. We selected this time window to capture sustained variation in whistling behavior within a single ping cycle without overlap between cycles.

We first calculated the mean number of whistles per second (whistle count) in the 20 seconds before and 20 seconds after each ping. Differences between these two subsequent time bins were calculated by subtracting the mean whistle count for the first bin from the mean whistle count of the second bin. The first ping started at time 0, the second ping at time 25 seconds, and so on. We repeated this process for the pre-exposure period to enable the assessment of the potential impacts of the MFAS playback signal. Because no actual pings were present in the pre-exposure period, the pairs of bins were centered around the analogous time to when pings occurred in the exposure period (i.e., 25 sec, 50 sec, 75 sec, etc for the full 10-minute pre-exposure period). To enable a post-hoc comparison across experiment types (control vs simulated MFAS exposure) we applied the same approach to controls using the times at which pings would have occurred (ghost pings) as the midpoint around which the bins were placed.

These paired bin differences served as the response variable, *binDiffs*, and the explanatory variable was a categorical variable *period*, either pre-exposure or exposure. We observed heteroscedasticity in the raw data; whistle variance varied with average whistle count. We thus applied a variance structure that allowed variance in whistle count to vary exponentially with average whistle count using the R package `nlme`.

$$binDiffs \sim period + \in \qquad (S2)$$

Each CEE (both controls and MFAS exposures) was modeled individually. We then assessed how often the CEE period significantly predicted differences in whistle count for both the controls and CEEs.

Differences in whistle counts in subsequent 5-second time bin pairs

The same analysis and linear regression modeling approach for 20-second duration subsequent bins was applied but using pairs of sequential bins of only 5 seconds duration. During the exposure period, these bins were centered around the pings. During the pre-exposure period and the exposure period for controls, these bins were centered around the time at which pings would have occurred. Again, each CEE was modeled individually, and ad-hoc comparisons were made across CEEs.

Results

Differences in whistle count at the 10-minute scale

Of the 18 CEEs that had sufficient whistle detections to be modeled, 7 had a significant difference in whistle count between the pre-exposure and exposure period – 5 of the seven significant differences were MFAS exposures, and 2 were controls. Of the 9 MFAS CEEs, 5 showed a significant difference in whistle count between CEE experimental periods, and 4 did not. Those with significant differences varied in the level and direction of response.

S1 Table. Results of GLMM analysis of whistle count as a function of experimental period and buoy distance for each CEE individually.

CEE	CEE Type	*period* estimate	*period* pvalue	*buoyDistance* estimate	*buoyDistance* pvalue
2019_01	MFAS	1.0978	0.0000108*	0.000496	0.75982
2019_02	Control	0.3171	0.6329465	-0.0029648	0.0369*
2019_04	Control	-0.4365	0.4713107	-0.0012041	0.40453
2019_06	Control	-1.27	0.2326034	-0.0027367	0.1435
2019_07	MFAS	0.826	0.1955801	-0.0017367	0.01269*
2019_08	MFAS	-0.4248	0.3157593	-0.0012726	0.29314

2019_09	Control	0.3337	0.4489849	-0.0016191	0.08367
2019_10	MFAS	1.0946	0.6294804	-0.0001284	0.96733
2021_01	Control	0.8132	0.359100	-0.00397	0.029110*
2021_02	Control	0.5136	0.026200*	-0.0005	0.283900
2021_03	Control	-0.5739	0.026200*	0.000776	0.489900
2021_04	Control	0.2873	0.232400	-0.00032	0.523500
2021_05	Control	2.0624	0.032640	0.004451	0.107800
2021_08	MFAS	0.4533	0.007578*	0.00057	0.408000
2021_09	MFAS	1.505	0.005311*	-0.0019	0.329500
2021_10	MFAS	-0.2053	0.010980*	-0.00041	0.171900
2021_12	MFAS	0.4103	0.404600	-0.00018	0.889200
2021_13	MFAS	-1.7524	0.000001*	-0.00396	0.000099*

*significance at the $p < 0.05$ level

Differences in whistle count at the 20-second scale

No CEEs showed a significant relationship in the change in whistle count between two sequential bins of 20 second duration and the experimental period.

S2 Table. Results of linear regression analysis of the short-duration difference in whistle count between sequential 5 second paired bins, as a function of experimental period, for each CEE individually.

CEE	CEE Type	*period* estimate	*period* pvalue
2019_01	MFAS	1.01689	0.1810
2019_02	Control	0.25667	0.6388
2019_04	Control	-0.01827	0.6181
2019_06	Control	0.04426	0.3751
2019_07	MFAS	0.20424	0.7420
2019_08	MFAS	0.03803	0.9040
2019_09	Control	0.67586	0.4569

2019_10	MFAS	-0.01052	0.3234
2021_01	Control	0.31829	0.5319
2021_02	Control	0.20341	0.7258
2021_03	Control	0.03467	0.8109
2021_04	Control	-0.34744	0.5421
2021_05	Control	-0.01071	0.6308
2021_08	MFAS	1.52019	0.0944
2021_09	MFAS	0.09414	0.5128
2021_10	MFAS	0.58794	0.6578
2021_12	MFAS	-0.08095	0.8409
2021_13	MFAS	-0.01812	0.7481

*significance at the $p < 0.05$ level

Differences in whistle count at the 5-second scale

There was a significant effect of period on the differences in whistle count between two sequential 5 second bins in 5 of 18 CEEs; all 5 significant CEEs were MFAS exposure CEEs. All 9 controls were non-significant. The effect in all 5 significant MFAS CEEs was positive, indicating an increase in whistle count in the second of the 5 second bins. Effect sizes ranged from 0.5 to 5.7 times greater whistle count in the second bin in bin pairs during the exposure period compared to the pre-exposure period.

S3 Table. Results of linear regression analysis of the short-duration difference in whistle count between sequential 5 second paired bins, as a function of experimental period, for each CEE individually.

CEE	CEE Type	*period* estimate	*period* pvalue
2019_01	MFAS	3.2420	0.000012*
2019_02	Control	0.5070	0.4839
2019_04	Control	0.0225	0.6345
2019_06	Control	-0.0013	0.9774

2019_07	MFAS	1.6920	0.005099*
2019_08	MFAS	0.3218	0.3899
2019_09	Control	1.4060	0.07018
2019_10	MFAS	0.0000	1.0000
2021_01	Control	0.070529	0.8475
2021_02	Control	0.193609	0.7284
2021_03	Control	-0.13344	0.4807
2021_04	Control	-0.56681	0.3358
2021_05	Control	0.001388	0.8491
2021_08	MFAS	2.947619	0.046380*
2021_09	MFAS	0.508054	0.049320*
2021_10	MFAS	5.727571	0.000001*
2021_12	MFAS	0.09499	0.8309
2021_13	MFAS	-0.03488	0.5048

*significance at the $p < 0.05$ level

References

1. Nikoloulopoulos AK, Karlis D. On modeling count data: a comparison of some well-known discrete distributions. *JSCS.* 2008;78(3): 437-57.
