# Supplementary material for: Common dolphin whistle responses to experimental mid-frequency sonar
Source: PLoS One. 2024 Apr 26;19(4):e0302035. doi: 10.1371/journal.pone.0302035 (PMC11051594; doi:10.1371/journal.pone.0302035)
Supplement: Attachment — Submitted filename: Casey et al. PLOS ONE comments.pdf [file pone.0302035.s005.pdf]

PLOS ONE

Common dolphin whistle response to experimental mid-frequency sonar

--Manuscript Draft--

**Manuscript Number:**	PONE-D-23-38783
**Article Type:**	Research Article
**Full Title:**	Common dolphin whistle response to experimental mid-frequency sonar
**Short Title:**	Dolphin acoustic response to sonar
**Corresponding Author:**	Caroline Casey<br>UCSC: University of California Santa Cruz<br>Santa Cruz, UNITED STATES
**Keywords:**	Bioacoustics: Common dolphins; (Delphinus delphis); (Delphinus bairdii); Behavioral response study; Navy sonar; Acoustic behavior
**Abstract:**	Millions of oceanic dolphins are exposed and presumably affected by military sonar annually; however empirically measuring its impact on the behavior of free-ranging dolphins has proven logistically challenging. Additionally, baseline variability or the frequency of vocal state-switching during undisturbed conditions is lacking among social oceanic delphinids, making it difficult to attribute changes in vocal behavior to anthropogenic disturbance. Using a network of drifting acoustic buoys and a three-phased controlled exposure experiment, we investigated the effects of mid-frequency (3-4 kHz) active sonar (MFAS) on whistle production in short-beaked (Delphinus delphis) and long-beaked common dolphins (D. bairdii) in southern California. Given the complexity of acoustic behavior exhibited by these group-living species, we conducted our response analysis over varying temporal windows (10 min – 5 sec) to describe both longer-term and instantaneous changes in sound production. We found that common dolphins exhibited an acute and dramatic change in acoustic behavior in the 5-seconds following exposure to experimental Navy sonar. In some cases, this response was sustained throughout repeated exposures to sonar in the experiment, suggesting that dolphins may not habituate to this kind of disturbance. These results suggest that common dolphins exhibit a brief, yet dramatic acoustic response to MFAS, and highlights how different sized temporal windows – tuned to key aspects of baseline vocal behavior as well as experimental parameters related to MFAS exposure – impacts the detection of behavioral responses. We suggest that future work with oceanic delphinid species explore baseline vocal rates a-priori and use information on the rate of change in vocal behavior to inform the analysis time-window over which behavioral responses are measured.
**Order of Authors:**	Caroline Casey
	Selene Fregosi
	Julie Oswald
	Vincent Janik
	Fleur Visser
	Brandon Southall
**Additional Information:**	
**Question**	**Response**
**Financial Disclosure**<br><br>Enter a financial disclosure statement that describes the sources of funding for the work included in this submission. Review the submission guidelines for detailed requirements. View published research articles from *PLOS ONE* for specific	Yes

Powered by Editorial Manager® and ProduXion Manager® from Aries Systems Corporation

examples.<br><br>This statement is required for submission and **will appear in the published article** if the submission is accepted. Please make sure it is accurate.<br><br>**Funded studies**<br>Enter a statement with the following details:<br>• Initials of the authors who received each award<br>• Grant numbers awarded to each author<br>• The full name of each funder<br>• URL of each funder website<br>• Did the sponsors or funders play any role in the study design, data collection and analysis, decision to publish, or preparation of the manuscript?<br><br>Did you receive funding for this work?	
Please add funding details.<br>as follow-up to "**Financial Disclosure**<br><br>Enter a financial disclosure statement that describes the sources of funding for the work included in this submission. Review the submission guidelines for detailed requirements. View published research articles from *PLOS ONE* for specific examples.<br><br>This statement is required for submission and **will appear in the published article** if the submission is accepted. Please make sure it is accurate.<br><br>**Funded studies**<br>Enter a statement with the following details:<br>• Initials of the authors who received each award<br>• Grant numbers awarded to each author<br>• The full name of each funder<br>• URL of each funder website<br>• Did the sponsors or funders play any role in the study design, data collection and analysis, decision to publish, or preparation of the manuscript?	Funding for this project was provided by the U.S. Navy's Office of Naval Research (Award Numbers N000141713132, N0001418IP-00021, N000141712887, N000141912572).

Did you receive funding for this work?"	
Please select the country of your main research funder (please select carefully as in some cases this is used in fee calculation).<br>as follow-up to "**Financial Disclosure**<br><br>Enter a financial disclosure statement that describes the sources of funding for the work included in this submission. Review the submission guidelines for detailed requirements. View published research articles from *PLOS ONE* for specific examples.<br><br>This statement is required for submission and **will appear in the published article** if the submission is accepted. Please make sure it is accurate.<br><br>**Funded studies**<br>Enter a statement with the following details:<br>• Initials of the authors who received each award<br>• Grant numbers awarded to each author<br>• The full name of each funder<br>• URL of each funder website<br>• Did the sponsors or funders play any role in the study design, data collection and analysis, decision to publish, or preparation of the manuscript?<br><br>Did you receive funding for this work?"	UNITED STATES - US
**Competing Interests**<br><br>Use the instructions below to enter a competing interest statement for this submission. On behalf of all authors, disclose any competing interests that could be perceived to bias this work—acknowledging all financial support and any other relevant financial or non-financial competing interests.	The authors have declared that no competing interests exist.

This statement is required for submission and **will appear in the published article** if the submission is accepted. Please make sure it is accurate and that any funding sources listed in your Funding Information later in the submission form are also declared in your Financial Disclosure statement.<br><br>View published research articles from *PLOS ONE* for specific examples.<br><br>**NO authors have competing interests**<br><br>Enter: *The authors have declared that no competing interests exist.*<br><br>**Authors with competing interests**<br><br>Enter competing interest details beginning with this statement:<br><br>*I have read the journal's policy and the authors of this manuscript have the following competing interests: [insert competing interests here]*<br><br>* typeset	
**Ethics Statement**<br><br>Enter an ethics statement for this submission. This statement is required if the study involved:<br><br>• Human participants<br>• Human specimens or tissue<br>• Vertebrate animals or cephalopods<br>• Vertebrate embryos or tissues<br>• Field research<br><br>Write "N/A" if the submission does not require an ethics statement.<br><br>General guidance is provided below. Consult the submission guidelines for detailed instructions. **Make sure that all information entered here is included in the Methods section of the manuscript.**	Animal research was conducted under authorization of the United States National Marine Fisheries Service marine mammal research permits 19116 and 19091.

Powered by Editorial Manager® and ProduXion Manager® from Aries Systems Corporation

**Format for specific study types**<br><br>**Human Subject Research (involving human participants and/or tissue)**<br>• Give the name of the institutional review board or ethics committee that approved the study<br>• Include the approval number and/or a statement indicating approval of this research<br>• Indicate the form of consent obtained (written/oral) or the reason that consent was not obtained (e.g. the data were analyzed anonymously)<br><br>**Animal Research (involving vertebrate animals, embryos or tissues)**<br>• Provide the name of the Institutional Animal Care and Use Committee (IACUC) or other relevant ethics board that reviewed the study protocol, and indicate whether they approved this research or granted a formal waiver of ethical approval<br>• Include an approval number if one was obtained<br>• If the study involved *non-human primates*, add *additional details* about animal welfare and steps taken to ameliorate suffering<br>• If anesthesia, euthanasia, or any kind of animal sacrifice is part of the study, include briefly which substances and/or methods were applied<br><br>**Field Research**<br><br>Include the following details if this study involves the collection of plant, animal, or other materials from a natural setting:<br>• Field permit number<br>• Name of the institution or relevant body that granted permission	
**Data Availability**<br><br>Authors are required to make all data underlying the findings described fully available, without restriction, and from the time of publication. PLOS allows rare exceptions to address legal and ethical concerns. See the PLOS Data Policy and FAQ for detailed information.	Yes - all data are fully available without restriction

A Data Availability Statement describing where the data can be found is required at submission. Your answers to this question constitute the Data Availability Statement and **will be published in the article**, if accepted.<br><br>**Important:** Stating 'data available on request from the author' is not sufficient. If your data are only available upon request, select 'No' for the first question and explain your exceptional situation in the text box.<br><br>Do the authors confirm that all data underlying the findings described in their manuscript are fully available without restriction?	
**Describe where the data may be found in full sentences. If you are copying our sample text, replace any instances of XXX with the appropriate details.**<br><br>• If the data are **held or will be held in a public repository**, include URLs, accession numbers or DOIs. If this information will only be available after acceptance, indicate this by ticking the box below. For example: *All XXX files are available from the XXX database (accession number(s) XXX, XXX.).*<br>• If the data are all contained **within the manuscript and/or Supporting Information files**, enter the following: *All relevant data are within the manuscript and its Supporting Information files.*<br>• If neither of these applies but you are able to provide **details of access elsewhere**, with or without limitations, please do so. For example:<br><br>*Data cannot be shared publicly because of [XXX]. Data are available from the XXX Institutional Data Access / Ethics Committee (contact via XXX) for researchers who meet the criteria for access to confidential data.*<br><br>*The data underlying the results presented in the study are available*	All exported raw whistle counts from PAMGuard and associated buoy distances for each playback experiment will be made available via Dryad data repository htt;://datadryad.org/.

from (include the name of the third party and contact information or URL).<br>• This text is appropriate if the data are owned by a third party and authors do not have permission to share the data.<br><br>* typeset	
Additional data availability information:	Tick here if the URLs/accession numbers/DOIs will be available only after acceptance of the manuscript for publication so that we can ensure their inclusion before publication.

Powered by Editorial Manager® and ProduXion Manager® from Aries Systems Corporation

Manuscript Click here to access/download;Manuscript;Manuscript.docx

Full title: Common dolphin whistle response to experimental mid-frequency sonar

Short title: Dolphin acoustic response to sonar

*Caroline Casey[a,b], *Selene Fregosi[a], Julie N. Oswald[c], Vincent M. Janik[c], Fleur Visser[d,e], Brandon Southall[a,b]

[a]Southall Environmental Associates, Inc., 9099 Soquel Drive, Aptos, CA 95003, USA

[b]Institute of Marine Sciences, University of California Santa Cruz, 115 McAllister Way, Santa Cruz, CA 95060, USA

[c]Sea Mammal Research Unit, Scottish Oceans Institute, School of Biology, University of St. Andrews, St. Andrews, KY16 8LB, United Kingdom

[d]Kelp Marine Research, 1624 CJ, Hoorn, the Netherlands

[e]Department of Coastal Systems, Royal Netherlands Institute for Sea Research, P.O. Box 59, 1790 AB Den Burg, Texel, the Netherlands

Corresponding author: Caroline Casey, cbcasey@ucsc.edu

Abstract

Millions of oceanic dolphins are exposed and presumably affected by military sonar annually; however empirically measuring its impact on the behavior of free-ranging dolphins has proven logistically challenging. Additionally, baseline variability or the frequency of vocal state-switching during undisturbed conditions is lacking among social oceanic delphinids, making it difficult to attribute changes in vocal behavior to anthropogenic disturbance. Using a network of drifting acoustic buoys and a three-phased controlled exposure experiment, we investigated the effects of mid-frequency (3-4 kHz) active sonar (MFAS) on whistle production in short-beaked (*Delphinus delphis*) and long-beaked common dolphins (*Delphinus bairdii*) in southern California. Given the complexity of acoustic behavior exhibited by these group-living species, we conducted our response analysis over varying temporal windows (10 min – 5 sec) to describe both longer-term and instantaneous changes in sound production. We found that common dolphins exhibited an acute and dramatic change in acoustic behavior in the 5-seconds following exposure to experimental Navy sonar. In some cases, this response was sustained throughout repeated exposures to sonar in the experiment, suggesting that dolphins may not habituate to this kind of disturbance. These results suggest that common dolphins exhibit a brief, yet dramatic acoustic response to MFAS, and highlights how different sized temporal windows – tuned to key aspects of baseline vocal behavior as well as experimental parameters related to MFAS exposure – impacts the detection of behavioral responses. We suggest that future work with oceanic delphinid species explore baseline vocal rates a-priori and use information on the rate of change in vocal behavior to inform the analysis time-window over which behavioral responses are measured.

Introduction

Sound production and reception play a critical role in the lives of cetaceans, aiding in important life-history events including maintenance of social relationships, coordination of group movement, foraging, and evasion of predators (1). Consequently, substantial effort has been directed toward describing cetacean acoustic behavior (2,3) and evaluating how it is impacted by human-generated disturbance (4-8). There are many sources of anthropogenic noise pollution (*e.g.*, ~~vessel noise, oil and gas exploration, construction and facilities maintenance, fisheries and aquaculture, military activity~~) which can have varying short and long-term impacts on marine mammal ~~health and behavior~~ (4, 9-11). Concentrated research efforts aimed at characterizing these impacts have led to the systematic development of acoustic exposure criteria, informing and improving effective management strategies for both regulators and industries (for ~~reviews on auditory and behavioral~~ criteria, see 12). Such ~~assessments and criteria~~ have also highlighted species and noise exposure contexts for which information is sparse or unavailable.

Among cetaceans, oceanic delphinids represent an important and logistically challenging group of species for which to evaluate the impact of anthropogenic noise on vocal behavior. These animals are highly soniferous, abundant, and often extremely gregarious (pods of > 500 individuals are common for some species). Sound production has been demonstrated to play a particularly important role in the regulation of social interactions and cohesion among group members (*e.g.*, 2,3). Oceanic delphinids are ubiquitous around some U.S. Navy operational areas where mid-frequency active sonar (MFAS; 1-10 kHz) is commonly used for submarine detection in training exercises ~~and warfare~~, resulting in associated large numbers of sonar exposures for these federally protected species. Some of the most powerful MFAS systems (*e.g.*, ~~SQS-53C~~)

use repeated pings with fundamental frequencies in the 3-4 kHz range, ping lengths of approximately 1-3 seconds, and effective source levels as high as 235 dB re 1μPa that may be transmitted for several minutes to hours at high duty cycles (13). Aside from the elevated background noise and potential disturbance that may result from these training exercises (*e.g.*, 14), MFAS signals overlap with the frequencies that oceanic dolphins commonly rely on for social sound (whistle) production. Delphinid whistles are narrowband tonal sounds with most of the acoustic energy concentrated below 20 kHz (14-16).

While MFAS has been linked to mass stranding events of cetaceans (13,17) and its effect on cetaceans has been experimentally evaluated in a handful of species (for a recent review, see 19) the effects of sonar on the acoustic behavior of oceanic delphinids have not been systematically explored. This is due in large part to the logistical challenges of applying previously developed methods used in other behavioral response studies of individual animals to large aggregations of dolphins. Much of the prior research on cetacean behavioral responses to noise has capitalized on the use of suction-cup attached motion-sensing and acoustic recording tags to characterize responses following controlled exposure to MFAS (21-23). Unfortunately, such tags are challenging to deploy and are easily shed by small dolphins due to the tag size relative to the smaller body surface of dolphins and high drag due to fast swimming speeds. Additionally, oceanic delphinids commonly occur in large groups that display remarkable coordination, making the collective vocal behavior of the group perhaps a more appropriate focus of analysis (20). Opportunistic passive acoustic studies relying on large, cabled hydrophone arrays have been used to quantify changes in vocal activity and thus as a proxy for presence of or absence of multiple animals before, during, and after exposure to MFAS (24,25). Such experiments are

valuable but require extensive, high-cost moored hydrophone arrays with restricted spatial coverage.

~~Findings from previous studies of acoustic responses to Navy sonar in oceanic delphinids include shifting specific frequency~~ components of whistle contours, increasing or decreasing calling rate, increasing call amplitude, and even mimicry of MFAS elements (14, 26-28). For example, tagged ~~orcas~~ (*Orcinus orca*) adjust the high-frequency component of their whistles during sonar exposure, and increase the number and amplitude of their calls following each ping (27). False killer whales *(Pseudorca crassidens)* appear to increase their whistle rate and produce more MFA-like whistles after exposure to simulated sonar (26).

One opportunistic study provided initial insights into the behavioral responses of some social oceanic delphinids to MFAS. Bottlenose dolphins (*Tursiops truncatus*), common dolphins (*Delphinus sp.*), Pacific white-sided dolphins (*Lagenorhynchus obliquidens*), and Risso's dolphins (*Grampus griseus*) ~~exposed to incidental~~ MFAS showed a cessation of vocalizations, an increase in the intensity of vocalizations, or a combination of both (14). Of all delphinid species, common dolphins displayed the widest range of responses, including changing their behavioral state or direction of travel when sonar stopped, increasing the intensity of vocalizations when sonar began, vocalizing very little or not at all during sonar, or a combination of these observations (14). These results are consistent with a more recent opportunistic evaluation of delphinid responses to an underwater ~~explosion~~, which showed that whistle rate, complexity, and frequency content all varied in response to the explosive event (30). Depending upon the frequency, intensity, and consistency of these kinds of noise exposures, such behavioral changes

could result in physiological consequences that impact overall population health (31). Unfortunately, information on baseline variability ~~or~~ the frequency of vocal state-switching during undisturbed conditions is lacking among social oceanic delphinids, making it challenging to interpret the responses observed. While opportunistic studies of delphinid acoustic responses to sonar are insightful, a detailed assessment under controlled experimental conditions is needed to understand the extent to which delphinids are impacted by this kind of disturbance.

Quantifying vocal behavior in these taxa is complicated by the fact that dolphin acoustic behavior is dynamic, variable, and influenced by a myriad of social and environmental factors (32). Measurements of acoustic behavior and how it changes in response to disturbance must be made across some predetermined interval of time. In previous cetacean behavioral response studies using a conventional controlled exposure experimental (CEE) design, this temporal window was often dictated by logistical limitations of the technology being used (*e.g.*, battery power of tags, the feasibility of continuous behavioral observations, etc.), or designed to match the duration of anthropogenic noise source being evaluated. However, averaging vocal behavior over long time windows may result in missing instantaneous or shorter duration responses that occur at the onset of exposure or at scales that are more biologically meaningful to the individuals exposed. One way to address this is to examine acoustic metrics computed over a variety of time windows to determine if and/or when we can attribute a change in vocal behavior to a known, controlled disturbance.

~~Here, we apply and compare different broad and fine-scale analytical approaches to analyzing~~ delphinid vocal behavior and effects of ~~controlled~~ MFAS on whistle production ~~using~~

~~experimental methods~~ in short-beaked (*Delphinus delphis*) and long-beaked common dolphins (*Delphinus bairdii*) in southern California. By assessing group-level vocal behavior across different time scales, we ~~aim~~ to:

1. Describe the variability in baseline vocal behavior of common dolphin aggregations during control conditions.

2. ~~Characterize the types of~~ vocal responses detected during a controlled exposure to MFAS ~~using broad and fine-scale temporal resolution~~.

Given the need to establish sampling regimes that can be applied and compared across studies, ~~our objective is~~ to provide an informative framework for assessing the complex acoustic behavior exhibited by group-living species. We highlight how using different sized temporal windows – tuned to key aspects of baseline vocal behavior as well as experimental parameters related to MFAS exposure – impacts the detection of behavioral responses.

Methods

A. General Methods

~~Controlled exposure experiments (CEEs)~~ were conducted with two ~~species~~ of common dolphins as part of a broader effort aimed at quantifying group-level responsiveness of oceanic delphinids to military sonar using CEEs. Common dolphins occur as two separate species within our study area (33), around Santa Catalina Island located off the coast of southern California, USA. Since they regularly occur in mixed~~-species~~ groups, we pooled data for the two species to describe baseline vocal data and included species as a potential explanatory variable in our models. The project ~~integrates~~ multiple different data streams, including shore-based tracking of dolphin

schools, passive acoustics to record vocal activity, and photogrammetry to measure fine-scale behavior (34). This work was conducted between 2017-2021. We chose our study area because it lies near the Southern California Offshore Range (SCORE) – a tactical training area for the U.S. Navy Pacific Fleet located off the west side of San Clemente Island – where animals regularly encounter the types of signals we used in our experiment.

CEEs were composed of three discrete phases: pre-exposure (baseline), exposure using intermittent simulated MFAS signals, and post-exposure. In a subset of experiments, no MFAS signals were broadcast during the exposure phase, serving as controls. For details about the experimental source and sound source characteristics – including calculations of received levels – see Durban et al. 2022 (34). Each experimental phase was 10 minutes in duration. During exposure phases, MFAS 'pings' of [illegible] in duration consisting of three tonal and frequency modulated elements between 3.5-4 kHz were transmitted (see: Southall et al. 2012 (35) for additional details on the sound source and signal parameters). Pings were emitted at a broadband source level of 212 dB re 1μPa RMS every 25 seconds, which is similar in repetition rate, duty cycle, and the absence of a ramped-up source level (as used in some previous MFAS CEEs) to some active Navy MFAS systems (*e.g.*, helicopter-dipped sonar systems). Up to 24 total pings were emitted per 10-minute exposure phase, provided that no permit-mandated shutdowns occurred for animals occurring within 200 m of the active sound source (this occurred in only one playback). The sound source was positioned relative to focal animal groups using noise propagation modeling to ensure received levels at focal animals occurred at maximum levels of 140-160 dB RMS.

For every CEE, species identity (based on differences in genetics, morphology and pigmentation) was determined using a combination of aerial images obtained from drone footage, genetic sequencing from biopsy samples, and visual observation. Additionally, group size was characterized by experienced shore-based observers using strong magnification binoculars or a binocular scope located at elevated locations (~70m) that enabled a broad overview of the research area (up to 20 km from shore). Shore-based tracking of animals could be conducted for groups up to 7 km from shore. Focal follows included estimation of low, best and high group size, the number of subgroups (defined as all individuals in closer proximity to each other than to other individuals in the area), the range of inter-individual spacings within subgroups, and distances between subgroups. These observations were taken continuously throughout the experiment at 2-minute intervals. For a full description of each of these methods see Visser et al., 2014 (36) and Durban et al., 2022 (34).

B. Acoustic Data Collection and Processing

1. Passive Acoustic Monitoring

Passive acoustic recordings were obtained from each target group of dolphins using three drifting, remote-deployed acoustic recording units. Up to three separate recording units were tactically positioned and recovered from a single small (~6 m) rigid-hull inflatable boat, with the objective of placing one recording unit within 500 m of the predicted trajectory of the dolphins during each CEE phase (Fig. 1). Each recording unit consisted of a surface buoy and flag with an underwater recorder suspended by 10 m of line. The recorder was either a SoundTrap ST300 (Ocean Instruments NZ, Auckland, New Zealand) or a SNAP Recorder (Loggerhead Instruments, Sarasota, FL, USA). Both recorded via a single omnidirectional calibrated

hydrophone (SoundTrap: integrated hydrophone, frequency response 0.02–60 kHz ± 3 dB, end-to-end sensitivity -178 dB re 1µPa/V; SNAP: HTI-96-MIN hydrophone, sensitivity -164 dB re 1µPa/V, frequency response 1.0-20 kHz ± 3 dB) which was suspended by a shock-mounted cable at a depth of 10 m. All recording units had a Global Positional System (GPS) tracking device (Trace, SPOT LLC, Chantilly, VA, USA) that recorded the location of the instrument once every minute (Fig. 1). Five-minute WAV files were continuously recorded at either 96 kHz sampling rate with 16-bit resolution (SoundTrap) or 44.1 kHz sampling rate with 16-bit resolution (SNAP).

Figure 1. A schematic representation of the placement of acoustic recorders (see inset for floating acoustic recording unit) relative to the track of the focal group of dolphins. The first buoy is placed within 500m of the animals during the pre-exposure, the second during the exposure, and the third during the post-exposure period. The sound source is located at approximately 1 km from the dolphins at the onset of the exposure period. The dashed line with associated arrows represents the movement path of the focal group. Note the presence of the shore station positioned on land.

To evaluate which PAM recording unit was closest to the focal group given the dolphins' frequently unpredictable course, the relative proximity of each hydrophone to the animals was determined *post-hoc*. The animals' location was known from an associated octocopter drone flight (APO-42, Aerial Imaging Solutions) centered over the focal group (see 34 for details). The relative distances (in meters) between the focal group (from the drone's GPS) and each recorder (from their flag-mounted GPS units) were estimated for every minute of the 30-minute

experiment using the Haversine formula and linear interpolation in a custom MATLAB script (Mathworks, Natick, MA, USA, Fig. 2). Recordings from the buoy closest to the focal group at 1-minute intervals were used for all subsequent analyses. Any recorders that exceeded 1600 meters from the focal group (even if they were the closest recorder deployed) were excluded. This threshold was selected based on a previous assessment of detection ranges of playbacks of odontocete whistles (10-20 kHz) by bottom hydrophones in southern California, which demonstrated a 95% probability of detection of a 135 dB re 1 μPa dolphin whistle at 1600 meters with an SNR of 2.2 dB (37). This assessment was supported by the drop-off in whistle amplitude observed in the spectrograms (*post-hoc*) when any buoy surpassed 1600-1800 m distance from the focal group (Fig. 2).

Figure 2. Spectrograms from each of the three recorders strategically placed on the track-line of a moving group of dolphins. The solid white line represents the distance between the recorder and the drone flight centered over the focal group (units on the right y-axis), the red dashed vertical lines denote the experimental period, and the horizontal white dashed line marks the 1600 m threshold. Estimates of the relative distance between the focal group and each of the recorders were produced every minute of the 30-minute experiment. The bottom panel shows the number of whistles detected on the closest recorder using the PAMGuard Whistle and Moan Detector. Times when the closest buoy switched is indicated by the blue dashed lines and associated blue numbers. MFAS pings denoted by the vertical lines on the spectrogram during the experimental period.

2. Quantifying whistle production and variability

While common dolphins are known to emit buzzes, echolocation clicks, and whistles, we focused our efforts on characterizing whistle production since they are the key signal for long distance communication and play a major role in group cohesion and coordination (2, 38). Extraction of whole whistle contours in high background noise with overlapping whistles is extremely challenging and results in high rates of missed detections, irrespective of methodology. To create a dataset in which error rates were kept constant across different phases of the experiment, we used the Whistle and Moan Detector (WMD) module in PAMGuard (v 2.01.05, 39). The WMD deals with uncertainties by only detecting parts of whistles that clearly stand out above noise using standardized settings across extractions. It is important to note that this often leads to a fragmentation of whistles with one whistle being split into several independent sections. Thus, whistle counts reported here are not comparable to ones obtained with whole whistle extraction in other studies. However, for our assessment of changes in vocal activity between different experimental phases, it was more important to keep error rates constant to allow for relative comparisons.

The WMD operates on the spectrogram output of the PAMGuard F[illegible] Engine module. We optimized settings for the FFT Engine to provide comparable frequency and temporal resolution of the calculated spectrograms across the two recorders and sampling rates. For the SoundTrap recorders, which had a sampling rate of 96 kHz, the FFT Engine module calculated spectrograms with an FFT length 1024, hop size 512, and a Hann window ~~providing~~ a frequency resolution of 93.75 Hz and time resolution of 10.67 ms. For the SNAP recorders, which had a sampling rate of 44.1 kHz, spectrograms were calculated with a Hann window, FFT length 512 and hop size 256 resulting in a frequency resolution of 86.13 and time resolution of 11.61 ms. The WMD was set

to detect whistles between 5 kHz and 20 kHz to exclude detection of the tonal sounds from the simulated mid-frequency sonar ~~playbacks~~ (below 5 kHz) and to standardize the upper detection limit across the two sampling rates and avoid any possible edge effects near the Nyquist frequency of the lower sampling rate. The detection threshold was set at 6.0 dB. Full WMD settings can be found in the supplementary materials (S1 Appendix). While the fundamental sonar tonals were excluded by the 5 kHz low-pass cut-off for detections, the high source level of the simulated MFAS resulted in the presence of harmonics in some of the recordings. All harmonics were manually annotated in PAMGuard Viewer using the Spectrogram Annotation module for later removal.

Whistles were exported from PAMGuard using the PAMGuard MATLAB tools (https://github.com/PAMGuard/PAMGuardMatlab) and R package `PAMPal` (40). MFAS harmonics were removed, and whistles were quantified at 1-second resolution. Because whistles are often longer than 1-second in duration, the total number of whistles starting within a 1-second bin was counted, providing a metric for whistle activity as detected whistles per second. For brevity, this is referred to as whistle count throughout the remainder of this manuscript.

C. Characterization of baseline vocal behavior

1. Overall whistle count

To assess common dolphin vocal behavior ~~in~~ control conditions, we calculated the mean and median whistle count per second for the full 30-minute experiment for each control CEE. These data were evaluated with reference to concurrent data collected ~~by experienced shore-based focal follow observations~~ that quantified group size.

2. Changepoint analysis

To describe the natural variability in vocal behavior during control conditions, we applied a changepoint analytical approach to the control CEE data collected for both common dolphin species. Change point detection is used to pinpoint times when the probability distribution of a time series changes (*i.e.*, vocal state changes). The aim is to identify times at which either the mean or variance deviates from the expected trends in the dataset and estimate the number and position of all changepoints. Effectively, this approach detects points in time when a significant change in whistle count occurs. First, a 5-second smoothing window was applied to the raw 1-second whistle count data. Then, changepoints in both mean whistle count and whistle count variance were detected using the 'changepoint' package in R version 2.2.4 (41). The "BinSeg" (Binary Segmentation) algorithm was used. This provided the number and locations of all state changes in both the mean and the variance of whistle count over the total 30-minute sampling period.

D. Assessing the impact of disturbance on vocal behavior

We employed a hierarchical approach to characterizing the types of vocal responses that might be detected during controlled exposure to MFAS using broad and fine-scale time windows (Fig. 3). All statistical analyses were carried out using R version 4.3.1 (R Core Team 2023).

Figure 3. Flowchart of methods implemented to assess changes in common dolphin vocal behavior during controlled exposure to MFAS. Methods include pre-processing of acoustic data to baseline vocal behavior analysis and hierarchical assessment of disturbance on vocal

behavior at four temporal scales. Alpha-numeric references corresponds to the sub-heading in the methods section.

1. Difference in changepoints by period across CEEs

We conducted a changepoint analysis on all CEEs (both controls and MFAS) to evaluate whether common dolphins change the frequency of vocal state switching as a result of exposure to MFAS. We used the same general method as in Section C.2 but quantified the number of changepoints in the 10-minute pre- and 10-minute exposure periods separately. Changepoints were detected for both the mean and variance of the whistle count data. The difference in the number of changepoints between the two periods was calculated and an unpaired t-test was used to evaluate any significant differences between controls and MFAS experiments.

2. Characterize the impact of MFAS exposure on whistle count: 10-minute time scale

To identify potential broad scale changes in whistle count in response to ~~a simulated MFAS exposure we pooled~~ all CEEs (both controls and MFAS exposures) ~~and analyzed them~~ using a Generalized Linear Mixed Model (GLMM) approach, implemented using R package `glmmTMB` (42). We modeled the absolute difference in median whistle count between the 10-minute pre-exposure period and the 10-minute exposure period (periodDiff) as a function of CEE type (ceeType; either control or simulated MFAS), a random identity variable (ceeNum), dolphin ~~species~~ (species), the best estimate of total group size from the shore based observers (groupSize), and the mean distance between the focal group and the closest buoy for the full CEE (buoyDistance). Using the absolute value for difference in median whistle count enabled us

to explore the magnitude of a potential response. We modeled the relationship using a negative binomial distribution which fit the count-type data after the transformation. Our full model was:

$$peridDiff \sim ceeType+ceeNum+species+groupSize+buoyDist$$

We used backward elimination and resulting AIC scores to select our final model ~~and present the results of the final model below~~.

3. Characterize the impact of MFAS exposure on whistle count: 20-second time scale

To characterize more instantaneous changes in whistle production in response to MFAS exposure, we compared dolphin whistle counts in the 20 seconds before and 20 seconds after each individual ping (n = 24 1-second pings per 10-minute experimental period, ~25 seconds between each ping) for both MFAS experiments and controls. We selected this time window to capture sustained variation in whistling behavior within a single ping cycle, without overlap between cycles. Differences between these two sequential time bins were calculated by subtracting the mean whistle count for the first bin from the mean whistle count of the second bin (Fig. 4B). The first ping started at time 0, the second ping at time 25 seconds, and so on. Because no actual pings were present in the control experiments, we calculated the change in whistle count surrounding 'ghost pings' ~~which were chosen to be timed~~ at the same time as when real pings would have occurred during a MFAS CEE.

Figure 4. Example plots of (A) raw whistle counts over time, (B) changes in whistle count between 20 second duration sequential bins, and (C) changes in whistle count between 5

second duration sequential bins, for the pre-exposure, exposure, and post-exposure period within one CEE. The dashed vertical red line indicates the onset of exposure, and the sequential gray dashed lines represent each ping within the exposure period. Pre-exposure and post-exposure periods were not included in modeling analysis but are presented here for reference.

Similar to the analysis at the 10-minute time scale, we used a ~~generalized linear mixed model~~ approach (using R package `glmmTMB`) to identify potential significant differences immediately following pings (*pingChange*) in MFAS experiments compared to controls where no pings were present. In addition to the previous fixed effects included at the 10-minute scale (*ceeType, ceeNum, species, buoyDistance, and groupSize*), we also included median whistle count per second for the entire experimental period to account for the varied baseline whistling activity across CEEs (*medWhist*).

$$pingChange \sim ceeType + ceeNum + species + groupSize + buoyDist + medWhist$$

Binned ping change data were generally normally distributed, but were zero inflated, so we used a Gaussian distribution for the primary model and additionally modeled the zero inflation as a function of median whistle count (*medWhist*). We used backward elimination, ΔAIC, and analysis of variance (ANOVA) to select the best model.

4. Characterize the impact of MFAS exposure on whistle count: 5-second time scale

We repeated the analysis conducted in section D3, but over a shorter 5 second time window to investigate potential shorter duration changes immediately following pings (Fig. 4C).

Similar to the above analysis, we used a ~~generalized linear mixed model~~ approach to identify potential significant differences in whistle count changes in the 5 seconds following compared to the 5 seconds before each ping (*pingChange*) for MFAS experiments compared to controls. We used the same fixed effects implemented at the 20-second scale (c*eeType, ceeNum, species, buoyDistance, and groupSize, medWhist)* but also included an autocorrelation structure to this analysis *AR(1)* to account for clear temporal lag effects in exploratory plots.

$$pingChange \sim ceeType + ceeNum + species + groupSize + buoyDist + medWhist + AR(1)$$

Like the 20-second scale, binned ping change data were normally distributed and zero inflated; a Gaussian distribution was used for the conditional model and zero-inflation was modeled as a function of median whistle count (*medWhist)*. We used backward elimination, ΔAIC, and ANOVA to select the final model.

5. CEE-by-CEE analysis

We assessed each playback individually at each time scale to better contextualize the severity, persistence, and directionality (*i.e.*, increase or decrease in whistle count) of responses, which were not measured by either the modeling approach or changepoint analysis. The methods and results for the CEE-by-CEE analysis can be found in the supplementary materials (S2 Appendix).

Ethics Statement

Animal research was conducted under authorization of the United States National Marine Fisheries Service marine mammal research permits 19116 and 19091.

Results

A. Characterization of baseline vocal behavior

1. Basic Description

This analysis includes nine control experiments, each conducted on separate days. Four of these experiments were conducted with *Delphinus delphis* and five were conducted with *Delphinus bairdii* (Table 1). This resulted in 270 minutes of baseline acoustic data for both species (pooled). The average group size across species was 190 individuals (range 45-300 animals). The dispersion of animals varied considerably within and between control experiments, including small to large groups (55-300 individuals) in tight to loose organization, joint (in a single group with no subgroups), or spread out over several subgroups (range: 2-6) at tens to several hundreds of meters apart (range: 10-800 m).

Whistles were successfully detected across all control experimental deployments. Mean (SD) and Median (IQR) whistle count per second varied between control CEEs (Table 1). In our assessment of the control experiments, we found the total number of whistles varied between 0.3-4.6 whistles/second. Note, however, that the inherent uncertainty error in our group size assessment for large groups did not allow us to calculate accurate whistle rates/individual (see table 1 for group size estimates and whistle counts).

CEE ID	species	estimated group size	type	RL (Max)	RL (Range)	whistles per second Median [IQR] (full 30 min period)	# changepoints - mean - pre-exposure	# changepoints - mean - exposure	# changepoints - variance - pre-exposure	# changepoints - variance - exposure
2019_01	Db	260	simMFAS	147 dB re 1μPa	140-147 dB re 1μPa	6.96 [6.39]	26	39	5	1
2019_02	Dd	350	control	n/a	n/a	4.80 [5.19]	37	29	0	5
2019_04	Db	200	control	n/a	n/a	0.25 [0.77]	2	0	4	2
2019_06	Db	45	control	n/a	n/a	0.32 [1.38]	5	0	4	9
2019_07	Db	300	simMFAS	154 dB re 1μPa	150-154 dB re 1μPa	2.88 [3.76]	36	20	3	3
2019_08	Db	250	simMFAS	142 dB re 1μPa	131-142 dB re 1μPa	1.50 [3.91]	30	12	4	4
2019_09	Dd	250	control	n/a	n/a	4.21 [5.38]	46	44	2	4
2019_10	Dd	30	simMFAS	149 dB re 1μPa	146-149 dB re 1μPa	0.091 [0.38]	0	0	3	7
2021_01	Db	150	control	n/a	n/a	3.14 [4.47]	3	51	1	3
2021_02	Db	200	control	n/a	n/a	4.00 [3.78]	18	29	0	4
2021_03	Dd	150	control	n/a	n/a	0.99 [2.01]	11	0	2	0
2021_04	Db	150	control	n/a	n/a	4.61 [4.15]	27	37	4	2
2021_05	Dd	250	control	n/a	n/a	0.36 [1.87]	0	5	8	4
2021_08	Db	30	simMFAS	153 dB re 1μPa	145-153 dB re 1μPa	14.04 [7.75]	50	49	2	4
2021_09	Db	200	simMFAS	157 dB re 1μPa	152-157 dB re 1μPa	1.17 [2.17]	3	6	2	2
2021_10	Db	300	simMFAS	159 dB re 1μPa	150-159 dB re 1μPa	14.62 [9.41]	42	61	3	2
2021_11	Db	10	simMFAS	153 dB re 1μPa	150-153 dB re 1μPa	0.0029 [0.063]	0	0	0	6
2021_12	Dd	150	simMFAS	152 dB re 1μPa	149-152 dB re 1μPa	2.85 [3.07]	7	24	4	6
2021_13	Dd	200	simMFAS	147 dB re 1μPa	139-147 dB re 1μPa	2.21 [4.73]	2	2	9	4

Table [illegible]. Summary of each controlled exposure experiment including controls (no sound emitted) and 'simMFAS' (~~playback of~~ simulated mid-frequency active sonar). CEE-ID contains the year and the CEE number. Species abbreviations are Db for *D. bairdii* and Dd for *D. delphis* and estimated group size is taken from shore observations. Received sound levels (RLs) are reported from Durb[illegible]t al. 2022. Median and interquartile range (IQR, 25-75th percentiles) are given for the raw whistle counts per second of the entire 30-minute CEE. Changepoints were calculated ~~on~~ both the mean whistle count and the variance in whistle count, separately for the

pre-exposure and exposure periods. Note that “exposures” in controls were quiet periods for comparison to sound exposure in MFAS trials.

2. Baseline Changepoint Analysis

The baseline changepoint analysis revealed that mean detected whistle counts over the 30-minute ~~control~~ sampling periods changed once every minute~~,~~ and variance in detected whistle count changed once every 3.5 minutes ~~in~~ control conditions.

B. Assessing the impact of disturbance on whistle behavior

A total of 10 MFAS CEEs were conducted - eight of which included *Delphinus bairdii*, and two of which included *Delphinus delphis*. The calculated average received level across all experiments was 151 dB re 1μPa (range 142-159 dB re 1μPa, Table 1). The average group size for MFAS CEEs was ~173 individuals (range 10-300, Table 1).

1. Number of changepoints in pre-exposure vs exposure

Changepoint analysis was run for all control (9) and MFAS (10) experiments. The number of changepoints detected in both the mean and variance of whistle count during the pre-exposure and exposure periods are presented in Table 1 for both controls and MFAS experiments. There was no significant increase or decrease in the mean and variance of detected whistle counts following MFAS exposure when compared to the natural variance present during control conditions. The difference (Δ) in change points in variance ~~in~~ whistle count between the pre-exposure and exposure period did not differ significantly between controls and MFAS CEEs (controls: $M = 3.1$, $SD = 1.4$; MFAS: $M = 2.5$, $SD = 2.3$; $t(17) = 0.69$, $p = 0.5$). The same was

true when comparing the mean whistle count between both experimental types (controls: M = 11.3, SD = 14.2;MFAS, M = 8.7, SD = 8.5; t(17) = 0.5, p = 0.63).

2. Impact of MFAS exposure on whistle count: 10-minute time scale

Whistle counts did not change between the pre-exposure and exposure period during MFAS experiments. At the 10-minute time scale, the preferred model was the simplest model with the absolute value of the change in median whistle count as a function of only CEE type (either MFAS or control). There was no significant effect of CEE type on the change in median whistle count (negative binomial GLMM, n = 18, p = 0.8). The full model (ΔAIC 5.4) indicated that no proposed predictor variables (CEE type, CEE number, species, buoy distance, or group size) had a significant effect on the change in median whistle count between the pre-exposure and exposure periods for both MFAS and control CEEs (negative binomial GLMM, n = 19: P > 0.05 for all variables, table 2).

Table 2. Overview of GLMMs used at three time-scales - 10 minutes, 20 seconds, and 5 seconds.

conditional model	zero-inflation model	distribution	ΔAIC	degrees of freedom	dispersion (σ^2)
10-minute scale					
abs(wrDiff) ~ ceeType	n/a	nbinom2	0	3	5.44
abs(wrDiff) ~ ceeType + (1 | ceeNum)	n/a	nbinom2	2	4	1.04
abs(wrDiff) ~ ceeType + (1 | ceeNum) + species + buoyDist + groupSize	n/a	nbinom2	5.4	7	1.48
20-second scale					
diff ~ ceeType + expMed	~expMed	gaussian	0	6	7.1
diff ~ ceeType	~expMed	gaussian	5.1	5	7.23
diff ~ ceeType + species + groupSize + buoyDist + expMed	~expMed	gaussian	5.3	9	7.09
diff ~ ceeType + (1 | ceeNum)	~expMed	gaussian	7.1	6	7.23
diff ~ ceeType	none	gaussian	28.3	3	6.71

5-second scale					
diff ~ ceeType + groupSize + expMed + ar1(times + 0 | cee)	~expMed	gaussian	0	9	10.1
diff ~ ceeType + (1 | ceeNum) + species + groupSize + buoyDist + expMed + ar1(times + 0 | cee)	~expMed	gaussian	2.6	12	10.1
diff ~ ceeType + ar1(times + 0 | cee)	~expMed	gaussian	13.8	7	9.83
diff ~ ceeType + (1 | ceeNum) + ar1(times + 0 | cee)	~expMed	gaussian	15.8	8	9.83
diff ~ ceeType	none	gaussian	207.8	3	10.9

3. Characterize the impact of MFAS exposure on whistle count: 20-second time scale

Whistle counts did not differ significantly over the 20-second time windows surrounding each ping. The preferred model at the 20 second scale included only predictor variables for CEE type and median whistle count (table 2). The results of this model showed that ceeType did not have a significant effect on changes in whistle count in the 20-seconds after each ping (GLMM, n=18, slope = 0.47, SE = 0.27, P > 0.05, table 2b, but that the baseline median whistle count for that experimental period was a significant predictor for the change in whistle count following a ping or ghost ping (GLMM, n = 19, slope = 0.76, SE = 0.028, p = 0.0075).

4. Characterize the impact of MFAS exposure on whistle count: 5-second time scale

The preferred model at the 5 second scale included the temporal autocorrelation structure and three explanatory variables, CEE type, group size, and median whistle count, all of which had a significant effect on the change in whistle count in the 5 seconds immediately following a ping compared to the 5 seconds immediately before a ping. When accounting for all other variables, the magnitude of the change in whistle count in the 5 seconds following a true MFAS ping was 1.4x greater than any change in whistle count following ghost pings in control CEEs (GLMM, n=18, slope = 1.43, SE = 0.47, p = 0.002, table 2c). The results of the CEE by CEE analysis (S2

Appendix) showed that in four of the [illegible] experiments in which sound was broadcasted, dolphins exhibited a significant elevation in whistle production in the 5-seconds after each ping throughout the entire 10-minute exposure period, while in three of the [illegible] MFAS exposures, animals showed an initial response to the first ping, with vocal responses slowly abating over the course of the experiment (plots of all raw whistles are provided in S3 Appendix). The effect was particularly pronounced (outside the 75th percentile; Appendix S2 Fig. S2.1) in the first ping of 6 of the MFAS CEEs; whistle activity increased in the 5 seconds following the first MFAS ping up to 15 times the whistle count in the 5 seconds before the first MFAS (mean of all MFAS CEEs 3.9, SD 5.2), compared to increases of only up to 1.4 times (mean 0.46, SD 0.88) at the first ghost ping of controls (Fig. 5, Appendix S2.1). Additionally, group size and median whistle count for the exposure period were found to be significant predictor variables. Larger groups showed larger changes in whistle count following pings and ghost pings (GLMM, n=18, slope = 0.007, SE = 0.003, p = 0.008, table [illegible]and when the median background whistle count was higher, so too were the changes following pings or ghost pings (GLMM, n=18, slope = 0.25, SE = 0.048, p = < 0.005, table 2).

Figure 5. (A) Spectrogram example of 5 seconds before and 5 seconds after the first ping for MFAS CEE 2021_08, illustratin[illegible]e large increase in whistle count immediately following the cessation of the ping. Focal group was comprised of approximately 30 long-beaked common dolphins. The MFAS signal can be seen between 3 and 4 kHz. (B) Boxplot of the change in whistle count from the 5 seconds before to the 5 seconds following each of the 24 pings for CEE 2021_08. Boxplot shows median, 25th, and 75th percentiles, with raw whistle count changes as open gray circles. The change following the first ping is shown as a red star.

Discussion

~~We present a~~ hierarchical approach to quantifying the vocal response of large groups of common dolphins to Navy ~~Sonar and find~~ that the most pronounced acoustic response occurs within the 5-seconds following each ping during MFAS exposure. As in previous observational studies evaluating delphinid acoustic behavior (14), determining a singular behavioral response to MFAS presents several challenges. Multiple factors – including rapid changes in behavioral state over the course of the experiment and variation in group size and composition – make it difficult to ascribe changes in vocal behavior due to disturbance versus natural variability. To better understand typical acoustic variation among common dolphins, we assessed vocal behavior during control conditions and found that they exhibited natural vocal state changes (identified by the changepoint analysis) in whistle production approximately once every minute. This rapid acoustic state switching informed our analytical approach, which utilized a range of temporal windows to test for changes in whistle count (10 minutes - 5 seconds) before and after MFAS exposure. Across the ~~larger~~ time windows selected, we did not detect a change in whistle behavior that was attributable to MFAS. However, CEE type did have a significant effect on the change in whistle count in the 5-seconds immediately following a ping compared to the 5-seconds immediately before a ping.

The initial selection of the time-period over which behavior was assessed in response to Navy sonar (10-minutes) for this project was influenced by multiple factors, including the flight endurance of the drone used for calculating animal distance to our recording buoys (34), our ability to consistently track large groups of fast-moving dolphins, and other previous BRS

~~methods~~ using MFAS (*e.g.,* ~~43, 21~~). Many of the aforementioned constraints are imposed by the logistics of field work. An informed approach to identify behavioral responses to anthropogenic ~~disturbances~~ also requires some prior knowledge of the timing of behavioral state switching in the study species. For example, previous work with blue whales (*Balaenoptera musculus*) evaluated several behavioral metrics (*e.g.,* maximum depth, dive time, ascent/descent rate) in responses to simulated MFAS exposure over a 30-minute time window (43). This exposure duration (which included a 30-minute pre-exposure period) was adequate to capture a behavioral change given the typical duration of their dive cycles (5-8 minutes, 44). In contrast, beaked whales are known to exhibit extremely long, deep foraging dives that often last over an hour followed by long periods of recovery (*e.g.,* 45,46). Consequently, studies focused on direct measurements of behavioral response by Cuvier's beaked whales to MFAS extended their pre-exposure baseline period up to 9.3 hours to ~~perform~~ sound exposure during foraging dives and evaluated their response to sonar for up to 1.7 hours after the exposure period (47).

While the 10-minute exposure period seemed appropriate given the fast-paced lifestyle of common dolphins, neither the aggregate model nor the individual assessment of each CEE detected a change in acoustic behavior that could be ascribed to sonar exposure at this time scale. Even when we explored variation in whistle production during the 20-seconds surrounding each ping, the experimental period was not a significant predictor of changes in whistle count in either the MFAS or control experiments, as vocal state switching often occurs within a 40 second time window under ~~natural~~ conditions. While the impacts of sonar may be evident over the 10-minute exposure period for other behavioral metrics (i.e., changes in behavioral state, group

composition, diving behavior), our analysis reveals that changes in acoustic behavior are limited to an extremely narrow time-window in these species.

It was only at the 5-second time scale surrounding each ping that we observed dolphins exhibiting an acute acoustic response, which often included a rapid increase in whistle production relative to the 5 seconds immediately prior to sound exposure. On average, dolphins increased their whistle count 4 times the average count in the 5 seconds preceding the first ping of the exposure. In one MFAS experiment, dolphins increased their whistle production 15 times compared to the whistle count in the 5 seconds immediately before the first ping (Appendix S2.1). This elevated vocal response following the first ping of the exposure was seen in six of the nine MFAS exposures.

Elevations in whistle counts did not occur during the sonar signal itself – which lasted for 1.6 seconds. Rather, the elevated vocal production occurred once the signal had been transmitted, often abating within ~ 10 seconds. The lack of whistle production occurring during the sonar transmission may be a tactic for reducing acoustic interference and masking which has been shown to impact the detection, discrimination, and localization of relevant signals (48). If the interfering signal is predictable (as is the case in our experiment), then animals should be able to adjust the timing of sound production to limit communication to periods in which noise is reduced (*e.g.*, 48-50). The ability of dolphins to learn the timing of intermittent noise has previously been demonstrated by Finneran et al., 2023 (51) who showed that individuals can modify their hearing sensitivity prior to the onset of predictably timed impulses, presumably to

mitigate ~~negative~~ auditory impacts. Surprisingly, little is known about their capacity to modify the timing of vocal production in response to interfering signals under ~~natural~~ conditions.

The sudden increase in vocal behavior following the first ping could be an example of the amplification of behavior of group members through recruitment or reinforcement (*i.e.,* positive feedback, 52). In this scenario, one dolphin may whistle in response to a surprising, salient stimuli and others follow suit. As this recruitment response continues, the number of dolphins producing whistles increases further and information is spread rapidly throughout the group (53). Alternatively, it could be an indicator that multiple animals are exchanging whistles to contact their closest social partners within the group in the presence of an unknown stimulus. This could be expected given the role of whistles in group cohesion and coordination (2,54) .

Whether animals continued to show an elevated acoustic response immediately following each ping varied between MFAS exposures. In four of the nine experiments in which sound was broadcasted, dolphins exhibited a significant elevation in whistle production in the 5-seconds after each ping throughout the entire 10-minute exposure period (S2 Appendix). This suggests that in these cases, dolphins did not habituate to successive pings (*i.e.*, show progressive decrease in the amplitude of a vocal behavioral response after repeated exposure). However, in three of the nine MFAS exposures, animals showed an initial response to the first ping, with vocal responses slowly abating over the course of the experiment (S2 Appendix). The population of dolphins tested in this study likely live a large portion of their lives in areas that are regularly ensonified by Navy sonar, and thus may exhibit an attenuated response when compared to individuals not regularly exposed to MFAS. Future work could compare the responsiveness of

animals in the Southern California Bight to nearby populations that occur in areas where Navy sonar is not regularly present (*e.g.*, Monterey Bay). Considering whether dolphins may be learning to modify their vocal behavior in response to repeated noise exposure may provide foundational evidence to support using vocal rates as a measure of sensitization or habituation to anthropogenic stimuli, as has been done in terrestrial species (55,56).

Future management decisions mitigating the impact of sonar on oceanic delphinids should consider our reported results of clear responses during CEEs when analyses were conducted at the appropriate temporal resolution . With respect to the most recent methodology for assessing the relative response severity for free-ranging marine mammals to acoustic disturbance (57) – had the acoustic response of animals to MFAS been pooled across the 10-minute time window – common dolphins likely would have been assigned a behavioral response severity score of 0 (no response detected). However, when evaluating vocal behavior across a ~~shorter-time~~ 5-second time window, this species' response would be elevated to a category 3 severity which includes an increase in contact or alarm calls (57). Ultimately, continued work with this (and other closely related) species should also consider how observed behavioral responses vary with respect to other contextual parameters including behavioral state, group composition (*e.g.,* presence or absence of calves), seasonality, and environmental covariates. Concurrent efforts from this project aim to integrate passive acoustics with other remotely sensed datasets (*i.e.*, shore-based group tracking, aerial photogrammetry) to identify group-level behavioral changes and quantify exposure-response relationships (58). Paired with energetic modeling methods (*e.g.,* 31,59,60), these data can be used to link these observed short-term behavioral responses to long-term fitness outcomes in this species and inform the implementation of effective mitigation strategies.

Our study has some limitations that can be addressed in future work. Given the close phylogenetic relationship between short-beaked ~~(*Delphinus delphis*)~~ and long-beaked common dolphins ~~(*Delphinus bairdii*)~~, (61,62), the basic description of baseline vocal behavior (*i.e.,* whistle count data) was combined across species in our analysis. However, recent work by Oswald et al. discovered unique species-specific whistle frequency contours ~~in both *D. delphis* *and D. bairdii*~~ and suggested that these distinctive acoustic signals could help facilitate recognition between these two *Delphinus* species (38). An increase in sample size for both long-beaked and short-beaked common dolphins would provide a more detailed understanding of their baseline vocal behavior and allow for the exploration of whether each species shows a differential or similar response in whistle type usage to MFAS. Additionally, photo-identification of individuals within these large, ephemeral groups is extremely difficult. Consequently, it is unknown whether individual dolphins around Catalina Island were exposed more than once to the experimental treatment. Future research could direct effort towards other oceanic delphinids with smaller group sizes where photo-identification is feasible and reliable (*e.g.,* bo[illegible]nose dolphins).

In any behavioral experiment, the response of the individual or group that is tested should be measured and interpreted over a time-window that is informed by their natural behavior. We suggest that future work with other oceanic delphinid species explore baseline vocal rates a-priori and use information on vocal state-switching to inform the analysis time-window over which behavioral responses are measured. Given these species' susceptibility to frequent MFAS exposure in Navy operational areas, it is of particular interest to evaluate how repeated exposure

influences responses. Future work should continue to explore key factors that are likely to influence the probability of response among these large groups, including their behavioral state and their proximity to the sound source and received level at the onset of exposure.

Acknowledgements

Funding for this project was provided by the U.S. Navy's Office of Naval Research (Award Numbers N000141713132, N0001418IP-00021, N000141712887, N000141912572). Animal observations, octocopter flights over dolphins, close approaches and CEEs were conducted under NMFS permit 19116 and 19091. We thank the M/V Magician and Captain Carl Mayhugh for support in the field, as well as the University of Southern California's Wrigley Institute for their hospitality throughout the duration of this project. This project would not have been possible without the dedicated contribution of all members of the Tagless-BRS team. Thank you to Stacy DeRuiter for extensive advice on statistical modeling and analysis. Additionally, we appreciate the thoughtful feedback provided by Dr. Ari Friedlaender, Dr. Colleen Reichmuth, Dr. Peter Cook, and Dr. John Durban during analysis and manuscript preparation. We also thank Ryan Jones, who created the line drawings for Fig. 1.

References

1. Tyack PL. Acoustic Communication Under the Sea. In: Hopp SL, Owren MJ, Evans CS, editors. Animal Acoustic Communication. Springer Berlin / Heidelberg; 1998.

2. Janik VM. Acoustic Communication in Delphinids. Adv Study Behav. 2009 ;40:123–157.

3. Lammers MO, Oswald JN. Analyzing the acoustic communication of dolphins. In: Herzing DL, Johnson CM, editors. Dolphin communication and cognition: Past, present, and future. MIT Press; 2015. pp. 107-130.

4. Erbe C, Dunlop R, Dolman S. Effects of Noise on Marine Mammals. In: Slabbekoorn H, Dooling R, Popper A, Fay R, editors. Effects of Anthropogenic Noise on Animals. New York : Springer; 2018.

5. Williams R, Wright AJ, Ashe E, Blight LK, Bruintjes R, Canessa R, et al. Impacts of anthropogenic noise on marine life: Publication patterns, new discoveries, and future directions in research and management. Ocean Coast Manag. 2015;115:17–24.

6. Southall BL, Finneran JJ, Reichmuth C, Nachtigall PE, Ketten DR, Bowles AE, et al. Marine mammal noise exposure criteria: Updated scientific recommendations for residual hearing effects. Aquat Mamm. 2019;45(2):125–232.

7. Hatch LT, Wahle CM, Gedamke Jason, Harrison Jolie, Laws Benjamin, Moore SE, et al. Can you hear me here? Managing acoustic habitat in US waters. Endanger Species Res. 2016;30(1):171–86.

8. Tyack PL, Janik VM. Effects of Noise on Acoustic Signal Production in Marine Mammals. In: Brumm H, editor. Animal Communication and Noise Animal Signals and Communication. Springer; 2013.pp. 251-271.

9. Nowacek DP, Thorne LH, Johnston DW, Tyack PL. Responses of cetaceans to anthropogenic noise. Mamm Rev. 2007;37(2):81–115.

10. Hastie G, Merchant ND, Götz T, Russell DJF, Thompson P, Janik VM. Effects of impulsive noise on marine mammals: investigating range-dependent risk. Ecol Appl.. 2019;29:e01906.

11. Harris CM, Sadykova D, DeRuiter SL, Tyack PL, Miller PJO, Kvadsheim PH, et al. Dose response severity functions for acoustic disturbance in cetaceans using recurrent event survival analysis. Ecosphere. 2015;6(11):1–14.

12. Southall EBL, Finneran JJ, Reichmuth C, Nachtigall PE, Ketten DR, Bowles AE, et al. Marine mammal noise exposure criteria: Updated scientific recommendations for residual hearing effects. Aquat Mamm. 2019;45(2):125–232.

13. D'Amico A, Pittenger R. A brief history of active sonar. Aquat Mamm. 2009 Dec;35(4):426–34.

14. Henderson EE, Smith MH, Gassmann M, Wiggins SM, Douglas AB, Hildebrand JA. Delphinid behavioral responses to incidental mid-frequency active sonar. J Acoust Soc Am. 2014;136(4):2003–14. Available from: http://dx.doi.org/10.1121/1.4895681

15. Richardson WJ, Thomson DH, Greene CR, Malme CI. Marine mammal sounds. In: Marine Mammals and Noise. San Diego, CA: Academic Press; 1995.

16. Oswald JN, Rankin S, Barlow J. The effect of recording and analysis bandwidth on acoustic identification of delphinid species. J Acoust Soc Am. 2004 Nov 1;116(5):3178–85.

17. Filadelfo R, Mintz J, Michlovich E, D'Amico A, Tyack PL, Ketten DR. Correlating military sonar use with beaked whale mass strandings: What do the historical data show? Aquat Mamm. 2009;35(4):435–44.

18. D'Amico A, Gisiner RC, Ketten DR, Hammock JA, Johnson C, Tyack PL, et al. Beaked whale strandings and naval exercises. Aquat Mamm. 2009;35(4):452–72.

19. Guan S, Brookens T. An overview of research efforts to understand the effects of underwater sound on cetaceans. Vol. 2, Water Biology and Security. Institute of Hydrobiology, Chinese Academy of Sciences; 2023; 2:100141.

20. Ioannou CC, Laskowski KL. A multi-scale review of the dynamics of collective behaviour: from rapid responses to ontogeny and evolution. Philosophical Transactions of the Royal Society B. 202310;378(1874):20220059.Available from: https://doi.org/10.1098/rstb.2022.0059

21. Southall BL, DeRuiter SL, Friedlaender A, Stimpert AK, Goldbogen JA, Hazen E, et al. Behavioral responses of individual blue whales (*Balaenoptera musculus*) to mid-frequency military sonar. Journal of Experimental Biology. 2019;222(5).

22. Sivle LD, Kvadsheim PH, Fahlman A, Lam FPA, Tyack PL, Miller PJO, et al. Changes in dive behavior during naval sonar exposure in killer whales, long-finned pilot whales, and sperm whales. Frontiers in Physiology. 2012; 11;3:400. Available from: www.frontiersin.org

23. Deruiter SL, Boyd IL, Claridge DE, Clark CW, Gagnon C, Southall BL, et al. Delphinid whistle production and call matching during playback of simulated military sonar. Mar Mamm Sci. 2013;29(2).

24. Tyack PL, Zimmer WMX, Moretti D, Southall BL, Claridge DE, Durban JW, et al. Beaked whales respond to simulated and actual navy sonar. PLoS One. 2011;6(3).

25. Durbach IN, Harris CM, Martin C, Helble TA, Henderson EE, Ierley G, et al. Changes in the Movement and Calling Behavior of Minke Whales (*Balaenoptera acutorostrata*) in Response to Navy Training. Front Mar Sci. 2021;8.

26. Rendell LE, Gordon JCD. Vocal response of long-finned pilot whales (*Globicephala melas*) to military sonar in the Ligurian Sea. Mar Mamm Sci. 1999;15(1):198–204.

27. Miller PJO, Antunes RN, Wensveen PJ, Samarra FIP, Catarina Alves A, Tyack PL, et al. Dose-response relationships for the onset of avoidance of sonar by free-ranging killer whales. J Acoust Soc Am. 2014;135(2):975–93. Available from: http://asa.scitation.org/doi/10.1121/1.4861346

28. DeRuiter SL, Boyd IL, Claridge DE, Clark CW, Gagnon C, Southall BL, Tyack PL. Delphinid whistle production and call matching during playback of simulated military sonar. Marine Mammal Science. 2013 Apr;29(2):E46-59.Available from: https://onlinelibrary.wiley.com/doi/10.1111/j.1748-7692.2012.00587.x

30. Lara G, Bou-Cabo M, Llorens S, Miralles R, Espinosa V. Acoustical Behavior of Delphinid Whistles in the Presence of an Underwater Explosion Event in the Mediterranean Coastal

Waters of Spain. Journal of Marine Science and Engineering. 2023;11(4):780. Available from: https://doi.org/10.3390/jmse11040780

31. Keen KA, Beltran RS, Pirotta E, Costa DP. Emerging themes in population consequences of disturbance models. Proceedings of the Royal Society B. 2021;288(1957):20210325.

32. King SL, Connor RC, Montgomery SH. Social and vocal complexity in bottlenose dolphins. Vol. 45, Trends in Neurosciences. Elsevier Ltd; 2022. p. 881–3.

33. Cunha HA, Loizaga De Castro R, Secchi ER, Crespo EA, Lailson-Brito J, Azevedo AF, et al. Molecular and morphological differentiation of common dolphins (*Delphinus* sp.) in the Southwestern Atlantic: testing the two species hypothesis in sympatry. PloS one. 2015 Nov 11;10(11):e0140251.

34. Durban JW, Southall BL, Calambokidis J, Casey C, Fearnbach H, Joyce TW, et al. Integrating remote sensing methods during controlled exposure experiments to quantify group responses of dolphins to navy sonar. Mar Pollut Bull. 2022;174:113194. Available from: https://doi.org/10.1016/j.marpolbul.2021.113194

35. Southall BL, Moretti D, Abraham B, Calambokidis J, Deruiter SL, Tyack PL. Marine mammal behavioral response studies in southern California: advances in technology and experimental methods. Marine Technology Society Journal. 2012 Jul 1;46(4):48-59

36. Visser, F. (2014). Moving in concert: Social and migratory behaviour of dolphins and whalesin the North Atlantic Ocean. PhD dissertation. University of Amsterdam. 2014.

37. Hager CA. Assessment of the performance of the near-bottom hydrophones of the US Navy Southern California offshore range in detecting, localizing and reconstructing 10-20KHZ odontocete whistles. PhD dissertation. Naval Postgraduate School. 2008.

38. Oswald JN, Walmsley SF, Casey C, Fregosi S, Southall B, Janik VM. Species information in whistle frequency modulation patterns of common dolphins. Phil TransR Soc B. 2021;376:20210046.

39. Gillespie D, Mellinger DK, Gordon J, David McLaren, Redmond P, McHugh R, et al. PAMGUARD: Semiautomated, open source software for real- time acoustic detection and localization of cetaceans. J Acoust Soc Am. 2009;125(4):2547–2547.

40. Sakai T et al. R Package 'PAMpal'. https://doi.org/10.1121/1.2743157;2022.

41. Killick R, Fearnhead P, Eckley IA. Killick R, Fearnhead P, Eckley IA. Optimal detection of changepoints with a linear computational cost. Journal of the American Statistical Association. 2012 Dec 1;107(500):1590-8.

42. Brooks ME;, Kristensen K;, Van Benthem KJ;, Magnusson A;, Berg CW;, Nielsen A;, et al. glmmTMB balances speed and flexibility among packages for zero-inflated generalized linear mixed modeling. The R journal. 2017;9(2):378-400. Available from: https://doi.org/10.3929/ethz-b-000240890

43. Goldbogen JA, Southall BL, DeRuiter SL, Calambokidis J, Friedlaender AS, Haz EL, et al. Blue whales respond to simulated mid-frequency military sonar. Proceeding of the Royal Society B. 2013;280:20130657.

44. Croll DA, Acevedo-Gutierrez A, Tershy BR, Urban-Ramırez J, Croll D. The diving behavior of blue and fin whales: is dive duration shorter than expected based on oxygen stores? Comparative Biochemistry and Physiology Part A: Molecular & Integrative Physiology. 2001 Jul 1;129(4):797-809.

45. Baird RW, Webster DL, Schorr GS, McSweeney DJ, Barlow J. Diel variation in beaked whale diving behavior. Mar Mamm Sci. 2008 Jul;24(3):630–42.

46. Shearer JM, Quick NJ, Cioffi WR, Baird RW, Webster DL, Foley HJ, et al. Diving behaviour of Cuvier's beaked whales (Ziphius cavirostris) off Cape Hatteras, North Carolina. R Soc Open Sci. 2019 Feb 1;6(2).

47. Deruiter SL, Southall BL, Calambokidis J, Zimmer WMX, Sadykova D, Falcone EA, et al. First direct measurements of behavioural responses by Cuvier ' s beaked whales to mid-frequency active sonar. 2013;9:20130223.

48. Egnor SR, Wickelgren JG, Hauser MD. Tracking silence: adjusting vocal production to avoid acoustic interference. Journal of Comparative Physiology A. 2007 Apr;193:477-83.

49. Kiai A, Clemens J, Kössl M, Poeppel D, Hechavarría JC. Flexible control of vocal timing in bats enables escape from acoustic interference. bioRxiv. 2023:2023-05.

50. Munoz-Santos I, Ríos-Chelén AA. Vermilion flycatchers avoid singing during sudden peaks of anthropogenic noise. acta ethologica. 2022 Dec 26:1-0.

51. Finneran JJ, Schlundt CE, Bowman V, Jenkins K. Dolphins reduce hearing sensitivity in anticipation of repetitive impulsive noise exposures. The Journal of the Acoustical Society of America. 2023 Jun 1;153(6):3372.

52. Bonabeau E, Theraulaz G, Deneubourg J.L, Aron S, Camazine S. Self-organization in social insects. Trends Ecol Evol. 1997;12(188).

53. Sumpter DJT. The principles of collective animal behaviour. Philosophical Transactions of the Royal Society B. 2005;361:5–22. Available from: https://royalsocietypublishing.org/

54. Caldwell MC, Caldwell DK. Vocalization of naive captive dolphins in small groups. Science. 1968 Mar 8;159(3819):1121-3.

55. Francis CD, Ortega CP, Cruz A. Noise Pollution Changes Avian Communities and Species Interactions. Current Biology. 2009 Aug 25;19(16):1415–9.

56. Brown CL, Hardy AR, Barber JR, Fristrup KM, Crooks KR, Angeloni LM. Brown CL, Hardy AR, Barber JR, Fristrup KM, Crooks KR, Angeloni LM. The effect of human activities and their associated noise on ungulate behavior. PloS one. 2012 Jul 10;7(7):e40505.

57. Southall BL, Tollit D, Amaral J, Clark CW, Ellison WT. Managing human activity and marine mammals: A biologically based, relativistic risk assessment framework. Front Mar Sci. 2023;10.

58. Harris CM, Thomas L, Falcone EA, Hildebrand J, Houser D, et al. Marine mammals and sonar: Dose- response studies, the risk- disturbance hypothesis and the role of exposure

context. J Appl Ecol. 2018;55:396-404. Available from: https://besjournals.onlinelibrary.wiley.com/doi/10.1111/1365-2664.12955

59. Harwood J, King S, Booth C, Donovan C, Schick RS, Thomas L, et al. Understanding the population consequences of acoustic disturbance for marine mammals. InThe effects of noise on aquatic life II 2016 (pp. 417-423). Springer New York.

60. McHuron EA, Adamczak S, Costa DP, Booth C. Estimating reproductive costs in marine mammal bioenergetic models: a review of current knowledge and data availability. Conservation Physiology. 2023;11(1):coac080.

61. Natoli A, Cañadas A, Peddemors VM, Aguilar A, Vaquero C, Fernandez- Piqueras P, Hoelzel AR. Phylogeography and alpha taxonomy of the common dolphin (*Delphinus* sp.). Journal of Evolutionary Biology. 2006 May;19(3):943-54.

62. ~~Mcgowen~~ MR, Tsagkogeorga G, Álvarez-Carretero S, Reis M Dos, Struebig M, Deaville R, et al. Phylogenomic resolution of the cetacean tree of life using target sequence capture. Systematic biology. 2020 May 1;69(3):479-501.

Author contributions

BLS acquired the funding for this project, and BLS, CC, and SF conceived, designed, and performed the experiment. CC and SF analyzed the data, while BLS, VMJ, and JNO provided analytical guidance. CC, SF, VMJ, JNO, and BLS wrote the manuscript.

Figure 1

Click here to access/download;Figure;Fig. 1.tif

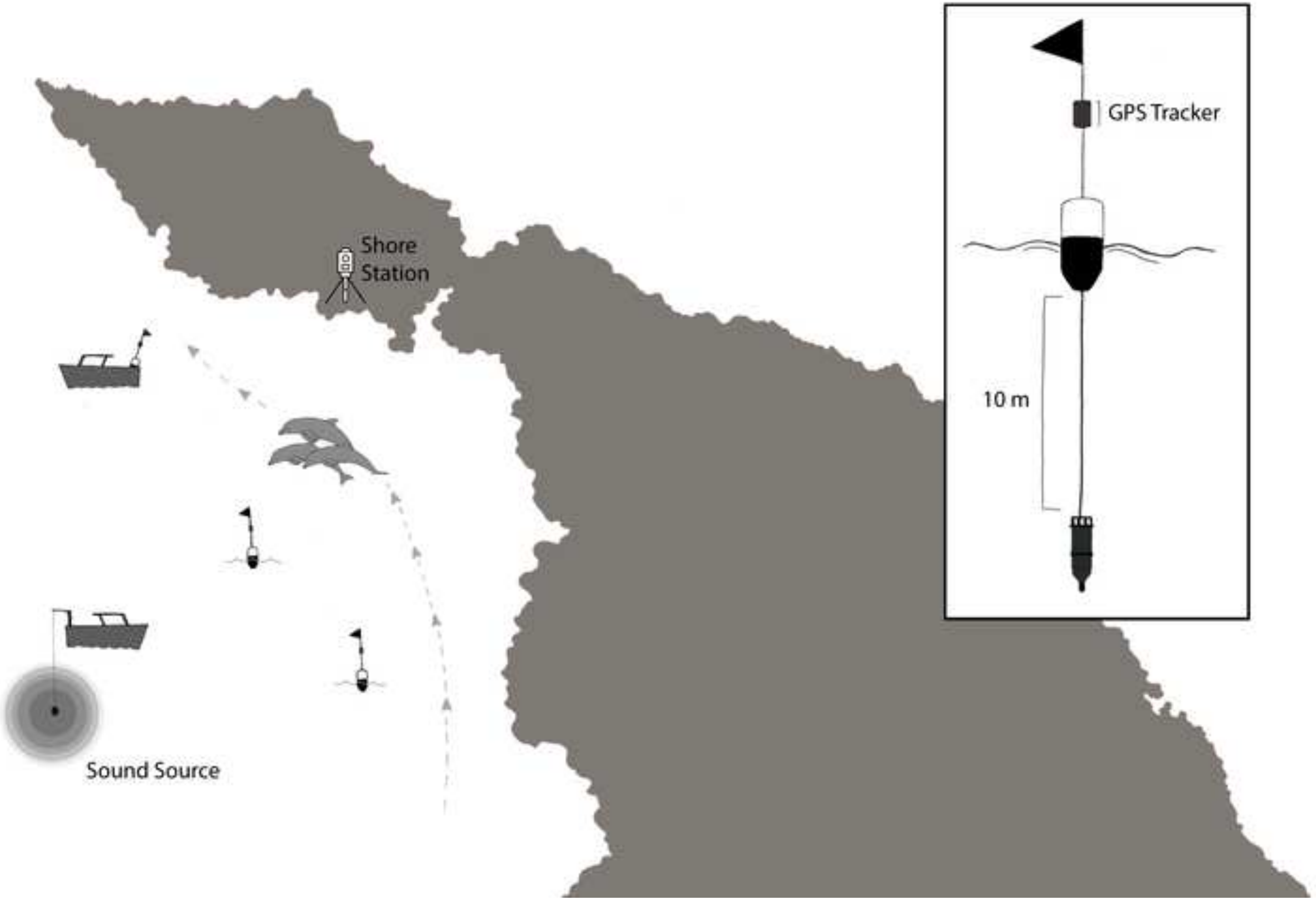

Figure 2

Click here to access/download;Figure;Fig. 2.tif

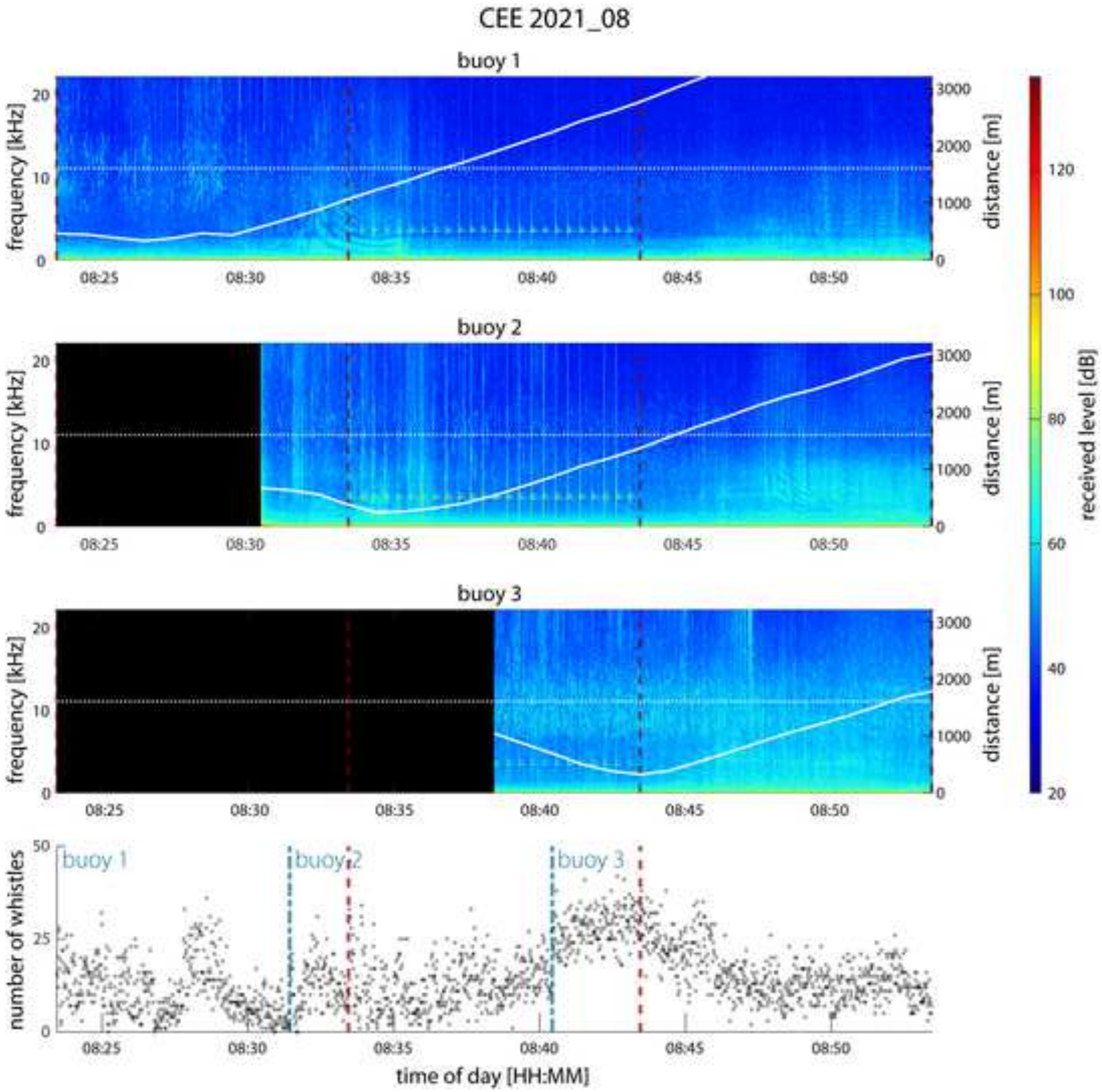

Figure 3

Click here to access/download;Figure;Fig 3.tif

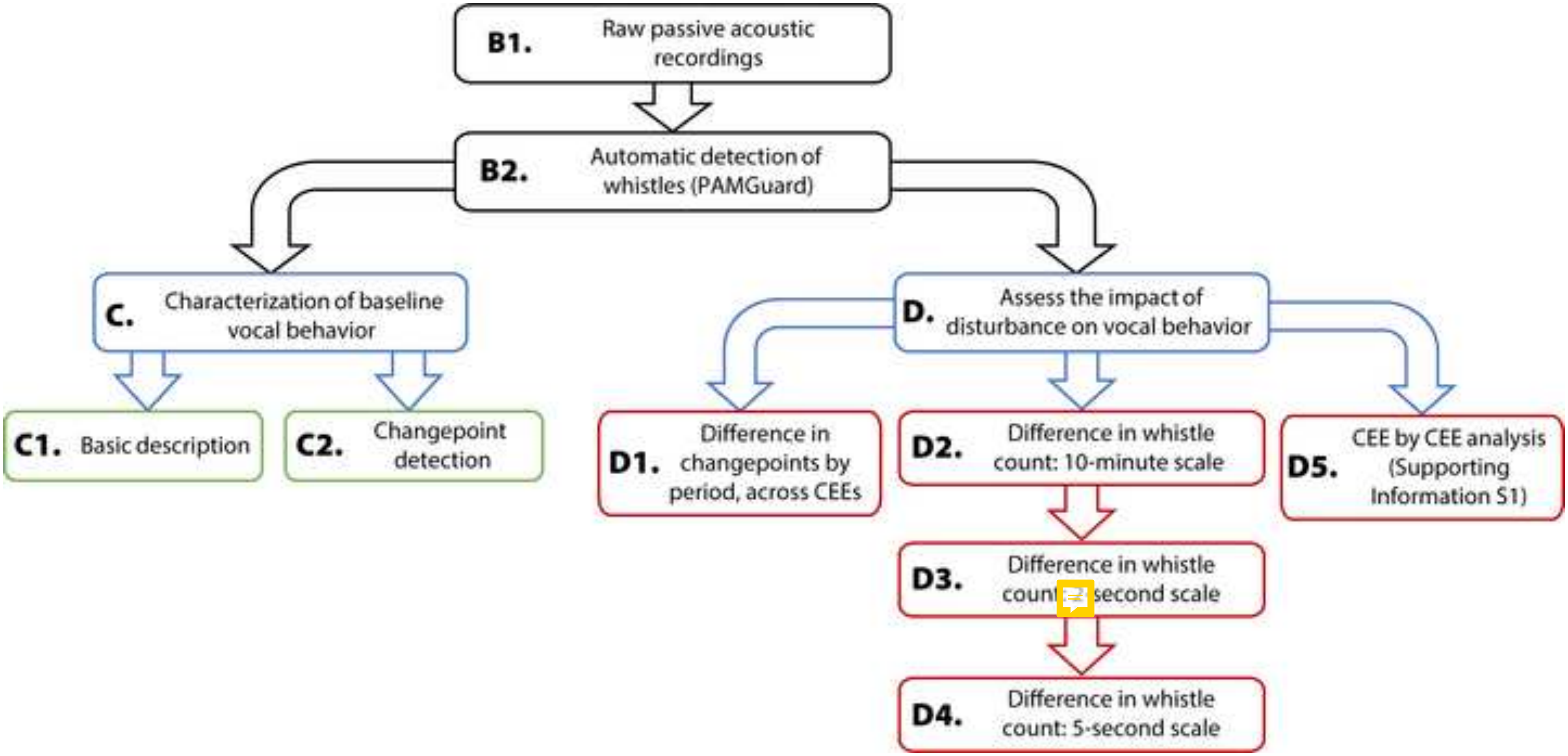

Figure 4

Click here to access/download;Figure;Fig. 4.tif

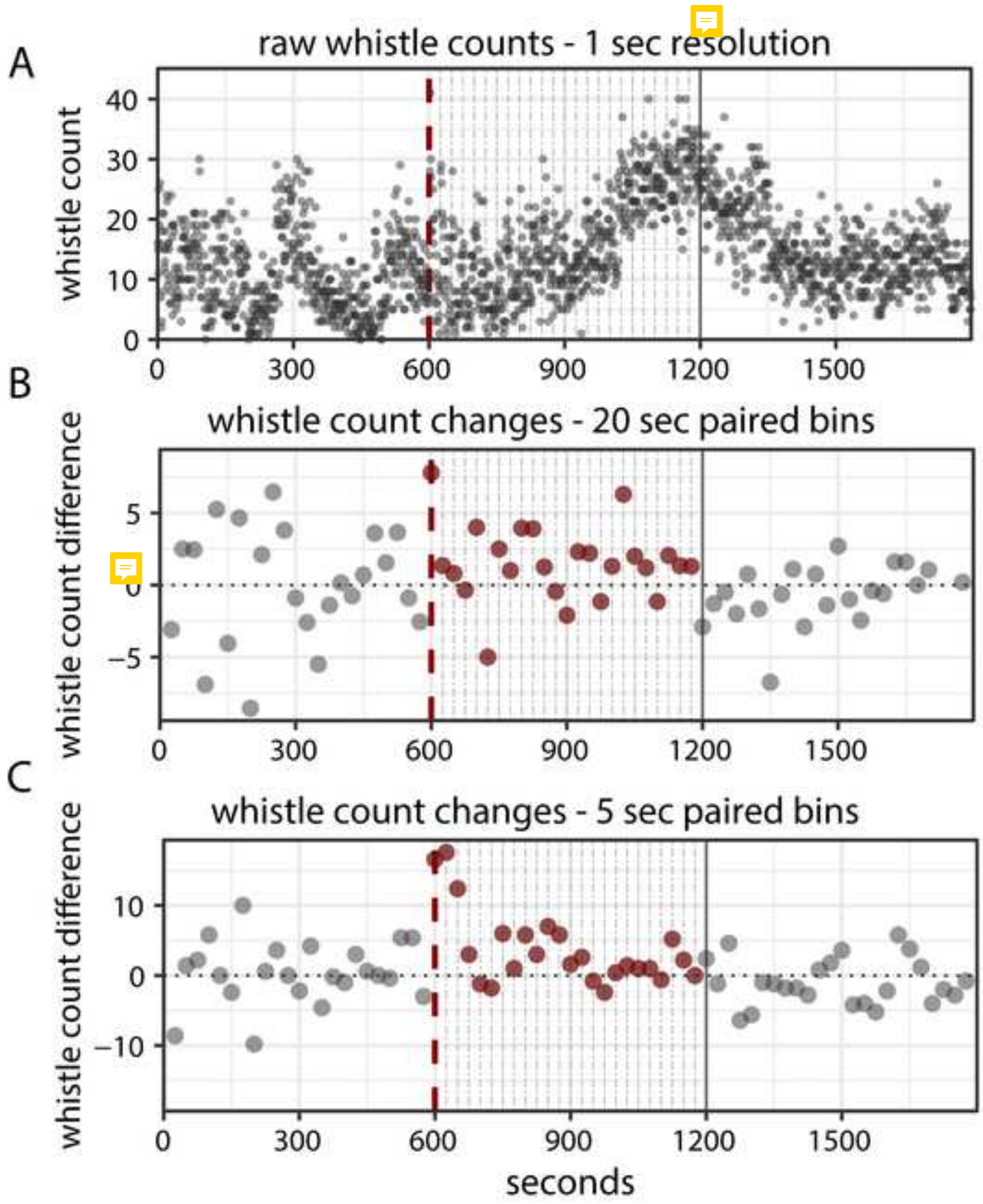

Figure 5

Click here to access/download;Figure;Fig 5.tif

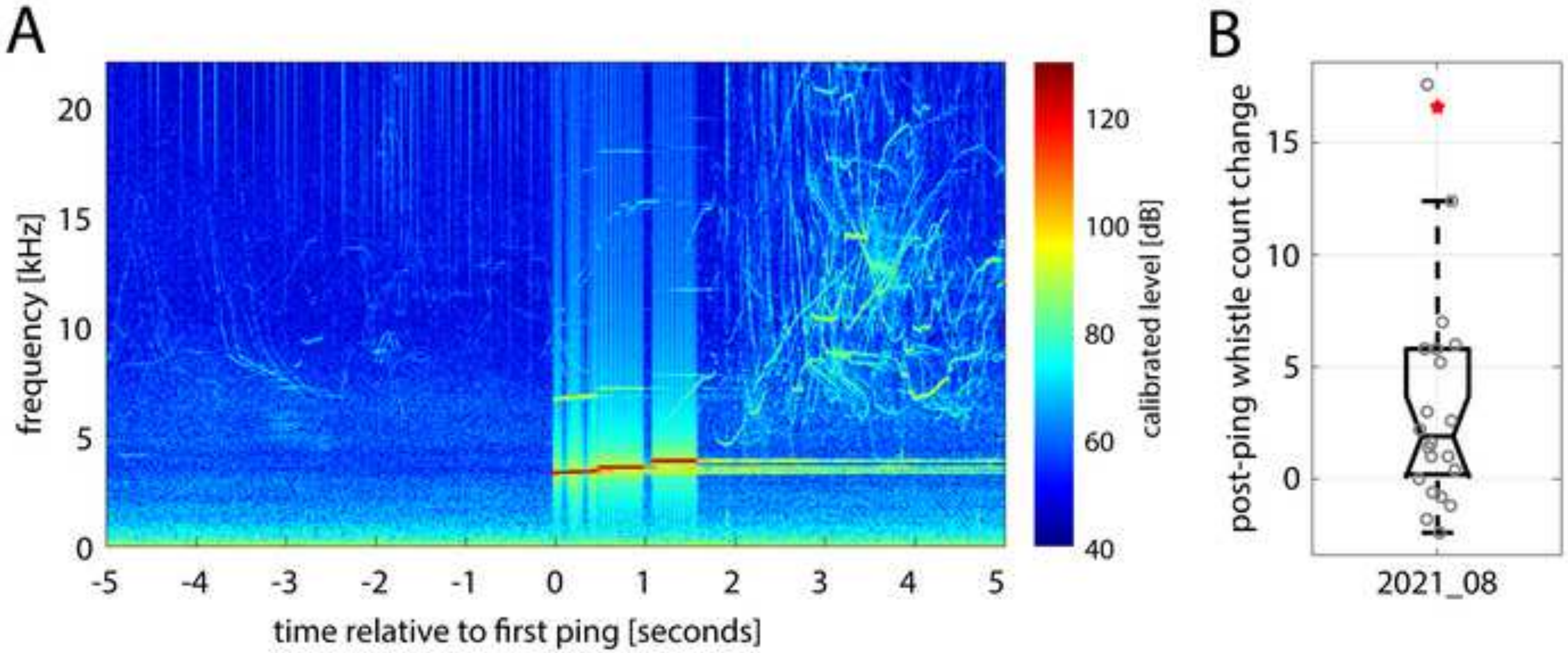

Click here to access/download
Supporting Information
Casey_et al supporting information.docx
