# Supplementary material for: Common dolphin whistle responses to experimental mid-frequency sonar
Source: PLoS One. 2024 Apr 26;19(4):e0302035. doi: 10.1371/journal.pone.0302035 (PMC11051594; doi:10.1371/journal.pone.0302035)
Supplement: Attachment — Submitted filename: Casey_et al supporting information comments.pdf [file pone.0302035.s006.pdf]

Common dolphin acoustic response to experimental mid-frequency sonar

*Caroline Casey[a,b], *Selene Fregosi[a], Julie N. Oswald[c], Vincent M. Janik[c], Fleur Visser[d,e], Brandon Southall[a,b]

[a]Southall Environmental Associates, Inc., 9099 Soquel Drive, Aptos, CA 95003, USA
[b]Institute of Marine Sciences, University of California Santa Cruz, 115 McAllister Way, Santa Cruz, CA 95060, USA
[c]Sea Mammal Research Unit, Scottish Oceans Institute, School of Biology, University of St. Andrews, St. Andrews, KY16 8LB, United Kingdom
[d]Kelp Marine Research, 1624 CJ, Hoorn, the Netherlands
[e]Department of Coastal Systems, Royal Netherlands Institute for Sea Research, P.O. Box 59, 1790 AB Den Burg, Texel, the Netherlands

Corresponding author: Caroline Casey, cbcasey@ucsc.edu

S1 Appendix: PAMGuard Whistle Detection Parameters

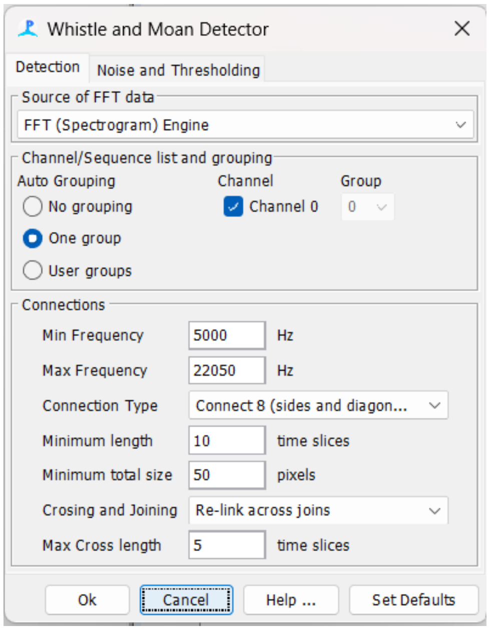

Figure S1.1. Detection settings for the PAMGuard Whistle and Moan Detector

S2 Appendix: CEE-by-CEE Analysis

The main manuscript presents methods and results for analyzing common dolphin vocal response to simulated mid-frequency sonar, pooling data across all CEEs. We acknowledge that some readers may be interested in additional information about each of the 19 CEEs (10 MFAS exposures and 9 controls), and so we have included additional methods and results for each individual CEE below.

Methods

In the CEE-by-CEE analysis, potential differences between controls vs MFAS CEEs were not compared, but rather comparisons were made within individual CEEs, between the pre-exposure vs the exposure period. Like the pooled response analysis, the CEE-by-CEE analysis was conducted at three scales using three different analysis window sizes: 10 minutes (the entire pre-exposure period compared to the entire exposure period), 20 seconds (changes between pairs of 20-sec duration bins), and 5 seconds (changes between pairs of 5-sec duration bins).

A. Differences in pre-exposure vs exposure period whistle counts: 10-minute time scale

Similar to the aggregate model approach presented in the main manuscript, we used a Generalized Linear Mixed Model (GLMM) approach. We evaluated raw whistle counts (per second), *count*, as a function of experimental period (either pre or exposure), *period*, and the distance from the closest buoy to the focal group (interpolated every second), *buoyDistance*, by fitting a negative binomial model using R package `glmmTMB`. This is a suitable approach for count data that are overdispersed relative to a Poisson distribution (1). Of the two negative binomial fits available, the fit with the lowest AIC was chosen on a CEE-by-CEE basis. To account for temporal autocorrelation in this time series dataset, we also included a covariance structure (*ar1(time)*) which improved model performance.

$$count \sim period + buoyDistance + ar1(time) \quad \text{(S1)}$$

One of the 10 MFAS experiments had to be excluded from this analysis (and all remaining within CEE analyses) due to an overall low (near zero) rate of whistle production throughout the

experiment (CEE 2021_11). Plots of all raw per-second whistle counts for each CEE are shown below in Supporting Appendix S3.

B. Differences in whistle counts in sequential 20 second time bin pairs

To characterize changes in whistle production over a shorter time window, we compared whistle counts 20 seconds before and 20 seconds after each individual ping (n = 24 1-second pings total over a 10-minute experimental period, ~25 seconds between each ping) and then used linear regression to model the magnitude of these changes between bin pairs as a function of experimental period. We selected this time window to capture sustained variation in whistling behavior within a single ping cycle, without overlap between cycles.

We first calculated the mean number of whistles per second (whistle count) in the 20 seconds before and 20 seconds after each ping. Differences between these two subsequent time bins were calculated by subtracting the mean whistle count for the first bin from the mean whistle count of the second bin. The first ping started at time 0, the second ping at time 25 seconds, and so on. We repeated this process for the pre-exposure period to enable assessment of potential impacts of the MFAS playback signal. Because no actual pings were present in the pre-exposure period, the pairs of bins were centered around the analogous time to when pings occurred in the exposure period (i.e., 25 sec, 50 sec, 75 sec, etc for the full 10-minute pre-exposure period). To enable a post-hoc comparison across experiment types (control vs simulated MFAS exposure) we applied the same approach to controls using the times at which pings would have occurred (ghost pings) as the midpoint around which the bins were placed.

These paired bin differences served as the response variable, *binDiffs*, and the explanatory variable was a categorical variable *period*, either pre-exposure or exposure. We observed heteroscedasticity in the raw data; whistle variance varied with average whistle count. We thus applied a variance structure that allowed variance in whistle count to vary exponentially with average whistle count using the R package `nlme`.

$$binDiffs \sim period + \in \qquad \textit{(S2)}$$

Each CEE (both controls and MFAS exposures) was modeled individually. We then assessed how often the CEE period significantly predicted differences in whistle count for both the controls and CEEs.

C. Differences in whistle counts in subsequent 5 second time bin pairs

The same analysis and linear regression modelling approach for 20-second duration subsequent bins was applied but using pairs of sequential bins of only 5 seconds duration. During the exposure period these bins were centered around the pings. During the pre-exposure period and the exposure period for controls, these bins were centered around the time in which pings would have occurred. Again, each CEE was modeled individually and ad-hoc comparisons were made across CEEs.

Results

A. Differences in whistle count at the 10-minute scale

Of the 18 CEEs that had whistles present enough to be modeled, 7 had a significant difference in whistle count between the pre-exposure and exposure period – 5 of the 7 significant differences were MFAS exposures and 2 were controls. Of the 9 MFAS CEEs, 5 showed a significant difference in whistle count between CEE experimental periods and 4 did not. Those with significant differences varied in the level and direction of response.

Commented [Anon.1]: Why weren't the pre- and post-exposure periods compared as well?

Commented [Anon.2]: Does this mean number and frequency (more/less) of whistles?

When I see level and direction in a manuscript about BRSs, I immediately think sound level/amplitude and direction of the response (away/toward) or direction of whistle. Please clarify what was intended.

Table S2.1 Results of GLMM analysis of whistle count as a function of experimental period and buoy distance, for each CEE individually.

CEE	CEE Type	*period* estimate	*period* pvalue	*buoyDistance* estimate	*buoyDistance* pvalue
2019_01	MFAS	1.0978	0.0000108*	0.000496	0.75982
2019_02	Control	0.3171	0.6329465	-0.0029648	0.0369*
2019_04	Control	-0.4365	0.4713107	-0.0012041	0.40453
2019_06	Control	-1.27	0.2326034	-0.0027367	0.1435
2019_07	MFAS	0.826	0.1955801	-0.0017367	0.01269*
2019_08	MFAS	-0.4248	0.3157593	-0.0012726	0.29314
2019_09	Control	0.3337	0.4489849	-0.0016191	0.08367

2019_10	MFAS	1.0946	0.6294804	-0.0001284	0.96733
2021_01	Control	0.8132	0.359100	-0.00397	0.029110*
2021_02	Control	0.5136	0.026200*	-0.0005	0.283900
2021_03	Control	-0.5739	0.026200*	0.000776	0.489900
2021_04	Control	0.2873	0.232400	-0.00032	0.523500
2021_05	Control	2.0624	0.032640	0.004451	0.107800
2021_08	MFAS	0.4533	0.007578*	0.00057	0.408000
2021_09	MFAS	1.505	0.005311*	-0.0019	0.329500
2021_10	MFAS	-0.2053	0.010980*	-0.00041	0.171900
2021_12	MFAS	0.4103	0.404600	-0.00018	0.889200
2021_13	MFAS	-1.7524	0.000001*	-0.00396	0.000099*

*significance at the p < 0.05 level

B. Differences in whistle count at the 20-second scale

No CEEs showed a significant relationship in the change in whistle count between two sequential bins of 20 second duration and the experimental period.

Table S2.2 Results of linear regression analysis of the short-duration difference in whistle count between sequential 5 second paired bins, as a function of experimental period, for each CEE individually.

CEE	CEE Type	*period* estimate	*period* pvalue
2019_01	MFAS	1.01689	0.1810
2019_02	Control	0.25667	0.6388
2019_04	Control	-0.01827	0.6181
2019_06	Control	0.04426	0.3751
2019_07	MFAS	0.20424	0.7420
2019_08	MFAS	0.03803	0.9040
2019_09	Control	0.67586	0.4569
2019_10	MFAS	-0.01052	0.3234

2021_01	Control	0.31829	0.5319
2021_02	Control	0.20341	0.7258
2021_03	Control	0.03467	0.8109
2021_04	Control	-0.34744	0.5421
2021_05	Control	-0.01071	0.6308
2021_08	MFAS	1.52019	0.0944
2021_09	MFAS	0.09414	0.5128
2021_10	MFAS	0.58794	0.6578
2021_12	MFAS	-0.08095	0.8409
2021_13	MFAS	-0.01812	0.7481

*significance at the p < 0.05 level

C. Differences in whistle count at the 5-second scale

There was a significant effect of period on the differences in whistle count between two sequential 5 second bins in 5 of 18 CEEs; all 5 significant CEEs were MFAS exposure CEEs. All 9 controls were non-significant. The effect in all 5 significant MFAS CEEs was positive, indicating an increase in whistle count in the second of the 5 second bins. Effect sizes ranged from 0.5 to 5.7 times greater whistle count in the second bin in bin pairs during the exposure period compared to the pre-exposure period.

Table S2.3 Results of linear regression analysis of the short-duration difference in whistle count between sequential 5 second paired bins, as a function of experimental period, for each CEE individually.

CEE	CEE Type	*period* estimate	*period* pvalue
2019_01	MFAS	3.2420	0.000012*
2019_02	Control	0.5070	0.4839
2019_04	Control	0.0225	0.6345
2019_06	Control	-0.0013	0.9774
2019_07	MFAS	1.6920	0.005099*

2019_08	MFAS	0.3218	0.3899
2019_09	Control	1.4060	0.07018
2019_10	MFAS	0.0000	1.0000
2021_01	Control	0.070529	0.8475
2021_02	Control	0.193609	0.7284
2021_03	Control	-0.13344	0.4807
2021_04	Control	-0.56681	0.3358
2021_05	Control	0.001388	0.8491
2021_08	MFAS	2.947619	0.046380*
2021_09	MFAS	0.508054	0.049320*
2021_10	MFAS	5.727571	0.000001*
2021_12	MFAS	0.09499	0.8309
2021_13	MFAS	-0.03488	0.5048

*significance at the $p < 0.05$ level

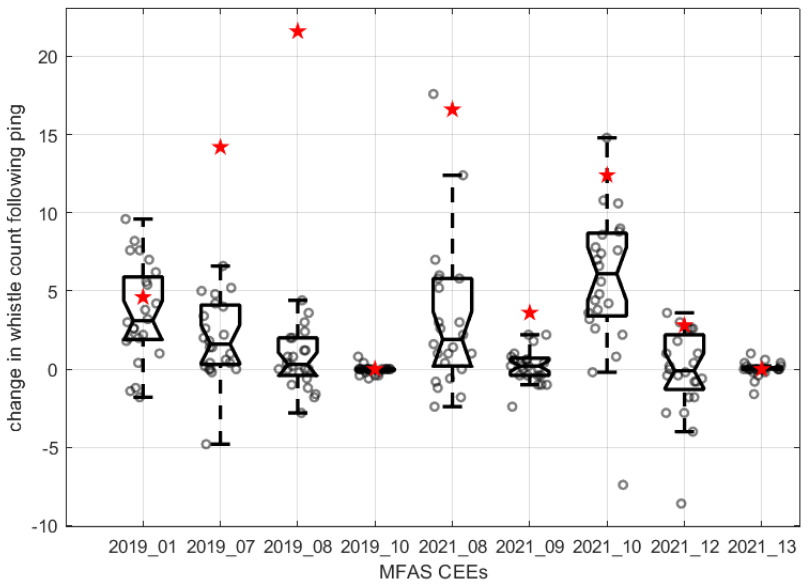

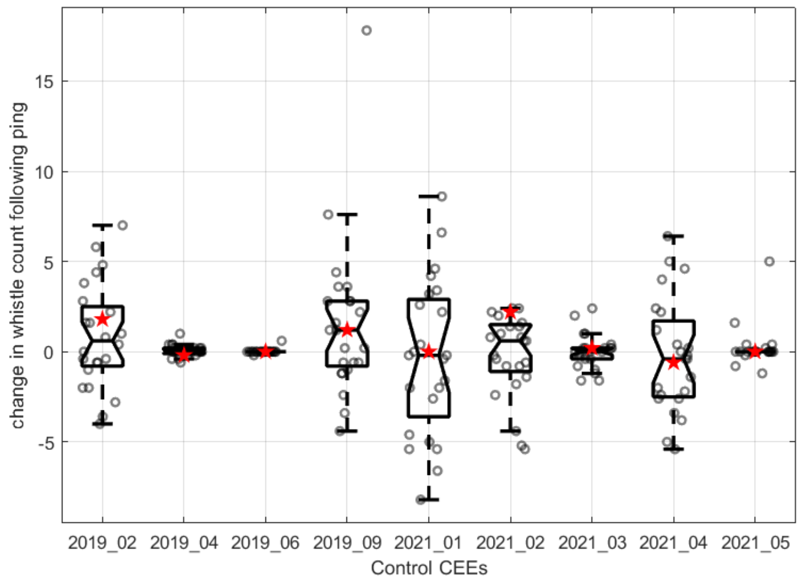

Commented [Anon.3]: It would be a bit more useful comparison-wise if the Y-axis in the bottom figure was the same scale as the top figure.

Figure S2.1. Boxplots of the change in whistle count from the 5 seconds before to the 5 seconds following each of the 24 pings for all CEEs (MFAS and Controls). Boxplot shows median, 25th, and 75th percentiles, with raw whistle count changes as open gray circles. The change following the first ping is shown as a red star.

S3 Appendix: Raw whistle counts

Plots of raw per-second whistle counts for all included CEEs. Red dashed line indicates start of exposure period with dashed grey lines indicating the timing of each ping (in an MFAS exposure) or ghost ping (in a Control exposure). Solid gray vertical line indicates start of post-exposure period.

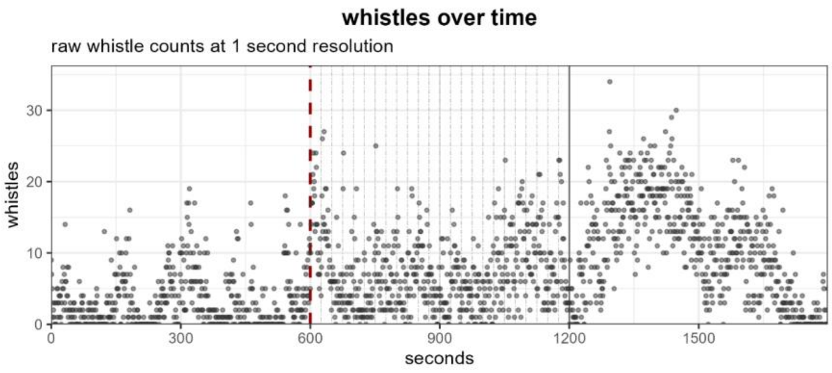

2019_01

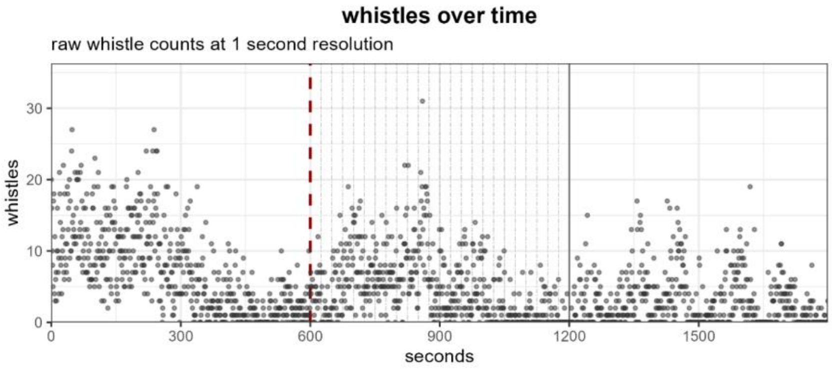

2019_02

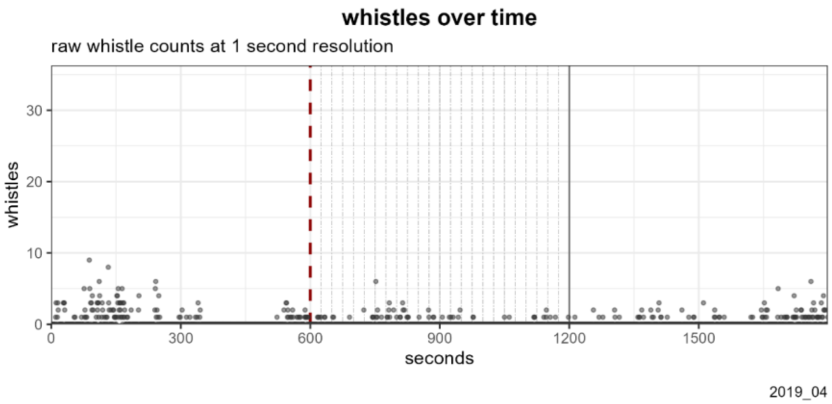
whistles over time
raw whistle counts at 1 second resolution
whistles
seconds
2019_04

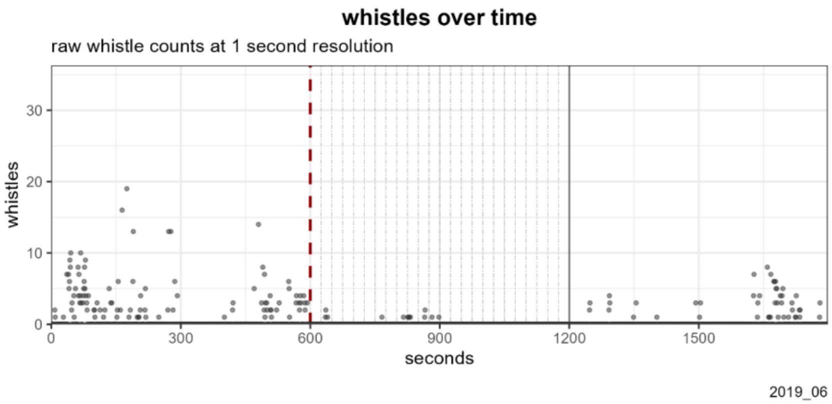
whistles over time
raw whistle counts at 1 second resolution
whistles
seconds
2019_06

whistles over time

raw whistle counts at 1 second resolution

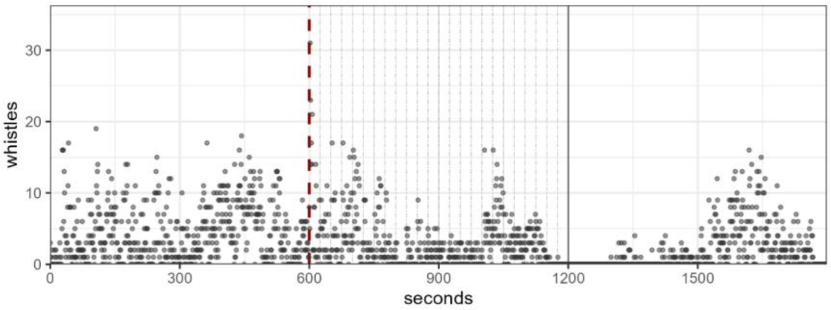

2019_07

whistles over time

raw whistle counts at 1 second resolution

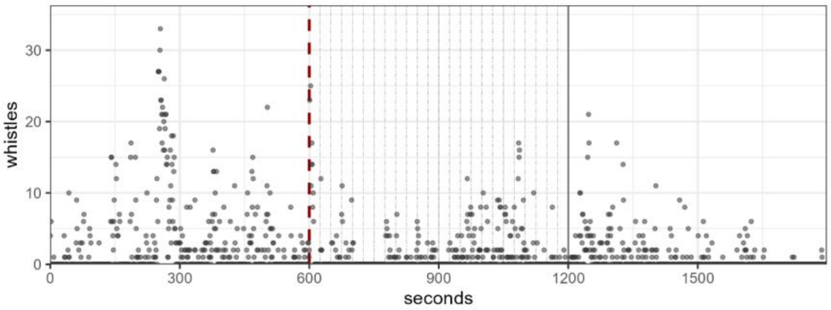

2019_08

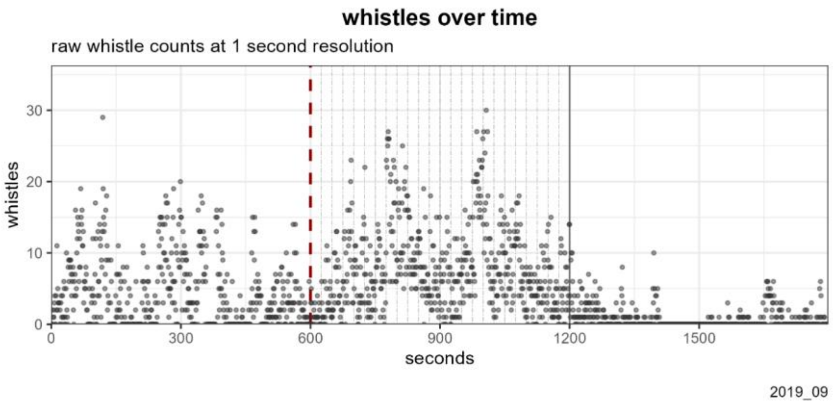
whistles over time
raw whistle counts at 1 second resolution
whistles
seconds
0
10
20
30
0
300
600
900
1200
1500
2019_09

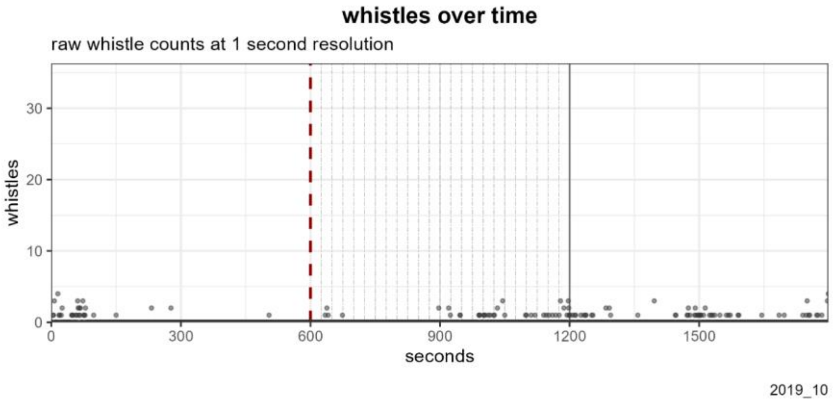
whistles over time
raw whistle counts at 1 second resolution
whistles
seconds
0
10
20
30
0
300
600
900
1200
1500
2019_10

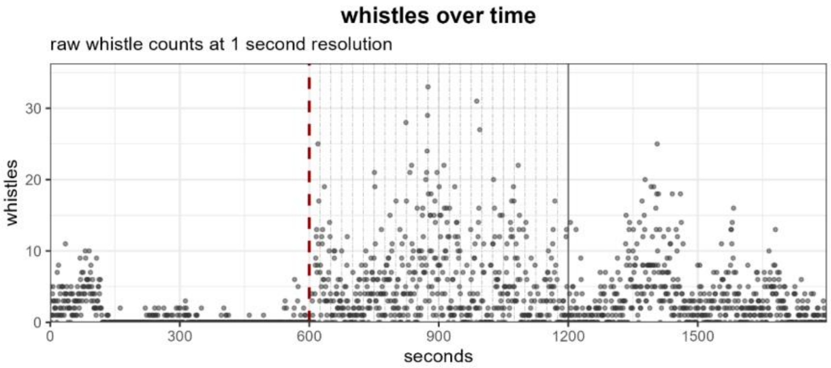

2021_01

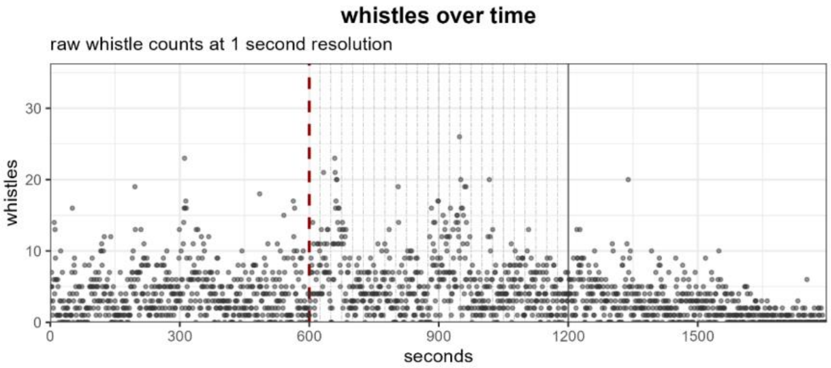

2021_02

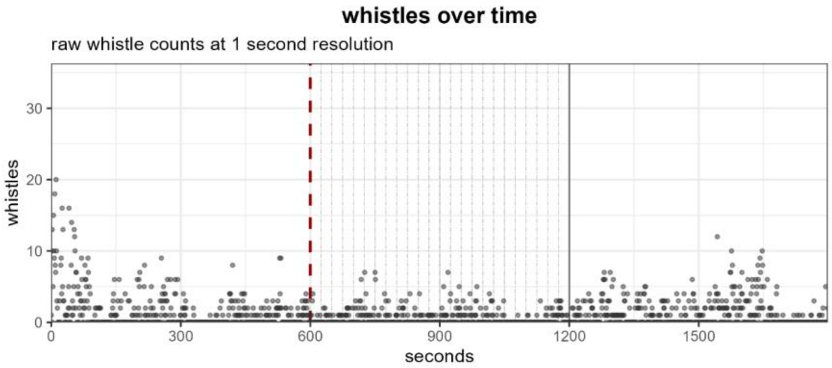

2021_03

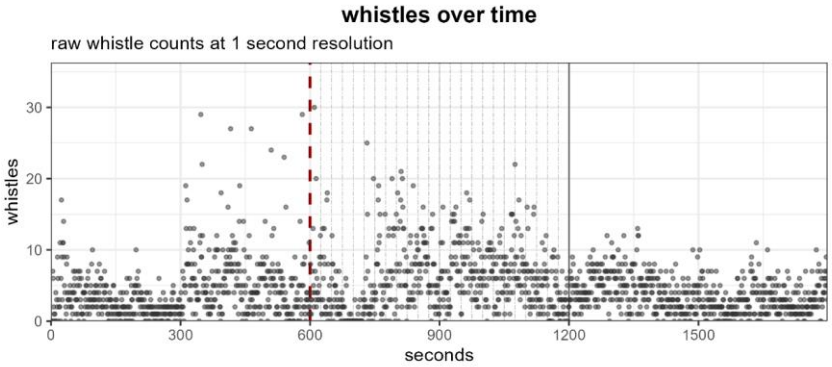

2021_04

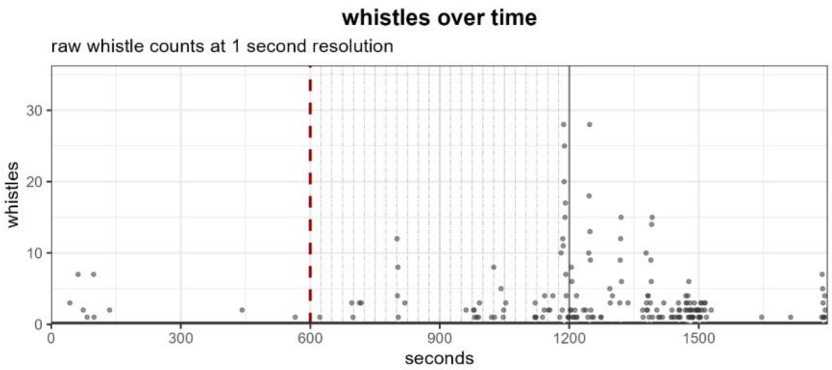

2021_05

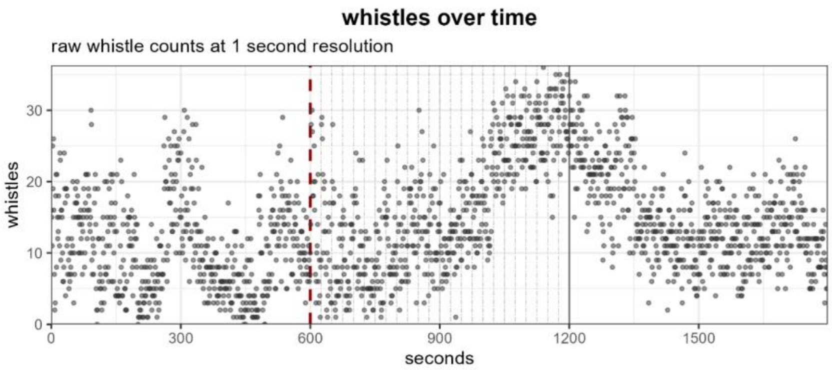

2021_08

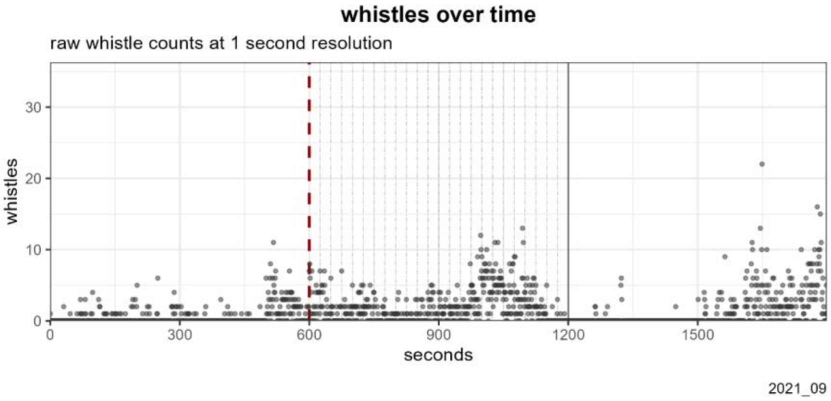

whistles over time
raw whistle counts at 1 second resolution
whistles
seconds
0
10
20
30
300
600
900
1200
1500
2021_09

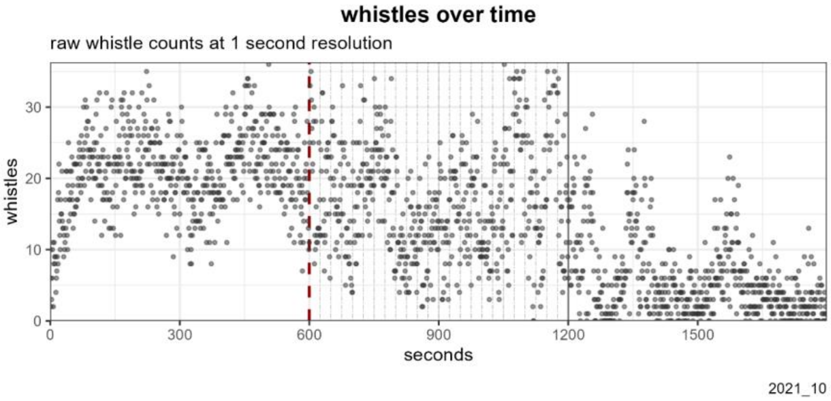

whistles over time
raw whistle counts at 1 second resolution
whistles
seconds
0
10
20
30
300
600
900
1200
1500
2021_10

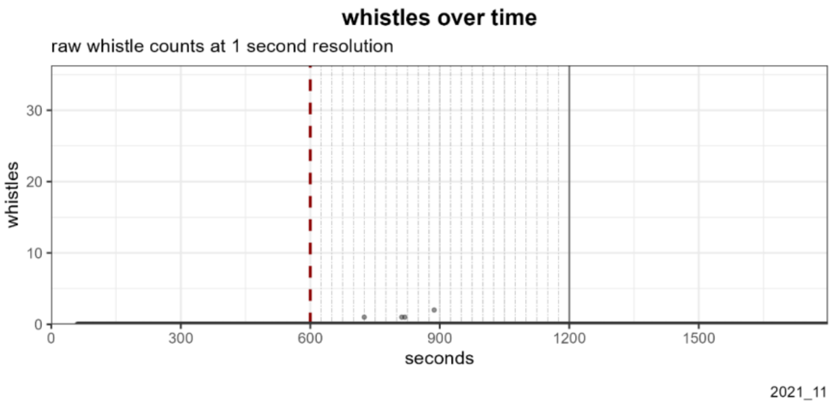
whistles over time
raw whistle counts at 1 second resolution
whistles
0
10
20
30
0
300
600
900
1200
1500
seconds
2021_11

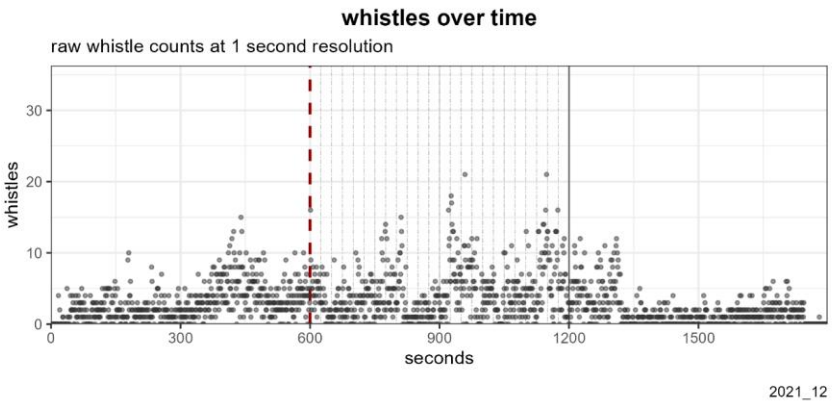

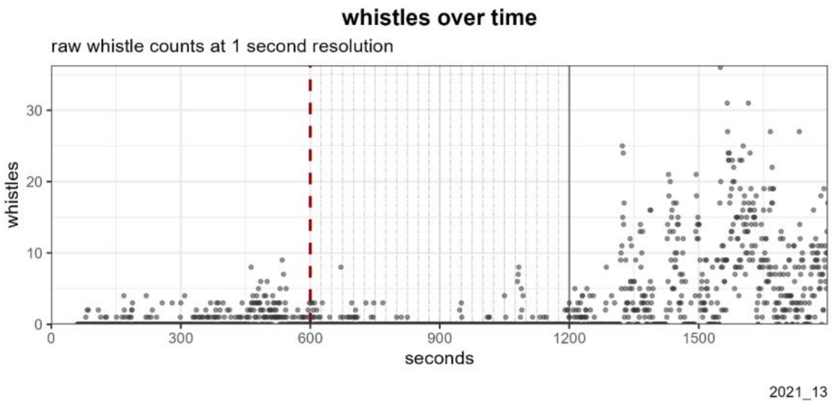

References

1. Nikoloulopoulos AK, Karlis D. On modeling count data: a comparison of some well-known discrete distributions. *JSCS.* 2008;78(3):437-57.
