# Supplementary material for: Common dolphin whistle responses to experimental mid-frequency sonar
Source: PLoS One. 2024 Apr 26;19(4):e0302035. doi: 10.1371/journal.pone.0302035 (PMC11051594; doi:10.1371/journal.pone.0302035)
Supplement: Attachment — Submitted filename: PONE-D-23-38783_R1_revised comments.pdf [file pone.0302035.s008.pdf]

PLOS ONE

Common dolphin whistle responses to experimental mid-frequency sonar

--Manuscript Draft--

**Manuscript Number:**	PONE-D-23-38783R1
**Article Type:**	Research Article
**Full Title:**	Common dolphin whistle responses to experimental mid-frequency sonar
**Short Title:**	Dolphin acoustic response to sonar
**Corresponding Author:**	Caroline Casey<br>UCSC: University of California Santa Cruz<br>Santa Cruz, UNITED STATES
**Keywords:**	Bioacoustics: Common dolphins; (Delphinus delphis); (Delphinus bairdii); Behavioral response study; Navy sonar; Acoustic behavior
**Abstract:**	Oceanic delphinids around naval operations are regularly exposed to intense military sonar broadcast within the frequency range of their hearing. However, empirically measuring the impact of sonar on the behavior of highly social, free-ranging dolphins is challenging. Additionally, baseline variability or the frequency of vocal state-switching among social oceanic dolphins during undisturbed conditions is lacking, making it difficult to attribute changes in vocal behavior to anthropogenic disturbance. Using a network of drifting acoustic buoys in controlled exposure experiments, we investigated the effects of mid-frequency (3-4 kHz) active sonar (MFAS) on whistle production in short-beaked (Delphinus delphis delphis) and long-beaked common dolphins (Delphinus delphis bairdii) in southern California. Given the complexity of acoustic behavior exhibited by these group-living animals, we conducted our response analysis over varying temporal windows (10 min – 5 s) to describe both longer-term and instantaneous changes in sound production. We found that common dolphins exhibited acute and pronounced changes in whistle rate in the 5 s following exposure to simulated Navy MFAS. This response was sustained throughout sequential MFAS exposures within experiments simulating operational conditions, suggesting that dolphins may not habituate to this disturbance. These results indicate that common dolphins exhibit brief yet clearly detectable acoustic responses to MFAS. They also highlight how variable temporal analysis windows – tuned to key aspects of baseline vocal behavior as well as experimental parameters related to MFAS exposure – enable the detection of behavioral responses. We suggest future work with oceanic delphinids explore baseline vocal rates a-priori and use information on the rate of change in vocal behavior to inform the analysis time window over which behavioral responses are measured.
**Order of Authors:**	Caroline Casey
	Selene Fregosi
	Julie Oswald
	Vincent Janik
	Fleur Visser
	Brandon Southall
**Response to Reviewers:**	Dear Vitor Hugo Rodrigues Paiva and the editorial team,<br><br>We greatly appreciate your response to our submitted manuscript (Common dolphin whistle response to experimental mid-frequency sonar – PONE-D-23-38783). It was helpful and gratifying to receive reviewer comments on our study that were so thoughtful and meticulous. We appreciate that the editorial team believes that this is a unique paper that should be of broad interest to the readers of PLOS One.<br><br>We have spent considerable time reflecting on and revising this manuscript based on the detailed recommendations of each reviewer. These changes can be seen in the "Revised Manuscript with Track Changes" document.<br>With respect to the specific Journal Requirements, we have made the following

Powered by Editorial Manager® and ProduXion Manager® from Aries Systems Corporation

requested changes:
1. Please ensure that your manuscript meets PLOS ONE's style requirements, including those for file naming. The PLOS ONE style templates can be found at https://journals.plos.org/plosone/s/file?id=wjVg/PLOSOne_formatting_sample_main_body.pdf and
https://journals.plos.org/plosone/s/file?id=ba62/PLOSOne_formatting_sample_title_authors_affiliations.pdf
The Title, Author, and affiliations have been updated. Additionally, we have carefully gone through PLOS ONE's style requirements, and have made several changes to the manuscript.
2. Thank you for stating the following financial disclosure:
"Funding for this project was provided by the U.S. Navy's Office of Naval Research (Award Numbers N000141713132, N0001418IP-00021, N000141712887, N000141912572). "
Please state what role the funders took in the study. If the funders had no role, please state: ""The funders had no role in study design, data collection and analysis, decision to publish, or preparation of the manuscript.""
If this statement is not correct you must amend it as needed.
Please include this amended Role of Funder statement in your cover letter; we will change the online submission form on your behalf.
This statement is correct. The funders had no role in study design, data collection and analysis, decision to publish, or preparation of the manuscript. We have removed the funding information from the Acknowledgements section and can re-include this in our cover letter.
3. Thank you for stating the following in the Acknowledgments Section of your manuscript:
"Funding for this project was provided by the U.S. Navy's Office of Naval Research (Award Numbers N000141713132, N0001418IP-00021, N000141712887, N000141912572). "
We note that you have provided funding information that is not currently declared in your Funding Statement. However, funding information should not appear in the Acknowledgments section or other areas of your manuscript. We will only publish funding information present in the Funding Statement section of the online submission form.
Please remove any funding-related text from the manuscript and let us know how you would like to update your Funding Statement. Currently, your Funding Statement reads as follows:
"Funding for this project was provided by the U.S. Navy's Office of Naval Research (Award Numbers N000141713132, N0001418IP-00021, N000141712887, N000141912572). "
Please include your amended statements within your cover letter; we will change the online submission form on your behalf.
We apologize for the confusion. We have removed the funding statement from the main body of the manuscript. The funding statement that you have here is correct.
4. We note that Figure 1 in your submission contain [map/satellite] images which may be copyrighted. All PLOS content is published under the Creative Commons Attribution License (CC BY 4.0), which means that the manuscript, images, and Supporting Information files will be freely available online, and any third party is permitted to access, download, copy, distribute, and use these materials in any way, even commercially, with proper attribution. For these reasons, we cannot publish previously copyrighted maps or satellite images created using proprietary data, such as Google software (Google Maps, Street View, and Earth). For more information, see our copyright guidelines: http://journals.plos.org/plosone/s/licenses-and-copyright.

We require you to either (1) present written permission from the copyright holder to publish these figures specifically under the CC BY 4.0 license, or (2) remove the figures from your submission:

a. You may seek permission from the original copyright holder of Figure 1 to publish the content specifically under the CC BY 4.0 license.

We recommend that you contact the original copyright holder with the Content Permission Form (http://journals.plos.org/plosone/s/file?id=7c09/content-permission-form.pdf) and the following text:

“I request permission for the open-access journal PLOS ONE to publish XXX under the Creative Commons Attribution License (CCAL) CC BY 4.0 (http://creativecommons.org/licenses/by/4.0/). Please be aware that this license allows unrestricted use and distribution, even commercially, by third parties. Please reply and provide explicit written permission to publish XXX under a CC BY license and complete the attached form.”

Please upload the completed Content Permission Form or other proof of granted permissions as an ""Other"" file with your submission.

In the figure caption of the copyrighted figure, please include the following text: “Reprinted from [ref] under a CC BY license, with permission from [name of publisher], original copyright [original copyright year].”

b. If you are unable to obtain permission from the original copyright holder to publish these figures under the CC BY 4.0 license or if the copyright holder’s requirements are incompatible with the CC BY 4.0 license, please either i) remove the figure or ii) supply a replacement figure that complies with the CC BY 4.0 license. Please check copyright information on all replacement figures and update the figure caption with source information. If applicable, please specify in the figure caption text when a figure is similar but not identical to the original image and is therefore for illustrative purposes only.
The following resources for replacing copyrighted map figures may be helpful:

USGS National Map Viewer (public domain): http://viewer.nationalmap.gov/viewer/
The Gateway to Astronaut Photography of Earth (public domain): http://eol.jsc.nasa.gov/sseop/clickmap/
Maps at the CIA (public domain): https://www.cia.gov/library/publications/the-world-factbook/index.html and https://www.cia.gov/library/publications/cia-maps-publications/index.html
NASA Earth Observatory (public domain): http://earthobservatory.nasa.gov/
Landsat: http://landsat.visibleearth.nasa.gov/
USGS EROS (Earth Resources Observatory and Science (EROS) Center) (public domain): http://eros.usgs.gov/#
Natural Earth (public domain): http://www.naturalearthdata.com/
We have redesigned the figure based on the map provided by NASA Earth Observatory (public domain) that complies with the CC BY 4.0 license. The updated Figure 1. is now reflected in the manuscript. We have also added the following to the figure caption. “The map was inspired by images obtained from the NASA Earth Observatory (public domain), is not drawn to scale, and is for illustrative purposes only”.

5. Please include captions for your Supporting Information files at the end of your manuscript, and update any in-text citations to match accordingly. Please see our Supporting Information guidelines for more information: http://journals.plos.org/plosone/s/supporting-information.
This has been done.
6. Please review your reference list to ensure that it is complete and correct. If you have cited papers that have been retracted, please include the rationale for doing so in the manuscript text, or remove these references and replace them with relevant current references. Any changes to the reference list should be mentioned in the rebuttal letter that accompanies your revised manuscript. If you need to cite a retracted article, indicate the article’s retracted status in the References list and also include a citation and full reference for the retraction notice.

We have carefully gone through our references and have updated the formatting. We do not believe we have any retracted references in our reference list.

Additionally, all the reviewers made minor suggestions to strengthen the manuscript, and we have addressed each in turn. Our specific responses to reviewers are provided below. Given these changes, we hope that PLOS One finds our manuscript suitable for publication. Please do not hesitate to contact me if you require any additional information.

Powered by Editorial Manager® and ProduXion Manager® from Aries Systems Corporation

Specific comments to reviewers can be found below:

Reviewer #1: The information contained in the manuscript is useful. One has to wonder whether the results will stand if/when additional data are obtained and n is increased.
We certainly aim to address this in the future in our ongoing BRS efforts and appreciate the supportive feedback.

The information itself is straightforward, but the manuscript is not as clear as it could be. There also were some inaccuracies, errors, inconsistencies, missing words, and formatting issues. The manuscript should be reviewed/revised editorially, since PLOS ONE does not use a copy editor.

Some of the issues included—
• Short- and long-beaked common dolphins are separate subspecies, not species. Their correct scientific names must be used.
Thank you for bringing this to our attention. This has been corrected throughout the manuscript based on the Marine Mammal Society Taxonomic list of marine mammal species subspecies.

• The units for source level and received level must be correct and complete (dB re 1 μPa at 1 m and dB re 1 μPa, respectively) and should include rms, when applicable
Thank you, this has been adjusted throughout the manuscript where appropriate.

• 10 MFAS CEEs were denoted in Table 1, but the text in the manuscript specified 9 CEEs in numerous instances.
We apologize for the confusion on this. We realize that there were some details here that needed to be resolved. Specifically, we had to exclude one of the CEEs (2021_11) from our modeling work because of the low number of whistles detected during this experiment. The following information has been added to the text of the manuscript for clarity: "For the changepoint analysis, all 10 MFAS experiments were included. However, for the assessment of changes in whistle behaviors across different time scales, CEE 2021_11 (conducted with long-beaked common dolphins) had to be excluded because the overall lack of detected whistles could not be successfully modeled. Consequently, the modeling results include nine controls and nine MFAS experiments. "

• The tense of nouns and verbs should be the same within a given sentence, as should the person.
• Commas should be used consistently and correctly throughout. In some instances, commas were not used when they should have been (i.e., after introductory clauses).
• Hyphens should not be used for nouns (i.e., 'in 5 sec' instead of 'in 5-sec') but should be used for adjectives (i.e., 'in a 5-sec bin' instead of 'in a 5 sec bin').
• Abbreviations should be used consistently (i.e., second, sec, and s were all used).
• "Table" should be capitalized when cited in the text.
• References should be cited in the correct order (numerically ascending), format (when the name also is used in the text), and with the correct punctuation (brackets [] instead of parentheses () for PLOS ONE).
• Capitalization for headings/headers and bolding of captions should be consistent.
• Extra spaces should be deleted within and between sentences.
We thank the reviewer for these detailed comments. We have carefully gone through the manuscript and have addressed each of their edits. These changes can be seen within the "Revised Manuscript with Track Changes" document.

Also, information regarding the IACUC review and approval was missing in various sections of the manuscript.
We apologize for this oversight. Our IACUC information has been added to the Acknowledgments section. The text now reads: "Additionally, all activities reported in this study were reviewed and approved by the Institutional Animal Care and Use Committee (IACUC Protocol No: CRC-2021-AUP-06, CRC-2021-AUP-08)."

Please see the pdfs for specific comments and questions regarding the manuscript and supplementary docs.
Many suggestions were made directly in an associated PDF (Reviewer #1). Those

Powered by Editorial Manager® and ProduXion Manager® from Aries Systems Corporation

changes (unless noted otherwise below) were made to the revised version of the manuscript and greatly improved the clarity. We thank Reviewer 1 for the time they spent reviewing our work.
-With respect to their comments about the consistency of the y-axis in Fig. 4b and Fig 4c, we decided to leave the figure as is, as the intention of the figure is to highlight the relative changes between the pre-exposure and exposure period for each individual plot, rather than compare across the plots. Keeping the axes as they are allows maximum resolution for across period comparisons.
-We will upload the data to NCEI as well since the project was funded by ONR.
-With respect to their comment about the validity of pooling the two subspecies acoustic data for baseline analysis, we decided not to pursue this approach since they regularly occur in mixed groups, and our sample size of Delphinus delphis delphis was relatively small. Further work looking at subspecies-specific differences in whistle production is still needed but is not within the scope of this paper.
Reviewer #2: Casey et al. aimed to measure the impact of Navy MFAS sonar on free-ranging dolphins in comparison to baseline variability data of acoustics. They utilize a network of acoustic buoys and controlled exposure experiments measure acoustic disturbance of free ranging short-beaked and long-beaked common dolphin whistles. CEEs were conducted off the coast of Santa Catalina Island and data were analyzed for vocal state changes. The authors found a significant vocal state change in the 5 seconds post CEEs. Interestingly, they give information on potential habituation of the animals as well.
Overall, it is a well written paper looking at vocal state changes as a tool for measuring disturbance. There are a few errors and areas that transition phrases are redundant. There are some areas where the authors have accidentally put in an extra space between words.
Thank you for this positive review of our manuscript. To address reviewers 1 and 2's comments, we have gone through the manuscript carefully to correct any editorial errors. We hope that this makes things easier to follow and clearer for the reader.

Line 19: Considering we do not have data that states that millions hear and are affected by Navy MFAS in particular, I would suggest rephrasing this first sentence or adding a citation.
We agree and have changed this sentence to be more general.

Line 222-223: While the author's state that the distance between the drone and the octocopter were estimated every thirty minutes, it would be beneficial for the authors to state how high the drone was flying on average.
Thank you for this suggestion. We have added the following details to the text to address both reviewer 2 and 3's point: "The animals' location was known from an associated octocopter drone flight (APO-42, Aerial Imaging Solutions) that utilized a micro 4/3 digital camera (Olympus E-PM2) and 25 mm lens (Olympus M. Zuiko F1.8) mounted to a gimbal. The octocopter flew at approximately 60 m directly above the dolphins to provide sufficient pixel resolution while also decreasing the potential for disturbance (see [34] for details)."

Line 427: Consider deleting "however" after Note.
We appreciate this suggestion and we have made this change.

Line 448: consider replacing en dash with an em dash.
We appreciate this suggestion and we have made this change.

Line 449: 10 MFAS CEEs were conducted but only 2 in Dolphinus delphis, why? I would suggest at least one line of explanation on this.
The reviewer here caught an important mistake in our text. While Table 1. reflected the correct proportion of CEEs for both sub-species (7 of 10 with D. d. bairdii and 3 of 10 with D. d. delphis), the text did not. The text is now consistent with the proportion of MFAS CEEs conducted with each sub-species presented in Table 1.
To address their question as to why we conducted more MFAS cees with D. d. bairdii – The total length of this project spanned over 4 years (2017, 2018, 2019, 2021). During that time, we did balance MFAS playbacks equally across subspecies. However, for this paper, we only decided to include data from 2019 and 2021 because of the consistency in acoustic methodology in the field during these two years. We happened to conduct more experiments with D. d. bairdii during the later years of this project,

which is why they are represented more here in this paper. We do not agree that we should include this in the text of the manuscript (as it is a bit verbose), so we have left it as is. We could add that D. d. bairdii was encountered more often and is therefore making up most of the data if the editors think this is helpful. Please advise.

Line 483: cee is not capitalized in this line but is in front of the word type in the line before. I would suggest making this congruent in the paragraph.
This has been corrected in the manuscript.

Line 618: There is an extra space between the last word of the sentence and the period.
This has been corrected in the manuscript.

Line 647: There is an extra space between the words “sizes” and “where”.
This has been corrected in the manuscript.
Reviewer #3: In this manuscript, the authors investigate the impact of mid-frequency active sonar (MFAS) on the whistle production of common dolphins in Southern California. The study uses a network of drifting acoustic buoys in a controlled exposure experiment to analyze the dolphins' acoustic reactions to sonar exposure over various time frames, to understand both longer-term and immediate changes in sound production.

The manuscript is detailed and informative but can be challenging to follow due to its complexity. Simplifying the language or reorganizing the data presentation could enhance readability. The authors do a good job arguing that the main objective is to clearly understand variability in baseline and examine differences in whistle count compared to baseline. However, some aspects are still unclear and could help the readability and better understanding of the scale.
We have taken into consideration this comment and have tried to make the manuscript clearer and more concise throughout. We hope that this satisfies reviewer 3’s comment.

To better grasp the scope of the study, it would be helpful to know: How much total recording hours were collected? How much was actually used in the analysis?
Up to 30 minutes of acoustic data per CEE was used in the baseline vocal activity (10-minute pre-exposure, 10-minute exposure, and 10-minute post-exposure periods) and 20 minutes of recordings per CEE was used in the response analysis (10-minute pre-exposure and 10-minute exposure periods).
Due to the nature of working with dynamic groups of free-ranging dolphins, the amount of additional recording before or after the actual CEE is highly variable (from just a few minutes before, to maybe 20 minutes or more after the CEE ended if it the animals moved a lot and the boat had to transit further back to pick up the buoy). Additionally, because of what we know about transmission loss, we used the 1600 m cut off considering a focal group within the range of the recordings; we may have had underwater recordings during the CEE time, or outside the nominal 30-minute CEE, but when the animals were 2+ km away those recordings were not considered usable. Because of this, we just analyzed up to the 30 minutes of the actual CEE, and only recordings from when the animals were within 1600 m of the recorder.
To address the suggestion for better grasping the scope, we added the following text to line 233-244:
"After accounting for this distance cut off, 9.25 total hours of recordings remained and were used in subsequent analysis."

Was the use of WMD validated with manually labeled data? AND
Although automated tools have gotten better, they still often bring a large amount of errors compared to manual labeling which is still recommended depending on the task. The validity of the results would be improved if the authors could use a subset of their data for manual analysis as a reliability measure of the software approach.
We appreciate these two comments and have combined our response here as they are closely related. We acknowledge that automated tools are imperfect and ideally would benefit from systematic manual review and validation. Our initial approach for this project did include manual review of all whistles, but we quickly realized this was not a feasible approach with the amount of data and very large numbers of whistles that we recorded. We had three analysts go through and count individual whistles from a very

small portion of these same recordings and found large variability in each analyst's manual assessment. The recordings often contain overlapping and/or 10s of whistles per second, which made accurate counts unreliable. While automated approaches have the same difficulties with overlapping whistles and periods of dense whistling, we opted to use an automated approach instead to remove some of the subjectiveness and biases introduced by different analyst. Our idea is that although an automated approach is imperfect, it is consistently imperfect within a 30-minute CEE, where recording conditions are similar. The text under the header ”Quantifying whistle production and variability” aims to justify this approach within the text. To try to further clarify, we changed the term 'whistle counts' to 'whistle detections' throughout the body of the manuscript, and added the following to this section:

"A qualitative assessment of detector performance within each CEE ensured that variation in whistle detections accurately reflected variation in whistle activity observed in the spectrograms."

We qualitatively reviewed the WMD outputs to ensure we were getting valid results and that the observed counts reflected what we saw in the spectrograms. We observed that the WMDtends to fragment whistles that a human would likely consider one whistle, and its performance decreases as whistling increases. Any potential bias from this increased variance in performance with increased whistle counts is accounted for in the covariance matrix of the modeling, where we allowed variance to differ by overall whistle count. False positives were rare and occurred in the form of harmonics of the projected sonar signal or were from an unknown linear noise of some sort that occasionally occurred on one of the CEEs; we manually removed these false positives.

The abstract effectively summarizes a lot of information but could be enhanced by a clearer statement of the main findings beyond “exhibited an acute and dramatic change in acoustic behavior in the 5-seconds following exposure to experimental” Is the metrics only a count of whistle per overlapping windows?

We have reworded the abstract to make the findings of the study clearer.

L.157 - To what extent are the animals already accustomed to the signals. For how long? How many generations? What differences in dispersion, sample rate, etc, between actual MFAS pings and experimental conditions could affect the results?

Common dolphin habitat overlaps with the Southern California Range Complex, which is frequently used by the US Navy as the site of MFAS training exercises. Active sonar of some type, including the most powerful MFAS systems, is used in this area daily, often multiple times a day. How many years this has occurred is not information that is publicly available. The exact timing and repetition of sonar produced in this area is unknown, but presumably, these animals have been exposed to MFAS multiple times throughout their lives (lifespan ~ 30 years). The playback stimuli that we used are categorically very similar (with respect to timing – once every 25 seconds) to certain Navy sources (i.e., helo-dipping sonar). The source itself and the positioning of the source boat relative to the animals during our experiments were designed to mimic the received levels animals are likely exposed to during actual helo-dipping training exercises.

L.180 - Regarding the use of drones for the experiment, specific details such as the type of drones, their number, operational height, and potential disturbances (visual or sonic) at the water level would be valuable.

Thank you for this suggestion. We have added the following details to the text to address this point: “The animals’ location was known from an associated octocopter drone flight (APO-42, Aerial Imaging Solutions) that utilized a micro 4/3 digital camera (Olympus E-PM2) and 25 mm lense (Olympus M. Zuiko F1.8) mounted to a gimbal. The octocopter flew at approximately 60 m directly above the dolphins to provide sufficient pixel resolution while also decreasing the potential for disturbance.”

L.180-190 - Good methodology observation

Thank you.

Powered by Editorial Manager® and ProduXion Manager® from Aries Systems Corporation

L.227 - More clarification needed
We hope that the above text concerning the details of the octocopter is sufficient to address this point.

L.260 - Good methodological point
Thank you

L.270: Why use different recording hardware? Any baseline to address differences in recordings?
Different recording hardware was used because of cost/available resources over the course of this study. In the first years of this work, we had access to 2 SNAP recorders and 1 SoundTrap. In the time between the two field years, we were able to acquire two additional SoundTraps, which we found to be more reliable and easier to use in the field than the SNAPS. We qualitatively assessed the quality of the recordings (electronic noise, frequency response) and didn't observe any noticeable differences that we felt limited us from being able to use data from both recorder types. We standardized the Pamguard analysis approach to account for the differences in sample rate, and the detection threshold is based on SNR, so differences in system sensitivity are not an issue.

L.282: More clarification needed
Lines 281-208 of the original submitted manuscript read: "Whistles were exported from PAMGuard using the PAMGuard MATLAB tools (https://github.com/PAMGuard/PAMGuardMatlab) and R package `PAMPal` (40). MFAS harmonics were removed, and whistles were quantified at 1-second resolution." We are unsure what more clarification is needed here, however we reworded and added a few additional words to try to improve clarity. It now reads "Detected whistles were automatically exported from the PAMGuard detection database using the PAMGuard MATLAB tools (https://github.com/PAMGuard/PAMGuardMatlab). Annotated MFAS harmonics were removed using R package `PAMmisc` in R version 4.3.1 [33,34]. Whistles were quantified at 1-s resolution; because whistles are often longer than 1 s in duration, the total number of whistles starting within a 1-s bin was counted, providing a metric for whistle activity as whistles detections per second." We hope this is sufficient to address the reviewers needs.

Fig 1: It would be beneficial to add scale information to the figure.
This schematic is not drawn to scale. Instead, we attempted to highlight the configuration and experimental design used during playback experiments. Therefore, we have left out a scale bar from this figure and have added the sentence "This schematic is not drawn to scale" to the figure caption. (Lines 211-213).

L.577 - The lack of elevation in whistle count during the 1.6 s signal broadcast is intriguing. Further explanations on this observation and its implications would be insightful. Was the sonar frequency removed from the recorded audio? Extend on the possible use of full duplex vs single duplex for future experiments.
We certainly agree that the lack of whistles present during the 1.6s sonar signal is interesting, and we present some possible explanations in the discussion. For example, lines 572-581 read:
" The lack of whistle production during sonar transmission may be a tactic for reducing acoustic interference and masking, which has been shown to impact the detection, discrimination, and localization of relevant signals [41]. If the signal is predictable (as in our experiment), then animals should be able to adjust the timing of sound production to limit communication to periods in which noise is reduced [e.g., 41-44]. The ability of dolphins to learn the timing of intermittent noise has previously been demonstrated by Finneran et al., 2023 [41], who showed that individuals can modify their hearing sensitivity before the onset of predictably timed impulses, presumably to mitigate adverse auditory impacts."
Given that we didn't explicitly test this phenomenon, we have refrained from elaborating more on this topic.
We only detected whistles between 5-20 kHz, which excluded the frequency bands of the sonar signal itself. Further, we manually removed any harmonics from the sonar between 5-20 kHz that were detected incorrectly as whistles (this is already mentioned in the methods). We are a little unclear about the reviewer's question regarding "full

	duplex versus single duplex for future experiments," however I believe that we addressed this comment above.<br><br>L.590 - Interesting hypothesis, how could this be tested in the future?<br>We agree that this is an interesting point, although it would be difficult to test using the acoustic data alone. We have added the following sentence to expand upon this idea and how it is currently being explored by our research group: "In these cases, there are often a few key individuals that catalyze the collective behavior of the rest of the group. While it would be difficult to evaluate this process using acoustic data alone, concurrent video data collected from the associated drone flights is currently being evaluated to explore the spatial movement patterns of the group and identify those individuals that successfully initiate changes in group movement."<br><br>L.607 - This point warrants earlier discussion in the manuscript for better context. Text<br><br>We did mention that the animals tested in this study occur in Navy operational areas in the methods section.<br><br>L.618 - Extra space before comma.<br><br>This has been resolved.<br><br>How do the two species compare in terms of vocal reactions?<br><br>Due to the relatively small sample size, differences in species response were not quantitatively tested. Anecdotally, one of the three MFAS experiments that were conducted with D. d. delphis showed a change in whistle production relative to baseline at the 5-second scale, and the remaining two experiments did not show a response. While interesting, this is inconclusive and deserves further investigation.
**Additional Information:**	
**Question**	**Response**
**Financial Disclosure**<br><br>Enter a financial disclosure statement that describes the sources of funding for the work included in this submission. Review the submission guidelines for detailed requirements. View published research articles from *PLOS ONE* for specific examples.<br><br>This statement is required for submission and **will appear in the published article** if the submission is accepted. Please make sure it is accurate.	Yes

**Funded studies**<br>Enter a statement with the following details:<br>• Initials of the authors who received each award<br>• Grant numbers awarded to each author<br>• The full name of each funder<br>• URL of each funder website<br>• Did the sponsors or funders play any role in the study design, data collection and analysis, decision to publish, or preparation of the manuscript?<br><br>Did you receive funding for this work?	
Please add funding details.<br>as follow-up to "**Financial Disclosure**<br><br>Enter a financial disclosure statement that describes the sources of funding for the work included in this submission. Review the submission guidelines for detailed requirements. View published research articles from *PLOS ONE* for specific examples.<br><br>This statement is required for submission and **will appear in the published article** if the submission is accepted. Please make sure it is accurate.<br><br>**Funded studies**<br>Enter a statement with the following details:<br>• Initials of the authors who received each award<br>• Grant numbers awarded to each author<br>• The full name of each funder<br>• URL of each funder website<br>• Did the sponsors or funders play any role in the study design, data collection and analysis, decision to publish, or preparation of the manuscript?<br><br>Did you receive funding for this work?"	Funding for this project was provided by the U.S. Navy's Office of Naval Research (Award Numbers N000141713132, N0001418IP-00021, N000141712887, N000141912572). . The funders had no role in study design, data collection and analysis, decision to publish, or preparation of the manuscript.
Please select the country of your main research funder (please select carefully as in some cases this is used in fee calculation).	UNITED STATES - US

as follow-up to "**Financial Disclosure**<br><br>Enter a financial disclosure statement that describes the sources of funding for the work included in this submission. Review the submission guidelines for detailed requirements. View published research articles from *PLOS ONE* for specific examples.<br><br>This statement is required for submission and **will appear in the published article** if the submission is accepted. Please make sure it is accurate.<br><br>**Funded studies**<br>Enter a statement with the following details:<br>• Initials of the authors who received each award<br>• Grant numbers awarded to each author<br>• The full name of each funder<br>• URL of each funder website<br>• Did the sponsors or funders play any role in the study design, data collection and analysis, decision to publish, or preparation of the manuscript?<br><br>Did you receive funding for this work?"	
**Competing Interests**<br><br>Use the instructions below to enter a competing interest statement for this submission. On behalf of all authors, disclose any competing interests that could be perceived to bias this work—acknowledging all financial support and any other relevant financial or non-financial competing interests.<br><br>This statement is required for submission and **will appear in the published article** if the submission is accepted. Please make sure it is accurate and that any funding sources listed in your Funding Information later in the submission form are also declared in your Financial Disclosure statement.	The authors have declared that no competing interests exist.

Powered by Editorial Manager® and ProduXion Manager® from Aries Systems Corporation

View published research articles from *PLOS ONE* for specific examples.<br><br>**NO authors have competing interests**<br><br>Enter: *The authors have declared that no competing interests exist.*<br><br>**Authors with competing interests**<br><br>Enter competing interest details beginning with this statement:<br><br>*I have read the journal's policy and the authors of this manuscript have the following competing interests: [insert competing interests here]*<br><br>* typeset	
**Ethics Statement**<br><br>Enter an ethics statement for this submission. This statement is required if the study involved:<br><br>• Human participants<br>• Human specimens or tissue<br>• Vertebrate animals or cephalopods<br>• Vertebrate embryos or tissues<br>• Field research<br><br>Write "N/A" if the submission does not require an ethics statement.<br><br>General guidance is provided below. Consult the submission guidelines for detailed instructions. **Make sure that all information entered here is included in the Methods section of the manuscript.**	Animal observations, octocopter flights over dolphins, close approaches, and CEEs were conducted under NMFS permits 19116 and 19091. Additionally, all activities reported in this study were reviewed and approved by the Institutional Animal Care and Use Committee (IACUC Protocol No: CRC-2021-AUP-06, CRC-2021-AUP-08).

Powered by Editorial Manager® and ProduXion Manager® from Aries Systems Corporation

**Format for specific study types**<br><br>**Human Subject Research (involving human participants and/or tissue)**<br>• Give the name of the institutional review board or ethics committee that approved the study<br>• Include the approval number and/or a statement indicating approval of this research<br>• Indicate the form of consent obtained (written/oral) or the reason that consent was not obtained (e.g. the data were analyzed anonymously)<br><br>**Animal Research (involving vertebrate animals, embryos or tissues)**<br>• Provide the name of the Institutional Animal Care and Use Committee (IACUC) or other relevant ethics board that reviewed the study protocol, and indicate whether they approved this research or granted a formal waiver of ethical approval<br>• Include an approval number if one was obtained<br>• If the study involved *non-human primates*, add *additional details* about animal welfare and steps taken to ameliorate suffering<br>• If anesthesia, euthanasia, or any kind of animal sacrifice is part of the study, include briefly which substances and/or methods were applied<br><br>**Field Research**<br><br>Include the following details if this study involves the collection of plant, animal, or other materials from a natural setting:<br>• Field permit number<br>• Name of the institution or relevant body that granted permission	
**Data Availability**<br><br>Authors are required to make all data underlying the findings described fully available, without restriction, and from the time of publication. PLOS allows rare exceptions to address legal and ethical concerns. See the PLOS Data Policy and FAQ for detailed information.	Yes - all data are fully available without restriction

A Data Availability Statement describing where the data can be found is required at submission. Your answers to this question constitute the Data Availability Statement and **will be published in the article**, if accepted.<br><br>**Important:** Stating 'data available on request from the author' is not sufficient. If your data are only available upon request, select 'No' for the first question and explain your exceptional situation in the text box.<br><br>Do the authors confirm that all data underlying the findings described in their manuscript are fully available without restriction?	
**Describe where the data may be found in full sentences. If you are copying our sample text, replace any instances of XXX with the appropriate details.**<br><br>• If the data are **held or will be held in a public repository**, include URLs, accession numbers or DOIs. If this information will only be available after acceptance, indicate this by ticking the box below. For example: *All XXX files are available from the XXX database (accession number(s) XXX, XXX.*).<br>• If the data are all contained **within the manuscript and/or Supporting Information files**, enter the following: *All relevant data are within the manuscript and its Supporting Information files.*<br>• If neither of these applies but you are able to provide **details of access elsewhere**, with or without limitations, please do so. For example:<br><br>*Data cannot be shared publicly because of [XXX]. Data are available from the XXX Institutional Data Access / Ethics Committee (contact via XXX) for researchers who meet the criteria for access to confidential data.*<br><br>*The data underlying the results presented in the study are available from (include the name of the third party*	All detected whistle counts obtained from PAMGuard from the closest buoy at 1-second resolution are available via Dryad data repository (accession number: https://doi.org/10.5061/dryad.d2547d88r)

Powered by Editorial Manager® and ProduXion Manager® from Aries Systems Corporation

and contact information or URL).<br>• This text is appropriate if the data are owned by a third party and authors do not have permission to share the data.<br><br>* typeset	
Additional data availability information:	

Manuscript Click here to access/download;Manuscript;Manuscript.docx

Common dolphin whistle responses to experimental mid-frequency sonar

Caroline Casey[a,b] ¶, Selene Fregosi[a]¶, Julie N. Oswald[c], Vincent M. Janik[c], Fleur Visser[d,e], Brandon Southall[a,b]

[a]Southall Environmental Associates, Inc., Aptos, California, United States of America

[b]Institute of Marine Sciences, University of California Santa Cruz, Santa Cruz, California, United States of America

[c]Sea Mammal Research Unit, Scottish Oceans Institute, School of Biology, University of St. Andrews, St. Andrews, United Kingdom

[d]Kelp Marine Research, Hoorn, the Netherlands

[e]Department of Coastal Systems, Royal Netherlands Institute for Sea Research, Den Burg, Texel, the Netherlands

*Corresponding author

E-mail: cbcasey@ucsc.edu

¶CC and SF are Joint Senior Authors of this work.

Abstract

Oceanic delphinids around naval operations are regularly exposed to intense military sonar broadcast within the frequency range of their hearing. However, empirically measuring the impact of sonar on the behavior of highly social, free-ranging dolphins is challenging. Additionally, baseline variability or the frequency of vocal state-switching among social oceanic dolphins during undisturbed conditions is lacking, making it difficult to attribute changes in vocal behavior to anthropogenic disturbance. Using a network of drifting acoustic buoys in controlled exposure experiments, we investigated the effects of mid-frequency (3-4 kHz) active sonar (MFAS) on whistle production in short-beaked (*Delphinus delphis delphis*) and long-beaked common dolphins (*Delphinus delphis bairdii*) in southern California. Given the complexity of acoustic behavior exhibited by these group-living animals, we conducted our response analysis over varying temporal windows (10 min – 5 s) to describe both longer-term and instantaneous changes in sound production. We found that common dolphins exhibited acute and pronounced changes in whistle rate in the 5 s following exposure to simulated Navy MFAS. This response was sustained throughout sequential MFAS exposures within experiments simulating operational conditions, suggesting that dolphins may not habituate to this disturbance. These results indicate that common dolphins exhibit brief yet clearly detectable acoustic responses to MFAS. They also highlight how variable temporal analysis windows – tuned to key aspects of baseline vocal behavior as well as experimental parameters related to MFAS exposure – enable the detection of behavioral responses. We suggest future work with oceanic delphinids explore baseline vocal rates a-priori and use information on the rate of change in vocal behavior to inform the analysis time window over which behavioral responses are measured.

Introduction

Sound production and reception play a critical role in the lives of cetaceans, aiding in important life-history events, including maintenance of social relationships, coordination of group movement, foraging, and evasion of predators [1]. Consequently, substantial effort has been directed toward describing cetacean acoustic behavior [2,3] and evaluating how it is impacted by human-generated disturbance [4-8]. Many sources of anthropogenic noise pollution (*e.g.*, vessel noise, oil and gas exploration, renewable energy, coastal construction and maintenance, fisheries and aquaculture, and military activity) can have varying short and long-term impacts on marine mammal behavior and health [4,9-11]. Concentrated research efforts to characterize these impacts have led to the systematic development of acoustic exposure criteria, informing and improving effective management strategies for regulators and industries (for reviews on auditory criteria, see 12). Such assessments have also highlighted species and noise exposure contexts for which information is sparse or unavailable.

Among cetaceans, oceanic delphinids represent an essential and logistically challenging group to evaluate the impact of anthropogenic noise on vocal behavior. These animals are highly soniferous, abundant, and often extremely gregarious (pods of > 500 individuals are common for some species). Sound production has been demonstrated to play a vital role in the maintenance of social relationships and cohesion among group members [2,3]. Oceanic delphinids are ubiquitous around some U.S. Navy operational areas where mid-frequency active sonar (MFAS; 1-10 kHz) is commonly used for submarine detection in training exercises, resulting in associated large numbers of sonar exposures for these federally protected species. Some of the most powerful MFAS systems (*e.g.*, AN/SQS-53C) emit repeated pings with fundamental

frequencies in the 3-4 kHz range, ping lengths of approximately 1-3 s, and nominal source levels as high as 235 dB re 1 μPa at 1 m root-mean-square (RMS) that may be transmitted for several minutes to hours at high duty cycles (more than 1 ping/min) [13]. Aside from the elevated background noise and potential disturbance that may result from these training exercises [*e.g.*, 14], MFAS signals overlap with the frequencies that oceanic dolphins commonly rely on for social sound (whistle) production. Delphinid whistles are narrowband tonal sounds with most acoustic energy concentrated below 20 kHz [15].

While MFAS has been linked to mass stranding events of cetaceans [13,16] and its effect on cetaceans has been experimentally evaluated in a handful of species (for a recent review, see [17]) , the impact of sonar on the acoustic behavior of oceanic delphinids ~~have~~ not been systematically explored. This is mainly due to the logistical challenges of applying previously developed methods used in other behavioral response studies of individual animals to large aggregations of dolphins. Much of the prior research on cetacean behavioral responses to noise has capitalized on using suction-cup-attached motion-sensing and acoustic recording tags to characterize responses following controlled exposure to MFAS [*e.g.*, 18-20]. Unfortunately, such tags are challenging to deploy and are easily shed by small dolphins due to the tag size relative to the smaller body surface, high drag due to fast swimming speeds, and frequent physical social contact. Additionally, oceanic delphinids commonly occur in large groups that display remarkable coordination, making the collective vocal behavior of the group perhaps a more appropriate focus of analysis [21]. Opportunistic passive acoustic studies relying on large, cabled hydrophone arrays have been used to quantify changes in vocal activity and thus act as a proxy for the presence or absence of multiple animals before, during, and after exposure to MFAS

[22,23). Such experiments are valuable but require extensive, high-cost moored hydrophone arrays with restricted spatial coverage.

Previous studies on acoustic responses of oceanic dolphins to Navy sonar have observed shifting specific frequency components of whistle contours, increasing or decreasing calling rate, increasing call amplitude, and even mimicry of MFAS elements [14, 23-25]. For example, tagged killer whales (*Orcinus orca*) adjust the high-frequency component of their whistles during sonar exposure and increase the number and amplitude of their calls following each ping [24]. False killer whales *(Pseudorca crassidens)* appear to increase their whistle rate and produce more MFAS-like whistles after exposure to simulated sonar [25].

One opportunistic study provided initial insights into the behavioral responses of some social oceanic delphinids to MFAS. Bottlenose dolphins (*Tursiops truncatus*), common dolphins (*Delphinus sp.*), Pacific white-sided dolphins (*Lagenorhynchus obliquidens*), and Risso's dolphins (*Grampus griseus*) incidentally exposed to MFAS showed a cessation of vocalizations, an increase in the intensity of vocalizations, or a combination of both [14]. Of all delphinid species, common dolphins displayed the broadest range of responses, including changing their behavioral state or direction of travel when sonar stopped, increasing the intensity of vocalizations when sonar began, vocalizing very little or not at all during sonar exposure, or a combination of these observations [14]. These results are consistent with a more recent opportunistic evaluation of delphinid responses to an underwater detonation, which showed that whistle rate, complexity, and frequency content varied in response to the explosive event [26]. Depending upon the frequency, intensity, and consistency of these noise exposures, such

behavioral changes could result in physiological consequences that impact overall population health [27]. Unfortunately, information on baseline variability and the frequency of vocal state-switching during undisturbed conditions is lacking among free-ranging, social, oceanic delphinids, making it challenging to interpret the responses observed. While opportunistic studies of delphinid acoustic responses to sonar are insightful, a detailed assessment under controlled experimental conditions is needed to understand the extent to which MFAS impacts oceanic delphinids.

Quantifying vocal behavior in these taxa is complicated by the fact that dolphin acoustic behavior is dynamic, variable, and influenced by a myriad of social and environmental factors [28]. Acoustic behavior and how it changes in response to disturbance must be measured across some predetermined time interval. In previous cetacean behavioral response studies using a conventional controlled exposure experimental (CEE) design, this temporal window was often dictated by logistical limitations of the technology being used (*e.g.,* battery power of tags, the feasibility of continuous behavioral observations, etc.), or designed to match the duration of anthropogenic noise source being evaluated. However, averaging vocal behavior over long time windows may result in missing instantaneous or shorter duration responses at the onset of exposure or at scales more biologically meaningful to the individuals exposed. One way to address this is to examine acoustic metrics computed over various time windows to determine if and when we can attribute a change in vocal behavior to a known, controlled disturbance.

For this study, different broad and fine-scale analytical approaches were used to investigate the effects of experimental MFAS on whistle production in short-beaked (*Delphinus delphis*

delphis) and long-beaked common dolphins (*Delphinus delphis bairdii*) in southern California. By assessing group-level vocal behavior across different time scales, we aimed to:

1. Describe the variability in baseline vocal behavior of common dolphin aggregations during control conditions.
2. Compare vocal responses detected during a controlled exposure to MFAS across broad and fine temporal scales.

Given the need to establish sampling regimes that can be applied and compared across studies, our objective is to provide an informative framework for assessing the complex acoustic behavior exhibited by group-living species. We highlight how using different-sized temporal windows – tuned to key aspects of baseline vocal behavior as well as experimental parameters related to MFAS exposure – impacts the detection of behavioral responses.

Materials and methods

CEEs were conducted with two subspecies of common dolphins. This study was part of a broader effort to quantify group-level responsiveness of oceanic delphinids to military sonar using CEEs around Santa Catalina Island, located off the coast of southern California, USA. Since they regularly occur in mixed groups, we pooled data for the two subspecies to describe baseline vocal data and included subspecies as a potential explanatory variable in our models. The project integrated multiple data streams, including shore-based tracking of dolphin pods, passive acoustics to record vocal activity, and photogrammetry to measure fine-scale behavior [29]. This work was conducted between 2017-2021. We chose our study area because it lies near the Southern California Offshore Range (SCORE) – a tactical training area for the U.S. Navy

Pacific Fleet located off the west side of San Clemente Island – where animals regularly encounter the types of signals we used in our experiment.

CEEs comprised three discrete phases: pre-exposure (baseline), exposure using intermittent simulated MFAS signals, and post-exposure. In control trials, the equipment was lowered into the water from the exposure boat, but no MFAS signals were broadcast. For details about the experimental source and sound source characteristics – including calculations of received levels – see Durban et al. 2022 [29]. Each experimental phase was 10 min in duration. During exposure phases, MFAS 'pings' of 1.6 s in duration consisting of three tonal and frequency-modulated elements between 3.5-4 kHz were transmitted. Pings were emitted at a broadband source level of 212 dB re ~~1μPa~~ RMS every 25 s, which is similar in repetition rate, duty cycle, and the absence of a ramped-up source level (as used in some previous MFAS CEEs) to certain active Navy MFAS systems (*e.g.*, helicopter-dipped sonar systems). Up to 24 total pings were emitted per 10-min exposure phase, provided no permit-mandated shutdowns were implemented for animals within 200 m of the active sound source (this happened in only one CEE). The sound source was positioned relative to focal animal groups using sound propagation modeling to ensure received levels at focal animals were no greater than 140-160 dB re 1μPa RMS.

For every CEE, subspecies identity (based on differences in genetics, morphology, and pigmentation) was determined using aerial images obtained from drone footage, genetic sequencing from biopsy samples, and visual observation. Additionally, group size was characterized by experienced shore-based observers using binoculars or a binocular scope

located at elevated locations (~70 m), enabling a broad overview of the research area (up to 20 km from shore). Shore-based tracking of animals could be conducted for groups up to 7 km from shore. Focal follows included estimating low, best, and high group size, the number of subgroups (defined as all individuals closer to each other than other individuals in the area), the range of inter-individual spacings within subgroups, and distances between subgroups. These observations were taken continuously throughout the experiment at 2-min intervals. For a complete description of these methods, see [29,30]

Acoustic data collection and processing

Passive acoustic monitoring

Passive acoustic recordings were obtained from each target group of dolphins using three drifting, remote-deployed acoustic recording units. Up to three separate recording units were tactically positioned and recovered from a single small (~6 m) rigid-hull inflatable boat, with the objective of placing one recording unit within 500 m of the predicted trajectory of the dolphins during each CEE phase (Fig 1). Each recording unit consisted of a surface buoy and flag with an underwater recorder. The recorder was either a SoundTrap ST300 (Ocean Instruments NZ, Auckland, New Zealand) or a SNAP Recorder (Loggerhead Instruments, Sarasota, FL, USA). Both recorded via a single omnidirectional calibrated hydrophone (SoundTrap: integrated hydrophone, frequency response 0.02–60 kHz ± 3 dB re 1μPa, end-to-end sensitivity -178 dB re 1μPa/V; SNAP: HTI-96-MIN hydrophone, frequency response 1.0-20 kHz ± 3 dB re 1μPa, end-to-end sensitivity -164 dB re 1μPa/V,) which was suspended by a shock-mounted cable at a depth of 10 m. All recording units had a Global Positional System (GPS) tracking device (Trace, SPOT LLC, Chantilly, VA, USA) that recorded the location of the instrument once every min

(Fig 1). Five-min WAV files were continuously recorded at a 96 kHz sampling rate with a 16-bit resolution (SoundTrap) or a 44.1 kHz sampling rate with a 16-bit resolution (SNAP).

Fig 1. A schematic representation of the placement of acoustic recorders (see inset for floating acoustic recording unit) relative to the track of the focal group of dolphins. A single buoy and associated hydrophone were placed within 500 m of the animals during the pre-exposure, exposure, and post-exposure period. The sound source is approximately 1 km from the dolphins at the onset of the exposure period. Note that the source vessel was idling in neutral and was not moving throughout the duration of the exposure period. The dashed line with associated arrows represents the movement path of the focal group. The shore station monitoring the group was positioned on land and is denoted by the theodolite symbol. The map was inspired by images from the NASA Earth Observatory (public domain) and was not drawn to scale (for illustrative purposes only).

Given the dolphins' frequently unpredictable course, the relative proximity of each hydrophone to the animals was determined post-hoc to evaluate which PAM recording unit was closest to the focal group. The animals’ location was known from an associated octocopter drone flight (APO-42, Aerial Imaging Solutions) that utilized a micro 4/3 digital camera (Olympus E-PM2) and 25 mm lens (Olympus M. Zuiko F1.8) mounted to a gimbal. The octocopter flew at approximately 60 m directly above the dolphins to provide sufficient pixel resolution while decreasing the potential for disturbance [see 29 for details]. The relative distances (in meters) between the focal group (from the drone’s GPS) and each recorder (from their flag-mounted GPS units) were estimated for every min of the 30-min experiment using the Haversine formula and

linear interpolation in a custom MATLAB script (Mathworks, Natick, MA, USA, Fig 2). Recordings from the buoy closest to the focal group at 1-min intervals were used for all subsequent analyses. Any recordings made when a recorder exceeded 1.6 km from the focal group (even if the recorder was the closest available) were excluded. This threshold was selected based on a previous assessment of detection ranges of playbacks of odontocete whistles (10-20 kHz) by bottom-mounted hydrophones in southern California, which demonstrated a 95% probability of detection of a 135 dB re 1 μPa dolphin whistle at 1.6 km with an SNR of 2.2 dB re 1μPa [31]. This assessment was supported by the drop-off in whistle amplitude observed in the spectrograms (*post-hoc*) when any buoy surpassed 1.6-1.8 km distance from the focal group (Fig 2). After accounting for this distance cut-off, 9.25 total hours of recordings remained and were used in subsequent analysis.

Fig 2. Spectrograms of each of the three recorders strategically placed on the track line of a moving group of dolphins and associated whistle detections from the buoy closest to the animals at each minute. The solid white line represents the distance between the recorder and the drone centered over the focal group (units on the right y-axis), the red dashed vertical lines denote the experimental period, and the horizontal white dashed line marks the 1.6 km threshold. Estimates of the relative distance between the focal group and each recorder were assessed every min of the 30-min experiment. The bottom panel shows the number of whistles detected on the closest recorder using the PAMGuard Whistle and Moan Detector. The blue dashed lines and associated blue numbers indicate times when the closest buoy switched, and which buoy was closest. The grey area denotes where whistle detections were excluded due to the recorder distance exceeding 1.6 km.

Quantifying whistle production and variability

While common dolphins are known to emit buzzes, echolocation clicks, and whistles, we focused our efforts on characterizing whistle production since they are the critical signal for long-distance communication and play a significant role in group cohesion and coordination [2]. Extraction of whole whistle contours in high background noise with overlapping whistles is exceptionally challenging and results in high rates of missed detections, irrespective of methodology. To create a dataset in which error rates were kept constant across different experimental phases, we used the Whistle and Moan Detector (WMD) module in PAMGuard (v 2.01.05)[32]. The WMD deals with uncertainties by only detecting parts of whistles that clearly stand out above noise using standardized settings across extractions. It is important to note that this often leads to a fragmentation of whistles, with one whistle being split into several independent sections. Thus, whistle detections reported here are not comparable to ones obtained with whole whistle extraction in other studies. However, for our assessment of changes in vocal activity between different experimental phases, it was more important to keep error rates constant to allow for relative comparisons. A qualitative assessment of detector performance within each CEE ensured that variation in whistle detections accurately reflected variation in whistle activity observed in the spectrograms.

The WMD operates on the spectrogram output of the PAMGuard Fast Fourier Transform (FFT) Engine module. We optimized settings for the FFT Engine to provide comparable frequency and temporal resolution of the calculated spectrograms across the two recorders and sampling rates. For the SoundTrap recorders, which had a sampling rate of 96 kHz, the FFT

Engine module computed spectrograms with an FFT length of 1024, hop size of 512, and a Hann window. This resulted in a frequency resolution of 93.75 Hz and time resolution of 10.67 ms. For the SNAP recorders, which had a sampling rate of 44.1 kHz, spectrograms were calculated with a Hann window, FFT length 512, and hop size 256, resulting in a frequency resolution of 86.13 Hz and time resolution of 11.61 ms. The WMD was set to detect whistles between 5 kHz and 20 kHz to exclude detection of the tonal sounds from the simulated mid-frequency sonar source (below 5 kHz) and to standardize the upper detection limit across the two sampling rates and avoid any possible edge effects near the Nyquist frequency of the lower sampling rate. The detection threshold was set at 6.0 dB re 1µPa. Full WMD settings are in the S1 Fig. While the fundamental sonar tonals were excluded by the 5 kHz high-pass cut-off for detections, the high source level of the simulated MFAS resulted in the presence of harmonics in some of the recordings. All harmonics were manually annotated in PAMGuard Viewer using the Spectrogram Annotation module for later removal.

Detected whistles were automatically exported from the PAMGuard detection database using PAMGuard MATLAB tools (https://github.com/PAMGuard/PAMGuardMatlab). Annotated MFAS harmonics were removed using R package `PAMmisc` in R version 4.3.1 [33,34]. Whistles were quantified at 1-s resolution; because whistles were often longer than 1 s in duration, the total number of whistles starting within a 1-s bin was counted, providing a metric for whistle activity as whistles detections per second. For brevity, this is referred to as whistle count throughout the remainder of this manuscript.

Characterization of baseline vocal behavior

Overall whistle count

We calculated the mean and median whistle count per second for the entire 30-min experiment for each control CEE to assess common dolphin vocal behavior under control conditions. These data were evaluated with reference to group size estimates collected by experienced shore-based observers.

Changepoint analysis

We applied a changepoint analytical approach to the control CEE data collected for both common dolphin subspecies to describe the natural variability in vocal behavior during control conditions. Change point detection is used to pinpoint times when the probability distribution of a time series changes (*i.e.*, vocal state changes). The aim is to identify times when either the mean or variance deviates from the expected trends in the dataset and estimate the number and position of all changepoints. Effectively, this approach detects points in time when a significant change in whistle count occurs. First, a 5-s smoothing window was applied to the raw 1-s whistle count data. Then, changepoints in both mean whistle count and whistle count variance were detected using the ‘changepoint’ package in R version 2.2.4 [34-35]. The “BinSeg” (Binary Segmentation) algorithm was used. This provided the number and locations of all state changes in the mean and the variance of whistle count over the 30-min sampling period.

Assessing the impact of disturbance on vocal behavior

Using broad and fine-scale time windows, we employed a hierarchical approach to characterizing the types of vocal responses that might be detected during controlled exposure to MFAS (Fig 3). All statistical analyses were carried out using R version 4.3.1 [34].

Fig 3. Flowchart of methods implemented to assess changes in common dolphin vocal behavior during controlled exposure to MFAS. Methods include pre-processing of acoustic data and baseline vocal behavior analysis, as well as a hierarchical assessment of disturbance on vocal behavior at three temporal scales.

Difference in changepoints by period across CEEs

We conducted a changepoint analysis on all CEEs (both controls and MFAS) to evaluate whether common dolphins change the frequency of vocal state switching as a result of exposure to MFAS. We used the same general method described above but quantified the number of changepoints in the 10-min pre-exposure and 10-min exposure periods separately. Changepoints were detected for both the mean and variance of the whistle count data. The difference in the number of changepoints between the two periods was calculated, and an unpaired t-test was used to evaluate any significant differences between controls and MFAS experiments.

Characterize the impact of MFAS exposure on whistle count: 10-min time scale

To identify potential broad-scale changes in whistle count in response to simulated MFAS exposure, we pooled and analyzed all CEEs (both controls and MFAS exposures) using a Generalized Linear Mixed Model (GLMM) approach, implemented using R package \`glmmTMB\` [36]. We modeled the absolute difference in median whistle count between the 10-min pre-exposure period and the 10-min exposure period (*periodDiff*) as a function of CEE type (*ceeType*; either control or MFAS), a random identity variable (*ceeNum*), dolphin subspecies

(*subSpecies*), the best estimate of total group size from the shore-based observers (*groupSize*), and the mean distance between the focal group and the closest buoy for the entire CEE (*buoyDistance*). Using the absolute value for difference in median whistle count enabled us to explore the magnitude of a potential response. We modeled the relationship using a negative binomial distribution, which fit the count-type data after the transformation. Our full model was:

periodDiff ~ ceeType + ceeNum + subSspecies+ groupSize + buoyDist

We used backward elimination, ΔAIC, and analysis of variance (ANOVA) to select the best model.

Characterize the impact of MFAS exposure on whistle count: 20-s time scale

To characterize more instantaneous changes in whistle production in response to MFAS exposure, we compared dolphin whistle detections in the 20 s before and 20 s after each ping (n = 24 1-s pings per 10-min experimental period, ~25 s between each ping) for both MFAS experiments and controls. We selected this time window to capture sustained variation in whistling behavior within a single ping cycle without overlap between cycles. Differences between these two sequential time bins were calculated by subtracting the mean whistle count for the first bin from the mean whistle count of the second bin (Fig 4B). The first ping started at time 0, the second at 25 s, and so on. Because no actual pings were present in the control experiments, we calculated the change in whistle count surrounding time points placed at the same time as when actual pings would have occurred during an MFAS CEE.

Fig 4. Example plots of (A) raw whistle detections over time, (B) changes in whistle count between 20-s duration sequential bins, and (C) changes in whistle count between 5-s duration sequential bins for the pre-exposure, exposure, and post-exposure period within one CEE. The dashed vertical red line indicates the onset of exposure, and the sequential gray dashed lines represent each ping within the exposure period. Post-exposure periods were not included in the modeling analysis but are presented here for reference. The grey shaded area denotes where whistle detections were excluded due to the recorder distance exceeding 1.6 km.

Like the analysis at the 10-min time scale, we used a GLMM approach (using R package `glmmTMB`) to identify potential significant differences immediately following pings (*pingChange*) in MFAS experiments compared to controls where no pings were present. In addition to the previous fixed effects included at the 10-min scale (*ceeType, ceeNum, subSpecies, buoyDis, and groupSize*), we also included median whistle count per second for the entire experimental period to account for the varied baseline whistling activity across CEEs (*medWhist*).

$$pingChange \sim ceeType + ceeNum + subspecies + groupSize + buoyDist + medWhist$$

Binned ping change data were generally normally distributed but were zero-inflated, so we used a Gaussian distribution for the primary model and additionally modeled the zero inflation as a function of median whistle count (*medWhist*). We used backward elimination, ΔAIC, and analysis of variance (ANOVA) to select the best model.

Characterize the impact of MFAS exposure on whistle count: 5-s time scale

We repeated the analysis conducted above but over a shorter 5-s time window to investigate potential instantaneous changes immediately following pings (Fig 4C). Like the above analysis, we used a GLMM approach to identify potential significant differences in whistle count changes in the 5 s following compared to the 5 s before each ping (*pingChange*) for MFAS experiments compared to controls. We used the same fixed effects implemented at the 20-s scale (c*eeType, ceeNum, [subSpecies, buoyDis, and groupSize, medWhist)* but also included an autocorrelation structure to this analysis *AR(1)* to account for apparent temporal lag effects in exploratory plots.

$$pingChange \sim ceeType + ceeNum + subSspecies + groupSize + buoyDist + medWhist + AR(1)$$

Like the 20-s scale, binned ping change data were normally distributed and zero-inflated; a Gaussian distribution was used for the conditional model and zero-inflation was modeled as a function of median whistle count (*medWhist).* We used backward elimination, ΔAIC, and ANOVA to select the final model.

CEE-by-CEE analysis

We assessed each playback individually at each time scale to better contextualize the severity and persistence of responses and whether dolphins increased or decreased their whistle behavior following sonar exposure. The methods and results for the CEE-by-CEE analysis can be found in the supplementary materials (S1 File).

Results

Characterization of baseline vocal behavior

Basic description

This analysis includes nine control experiments, each conducted on separate days. Four control experiments were conducted with short-beaked common dolphins, and five were conducted with long-beaked common dolphins (Table 1). This resulted in 270 mins of baseline acoustic data for both subspecies (pooled). The average group size across subspecies was 190 individuals (45-300 animals). The dispersion of animals varied considerably within and between control experiments, including small to large groups (55-300 individuals) in tight to lose organization, joint (in a single group with no subgroups), or spread out over several subgroups (range: 2-6) at tens to several hundreds of meters apart (range: 10-800 m).

Whistles were successfully detected across all control experimental deployments. Mean (SD) and Median (IQR) whistle count per second varied between control CEEs (Table 1). In our assessment of the control experiments, we found the total number of whistles varied between 0.3-4.6 whistles/s. Note that the inherent uncertainty error in our group size assessment for large groups did not allow us to calculate accurate whistle rates/individual (see Table 1 for group size estimates and whistle detections).

Table 1. Summary of each controlled exposure experiment, including controls (no sound emitted) and MFAS (playback of mid-frequency active sonar).

CEE ID	Subspecies	Estimated group size	Type	RL (max)	RL (range)	Median [IQR] whistles per second	Changepoints (mean, pre-exposure)	Changepoints (mean, exposure)	Changepoints (variance, pre-exposure)	Changepoints (ariance, exposure)
2019_01	Db	260	MFAS	147 dB re 1µPa RMS	140-147 dB re 1µPa RMS	6.96 [6.39]	26	39	5	1
2019_02	Dd	350	control	n/a	n/a	4.80 [5.19]	37	29	0	5
2019_04	Db	200	control	n/a	n/a	0.25 [0.77]	2	0	4	2
2019_06	Db	45	control	n/a	n/a	0.32 [1.38]	5	0	4	9
2019_07	Db	300	MFAS	154 dB re 1µPa RMS	150-154 dB re 1µPa RMS	3.11 [3.81]	36	20	3	3
2019_08	Db	250	MFAS	142 dB re 1µPa RMS	131-142 dB re 1µPa RMS	1.50 [3.91]	30	12	4	4
2019_09	Dd	250	control	n/a	n/a	4.21 [5.38]	46	44	2	4
2019_10	Dd	30	MFAS	149 dB re 1µPa RMS	146-149 dB re 1µPa RMS	0.091 [0.38]	0	0	3	7
2021_01	Db	150	control	n/a	n/a	3.14 [4.47]	3	51	1	3
2021_02	Db	200	control	n/a	n/a	4.68 [3.89]	18	29	0	4
2021_03	Dd	150	control	n/a	n/a	0.99 [2.01]	11	0	2	0
2021_04	Db	150	control	n/a	n/a	4.66 [4.20]	27	37	4	2
2021_05	Dd	250	control	n/a	n/a	0.36 [1.87]	0	5	8	4
2021_08	Db	30	MFAS	153 dB re 1µPa RMS	145-153 dB re 1µPa RMS	14.13 [7.85]	50	49	2	4
2021_09	Db	200	MFAS	157 dB re 1µPa RMS	152-157 dB re 1µPa RMS	1.17 [2.17]	3	6	2	2
2021_10	Db	300	MFAS	159 dB re 1µPa RMS	150-159 dB re 1µPa RMS	15.34 [9.16]	42	61	3	2
2021_11	Db	10	MFAS	153 dB re 1µPa RMS	150-153 dB re 1µPa RMS	0.0029 [0.063]	0	0	0	6
2021_12	Dd	150	MFAS	152 dB re 1µPa RMS	149-152 dB re 1µPa RMS	2.85 [3.07]	7	24	4	6
2021_13	Dd	200	MFAS	147 dB re 1µPa RMS	139-147 dB re 1µPa RMS	2.21 [4.73]	2	2	9	4

CEE ID denotes the year and the CEE number. Subspecies abbreviations are Db for *D. d. bairdii* and Dd for *D. d. delphis*, and the estimated group size is taken from shore observations. Received sound levels (RLs) are reported by Durban et al. 2022 [29]. The median and interquartile range (IQR, 25-75th percentiles) are given for the raw whistle detections per second of the entire 30-min CEE. Changepoints were calculated for both the mean whistle count and the variance in whistle count, separately for the pre-exposure and exposure periods. Note that "exposures" in controls were quiet periods compared to sound exposure in MFAS trials.

Baseline Changepoint Analysis

The baseline changepoint analysis revealed that mean detected whistle counts over the 30-min sampling periods changed once every min, and variance in detected whistle counts changed once every 3.5 min.

Assessing the impact of disturbance on whistle behavior

A total of 10 MFAS CEEs were conducted – seven of which included long-beaked common dolphins and three of which included short-beaked common dolphins. The calculated average received level across all experiments was 151 dB re 1μPa RMS (range 142-159 dB re 1μPa RMS, Table 1). The average group size for MFAS CEEs was 173 individuals (range 10-300, Table 1). For the changepoint analysis, all 10 MFAS experiments were included. However, for the assessment of changes in whistle behavior across different time scales, CEE 2021_11 (conducted with long-beaked common dolphins) had to be excluded because the overall lack of detected whistles could not be successfully modeled. Consequently, the modeling results include nine controls and nine MFAS experiments.

Number of changepoints in pre-exposure vs exposure

Changepoint analysis was run for all control (9) and MFAS (10) experiments. The number of changepoints detected in both the mean and variance of whistle count during the pre-exposure and exposure periods is presented in Table 1 for both controls and MFAS experiments. There was no significant increase or decrease in the mean and variance of detected whistle counts following MFAS exposure when compared to the natural variance present during control conditions. The difference (Δ) in change points in variance of whistle detections between the pre-exposure and exposure period did not differ significantly between controls and MFAS CEEs

(controls: M = 3.1, SD = 1.4; MFAS: M = 2.5, SD = 2.3; t(17) = 0.69, p = 0.5). The same was true when comparing the mean whistle count between both experimental types (controls: M = 11.3, SD = 14.2; MFAS, M = 8.7, SD = 8.5; t(17) = 0.5, p = 0.63).

Impact of MFAS exposure on whistle count: 10-min time scale

Whistle detections did not change between the pre-exposure and exposure period during MFAS experiments at the 10-min time scale. The preferred model was the simplest model with the absolute value of the change in median whistle count as a function of only CEE type (either MFAS or control). There was no significant effect of CEE type on the change in median whistle detections (negative binomial GLMM, n = 18, p = 0.8). The full model (ΔAIC 5.4) indicated that no proposed predictor variables (CEE type, CEE number, subspecies, buoy distance, or group size) had a significant effect on the change in median whistle count between the pre-exposure and exposure periods for both MFAS and control CEEs (negative binomial GLMM, n = 19: P > 0.05 for all variables, Table 2).

Table 2. Overview of GLMMs used at three time scales – 10-mins, 20-s, and 5-s.

Conditional model	Zero-inflation model	Distribution	ΔAIC	Degrees of freedom	Dispersion (σ^2)
10-min scale					
abs(*periodDiff*) ~ *ceeType*	n/a	nbinom2	0	3	5.44
abs(*periodDiff*) ~ *ceeType* + (1 | *ceeNum*)	n/a	nbinom2	2	4	1.04
abs(*periodDiff*) ~ *ceeType* + (1 | *ceeNum*) + *subSpecies* + *buoyDist* + *groupSize*	n/a	nbinom2	5.4	7	1.48
20-s scale					
pingDiff ~ ceeType + medWhist	*~medWhist*	gaussian	0	6	7.1
pingDiff ~ ceeType	*~medWhist*	gaussian	5.1	5	7.23
pingDiff ~ ceeType + subSpecies + groupSize + buoyDist + medWhist	*~medWhist*	gaussian	5.3	9	7.09

pingDiff ~ *ceeType* + (1 | *ceeNum*)	~*medWhist*	gaussian	7.1	6	7.23
pingDiff ~ *ceeType*	none	gaussian	28.3	3	6.71
5-s scale					
pingDiff ~ *ceeType* + *groupSize* + *medWhist* + ar1(*times* + 0 | *ceeNum*)	~*medWhist*	gaussian	0	9	10.1
pingDiff ~ *ceeType* + (1 | *ceeNum*) + *subSpecies* + *groupSize* + *buoyDist* + *medWhist* + ar1(*times* + 0 | *ceeNum*)	~*medWhist*	gaussian	2.6	12	10.1
pingDiff ~ *ceeType* + ar1(*times* + 0 | *ceeNum*)	~*medWhist*	gaussian	13.8	7	9.83
pingDiff ~ *ceeType* + (1 | *ceeNum*) + ar1(*times* + 0 | *ceeNum*)	~*medWhist*	gaussian	15.8	8	9.83
pingDiff ~ *ceeType*	none	gaussian	207.8	3	10.9

Model formulas (conditional and zero-inflation if included) are listed for each time scale of analysis, and within each time scale are given in ascending order of ΔAIC relative to the best model (ΔAIC = 0).

Characterize the impact of MFAS exposure on whistle count: 20-s time scale

Whistle detections did not differ significantly over the 20-s time windows surrounding each ping. The preferred model at the 20-s scale included only predictor variables for CEE type and median whistle count (Table 2). The results of this model showed that CEE type did not have a significant effect on changes in whistle count in the 20 s after each ping (GLMM, n=18, slope = 0.47, SE = 0.27, P > 0.05, Table 2) but that the baseline median whistle count for that experimental period was a significant predictor for the change in whistle count following a ping or control treatment (GLMM, n = 19, slope = 0.76, SE = 0.028, p = 0.0075).

Characterize the impact of MFAS exposure on whistle count: 5-s time scale

The preferred model at the 5-s scale included the temporal autocorrelation structure and three explanatory variables, CEE type, group size, and median whistle count, all of which had a significant effect on the change in whistle count in the 5 s immediately following a ping

compared to the 5 s immediately before a ping. When accounting for all other variables, the magnitude of the change in whistle count in the 5 s following an actual MFAS ping was 1.4 times greater than any change in whistle count following control treatments (GLMM, n=18, slope = 1.43, SE = 0.47, p = 0.002, Table 2). The results of the CEE-by-CEE analysis (S1 File) showed that in five of the nine MFAS experiments, detected whistle counts were elevated in the 5 s after each ping for the entire 10-min exposure period (plots of all raw whistles are provided in S2 Fig). The effect was particularly pronounced (outside the 75th percentile; S3 Fig) in the first ping of six of the MFAS CEEs; whistle activity increased in the 5 s following the first MFAS ping up to 15 times the whistle count in the 5 s before the first ping (mean of all MFAS CEEs 3.9, SD 5.2), compared to increases of only up to 1.4 times (mean 0.46, SD 0.88) at the start of control treatments (Fig 5, S3 Fig). Additionally, group size and median whistle count for the exposure period were significant predictor variables. Larger groups showed more extensive changes in whistle count following pings and control treatments (GLMM, n=18, slope = 0.007, SE = 0.003, p = 0.008, Table 2), and when the median background whistle count was higher, so too were the changes following pings and control treatments (GLMM, n=18, slope = 0.25, SE = 0.048, p = < 0.005, Table 2).

Fig 5. (A) Spectrogram example of 5 s before and 5 s after the first ping for MFAS CEE 2021_08 illustrates the significant increase in whistle count immediately following the cessation of the ping. The focal group was comprised of approximately 30 long-beaked common dolphins. The MFAS signal can be seen between 3 and 4 kHz. (B) Boxplot of the change in whistle count from the 5 s before to the 5 s following each of the 24 pings for CEE 2021_08.

Boxplot shows median, 25th, and 75th percentiles, with raw whistle count changes as open gray circles. The change following the first ping is shown as a red star.

Discussion

Multiple factors – including rapid changes in behavioral state throughout the experiment and variation in group size and composition – make it difficult to assess whether changes in vocal behavior are due to disturbance or natural variability. We assessed vocal behavior during control conditions to understand typical acoustic variation among common dolphins. We found that dolphins exhibited natural vocal state changes (identified by the changepoint analysis) in whistle production approximately once every min. This rapid acoustic state switching informed our analytical approach, which utilized a range of temporal windows to test for changes in whistle count (5 s – 10 min) before and after MFAS exposure. Across the longer time windows selected; we did not detect a shift in whistle behavior attributable to MFAS. However, CEE type significantly affected the change in whistle count in the 5 s following a ping compared to the 5 s immediately before a ping.

The initial selection of the time period over which behavior was assessed in response to Navy sonar (10 min) for this project was influenced by multiple factors, including the flight endurance of the drone used for calculating animal distance to our recording buoys, our ability to consistently track large groups of fast-moving dolphins, and other previous BRS studies using MFAS (*e.g.*, 18, 37]. Many of the aforementioned constraints are imposed by the logistics of fieldwork. An informed approach to identifying behavioral responses to anthropogenic sources also requires prior knowledge of the timing of behavioral state switching in the study species.

For example, previous work with blue whales (*Balaenoptera musculus*) evaluated several behavioral metrics (*e.g.*, maximum depth, dive time, ascent/descent rate) in responses to simulated MFAS exposure over a 30-min time window [37]. This exposure duration (which included a 30-min pre-exposure period) was adequate to capture a behavioral change given the typical duration of their dive cycles (5-8 mins) [38]. In contrast, beaked whales exhibit incredibly long, deep foraging dives that often last over an hour, followed by long periods of recovery [*e.g.*, 39,40). Consequently, studies focused on direct measurements of behavioral response by Cuvier's beaked whales to MFAS extended their pre-exposure baseline period up to 9.3 hours and evaluated their response to sonar for up to 1.7 hours after the exposure period [41].

While the 10-min exposure period seemed appropriate given the fast-paced lifestyle of common dolphins, neither the aggregate model nor the individual assessment of each CEE detected a change in acoustic behavior that could be ascribed to sonar exposure at this time scale. Even when we explored variation in whistle production during the 20-s surrounding each ping, the experimental period was not a significant predictor of changes in whistle count in either the MFAS or control experiments, as vocal state switching often occurs within a 40-s time window under baseline conditions. While the impacts of sonar may be evident over the 10-min exposure period for other behavioral metrics (*i.e.*, changes in behavioral state, group composition, diving behavior), our analysis reveals that changes in acoustic behavior are limited to an extremely narrow time window in these two subspecies.

It was only at the 5-s time scale surrounding each ping that we observed dolphins exhibiting an acute acoustic response, which included a rapid increase in whistle production relative to the 5

s immediately before sound exposure. On average, dolphins increased their whistle count four times the average in the 5 s preceding the first ping of the exposure. In one MFAS experiment, dolphins increased their whistle production 15 times compared to the whistle count in the 5 s immediately before the first ping (S3 Fig). This elevated vocal response following the first ping of the exposure was seen in six of the nine MFAS exposures where whistles were present.

Elevations in whistle detections did not occur during the sonar transmission itself – which lasted for 1.6 s. Rather, the increased vocal production occurred once the signal had been transmitted, often abating within ~ 10 s. The lack of whistle production during sonar transmission may be a tactic for reducing acoustic interference and masking, which has been shown to impact the detection, discrimination, and localization of relevant signals [42]. If the signal is predictable (as in our experiment), then animals should be able to adjust the timing of sound production to limit communication to periods in which noise is reduced [*e.g.*, 42-45]. The ability of dolphins to learn the timing of intermittent noise has previously been demonstrated by Finneran et al., 2023 [42], who showed that individuals can modify their hearing sensitivity before the onset of predictably timed impulses, presumably to mitigate adverse auditory impacts. Surprisingly, little is known about their capacity to alter the timing of vocal production in response to interfering signals under baseline conditions.

The sudden increase in vocal behavior following the first ping could be an example of the amplification of the behavior of group members through recruitment or reinforcement (*i.e.*, positive feedback) [46]. In this scenario, one dolphin may whistle in response to a surprising, salient stimuli, and others may follow suit. As this recruitment response continues, the number of

dolphins producing whistles will increase further, and information is spread rapidly throughout the group [47]. In such cases, a few key individuals could catalyze the collective behavior of the rest of the group. While it would be difficult to evaluate this process using acoustic data alone, concurrent video data collected from the associated drone flights is currently being assessed to explore the spatial movement patterns of the group and identify those individuals that successfully initiate changes in group movement. Alternatively, the rapid increase in vocal behavior following each ping could indicate that multiple animals exchange whistles to contact their closest social partners in the presence of an unknown stimulus. This could be expected given the role of whistles in group cohesion and coordination [2,48].

Surprisingly, in the playbacks when dolphins showed a significant increase in vocal behavior in the ~~5-sec~~ following the first ping (6 of 9 CEEs, S3 Fig), the severity of their acoustic response did not abate over the course of the exposure (S2 Fig). This suggests that dolphins did not habituate to successive pings (*i.e.*, show a progressive decrease in the amplitude of a vocal behavioral response after repeated exposure). This observation is surprising given that the dolphins tested in this study likely live a large portion of their lives in areas regularly ensonified by Navy sonar. Continued work is needed to confirm these initial findings; for example, future studies could compare animals' responsiveness in the Southern California Bight to nearby populations in areas where Navy sonar is not regularly present (*e.g.*, Monterey Bay). Considering whether dolphins may be learning to modify their vocal behavior in response to repeated noise exposure may provide foundational evidence to support using vocal rates as a measure of sensitization or habituation to anthropogenic stimuli, as has been done in terrestrial species [as in 49,50].

Future management decisions mitigating the impact of sonar on oceanic delphinids should consider our reported results of clear responses during CEEs when analyses were conducted at the appropriate temporal resolution. Concerning the most recent methodology for assessing the relative response severity for free-ranging marine mammals to acoustic disturbance [51] – had the acoustic response of animals to MFAS been pooled across the 10-min time window – common dolphins likely would have been assigned a behavioral response severity score of 0 (no response detected). However, when evaluating vocal behavior across a shorter time window, common dolphins would be elevated to a category three severity, which includes an increase in possible contact or alarm calls [51]. Ultimately, continued work with this (and other closely related) species should also consider how observed behavioral responses vary with respect to other contextual parameters, including behavioral state, group composition (*e.g.*, presence or absence of calves), seasonality, and environmental covariates. Concurrent efforts from this project aim to integrate passive acoustics with other remotely sensed datasets (*i.e.*, shore-based group tracking and aerial photogrammetry) to identify group-level behavioral changes and quantify exposure-response relationships [49]. Paired with energetic modeling methods [*e.g.*, 27,53,54], these data can link ~~these~~ observed short-term behavioral responses to long-term fitness outcomes in this species and inform effective mitigation strategies.

Our study has some limitations that can be addressed in future work. Given the close phylogenetic relationship between short-beaked and long-beaked common dolphins [55,56], the basic description of baseline vocal behavior (*i.e.*, whistle count data) was combined across subspecies in our analysis. However, recent work by ~~Oswald et al.~~ discovered unique whistle

contours in short-beaked common dolphins suggesting that these distinctive acoustic signals could help facilitate recognition between these *Delphinus* subspecies [57]. An increase in sample size for both long-beaked and short-beaked common dolphins would provide a more detailed understanding of their baseline vocal behavior and enable the exploration of whether each subspecies shows a differential or similar response in whistle-type usage to MFAS. Additionally, photo identification of individuals within these large, ephemeral groups is challenging. Consequently, whether individual dolphins around Catalina Island were exposed more than once to the experimental treatment is unknown. Future research could direct efforts toward other oceanic delphinids with smaller group sizes where photo-identification is feasible and reliable (*e.g.*, bottlenose or rough-toothed dolphins).

In any behavioral experiment, the response of the individual or group that is tested should be measured and interpreted over a time window that is informed by their natural behavior. We suggest that future work with other oceanic delphinid species explore baseline vocal rates a-priori and use information on vocal state-switching to inform the analysis time window over which behavioral responses are measured. Given these animals' susceptibility to frequent MFAS exposure in Navy operational areas, evaluating how repeated exposure influences responses is of particular interest. Future work should continue to explore critical factors likely to affect the probability of response among these large groups, including their behavioral state and proximity to the sound source and received level at the onset of exposure.

Acknowledgments

Animal observations, octocopter flights over dolphins, close approaches, and CEEs were conducted under NMFS permits 19116 and 19091. Additionally, all activities reported in this study were reviewed and approved by the Institutional Animal Care and Use Committee (IACUC Protocol No: CRC-2021-AUP-06, CRC-2021-AUP-08). We thank the *M/V Magician* and Captain Carl Mayhugh for support in the field and the University of Southern California's Wrigley Institute for Environment and Sustainability for their hospitality throughout this project. This project would not have been possible without the dedicated contribution of all members of the Tagless-BRS team. Thank you to Stacy DeRuiter for extensive advice on statistical modeling and analysis. Additionally, we appreciate the thoughtful feedback provided by Ari Friedlaender, Colleen Reichmuth, Peter Cook, and John Durban during analysis and manuscript preparation. We also thank Ryan Jones, who created the line drawings for Fig 1.

References

1. Tyack PL. Acoustic communication under the sea. In: Hopp SL, Owren MJ, Evans CS, editors. Animal acoustic communication. Berlin / Heidelberg: Springer; 1998.

2. Janik VM. Acoustic communication in delphinids. Adv Study Behav. 2009;40: 123–157.

3. Lammers MO, Oswald JN. Analyzing the acoustic communication of dolphins. In: Herzing DL, Johnson CM, editors. Dolphin communication and cognition: Past, present, and future. Cambridge: MIT Press; 2015. pp. 107-130.

4. Erbe C, Dunlop R, Dolman S. Effects of noise on marine mammals. In: Slabbekoorn H, Dooling R, Popper A, Fay R, editors. Effects of anthropogenic noise on animals. New York: Springer; 2018.

5. Williams R, Wright AJ, Ashe E, Blight LK, Bruintjes R, Canessa R, et al. Impacts of anthropogenic noise on marine life: Publication patterns, new discoveries, and future directions in research and management. Ocean Coast Manag. 2015;115: 17–24.

6. Southall BL, Finneran JJ, Reichmuth C, Nachtigall PE, Ketten DR, Bowles AE, et al. Marine mammal noise exposure criteria: Updated scientific recommendations for residual hearing effects. Aquat Mamm. 2019;45(2): 125–232.

7. Hatch LT, Wahle CM, Gedamke J, Harrison J, Laws B, Moore SE, et al. Can you hear me here? Managing acoustic habitat in US waters. Endanger Species Res. 2016;30(1): 171–86.

8. Tyack PL, Janik VM. Effects of noise on acoustic signal production in marine mammals. In: Brumm H, editor. Animal communication and noise. New York: Springer; 2013. pp. 251-271.

9. Nowacek DP, Thorne LH, Johnston DW, Tyack PL. Responses of cetaceans to anthropogenic noise. Mamm Rev. 2007;37(2): 81–115.

10. Hastie G, Merchant ND, Götz T, Russell DJF, Thompson P, Janik VM. Effects of impulsive noise on marine mammals: Investigating range-dependent risk. Ecol Appl. 2019;29: e01906.

11. Harris CM, Sadykova D, DeRuiter SL, Tyack PL, Miller PJO, Kvadsheim PH, et al. Dose response severity functions for acoustic disturbance in cetaceans using recurrent event survival analysis. Ecosphere. 2015;6(11): 1–14.

12. Southall BL, Finneran JJ, Reichmuth C, Nachtigall PE, Ketten DR, Bowles AE, et al. Marine mammal noise exposure criteria: Updated scientific recommendations for residual hearing effects. Aquat Mamm. 2019;45(2): 125–232.

13. D’Amico A, Pittenger R. A brief history of active sonar. Aquat Mamm. 2009;35(4): 426–34.
14. Henderson EE, Smith MH, Gassmann M, Wiggins SM, Douglas AB, Hildebrand JA. Delphinid behavioral responses to incidental mid-frequency active sonar. J Acoust Soc Am. 2014;136(4): 2003–2014.
15. Oswald JN, Rankin S, Barlow J. The effect of recording and analysis bandwidth on acoustic identification of delphinid species. J Acoust Soc Am. 2004;116(5): 3178–3185.
16. Filadelfo R, Mintz J, Michlovich E, D’Amico A, Tyack PL, Ketten DR. Correlating military sonar use with beaked whale mass strandings: What do the historical data show? Aquat Mamm. 2009;35(4): 435–444.
17. Guan S, Brookens T. An overview of research efforts to understand the effects of underwater sound on cetaceans. Vol. 2, Water Biol Secur. 2023;2: 100141.
18. Southall BL, DeRuiter SL, Friedlaender A, Stimpert AK, Goldbogen JA, Hazen E, et al. Behavioral responses of individual blue whales (*Balaenoptera musculus*) to mid-frequency military sonar. J Exp Biol. 2019;222: 5.
19. Sivle LD, Kvadsheim PH, Fahlman A, Lam FPA, Tyack PL, Miller PJO, et al. Changes in dive behavior during naval sonar exposure in killer whales, long-finned pilot whales, and sperm whales. Front Physiol. 2012;11: 400
20. Deruiter SL, Boyd IL, Claridge DE, Clark CW, Gagnon C, Southall BL, et al. Delphinid whistle production and call matching during playback of simulated military sonar. Mar Mamm Sci. 2013;29(2): E46-E59.

21. Ioannou CC, Laskowski KL. A multi-scale review of the dynamics of collective behaviour: from rapid responses to ontogeny and evolution. Philos Trans R Soc Lond, B. 2023;10(378): 20220059.

22. Durbach IN, Harris CM, Martin C, Helble TA, Henderson EE, Ierley G, et al. Changes in the movement and calling behavior of minke whales (*Balaenoptera acutorostrata*) in Response to Navy Training. Front Mar Sci. 2021;8: 660122.

23. Rendell LE, Gordon JCD. Vocal response of long-finned pilot whales (*Globicephala melas*) to military sonar in the Ligurian Sea. Mar Mamm Sci. 1999;15(1): 198–204.

24. Miller PJO, Antunes RN, Wensveen PJ, Samarra FIP, Catarina Alves A, Tyack PL, et al. Dose-response relationships for the onset of avoidance of sonar by free-ranging killer whales. J Acoust Soc Am. 2014;135(2): 975–93.

25. DeRuiter SL, Boyd IL, Claridge DE, Clark CW, Gagnon C, Southall BL, et al. Delphinid whistle production and call matching during playback of simulated military sonar. Mar Mamm Sci. 2013;2: 46-59.

26. Lara G, Bou-Cabo M, Llorens S, Miralles R, Espinosa V. Acoustical behavior of delphinid whistles in the presence of an underwater explosion event in the Mediterranean coastal waters of spain. J Mar Sci. 2023;11(4): 780.

27. Keen KA, Beltran RS, Pirotta E, Costa DP. Emerging themes in population consequences of disturbance models. Proc R Soc B. 2021;288(1957): 20210325.

28. King SL, Connor RC, Montgomery SH. Social and vocal complexity in bottlenose dolphins. Trends Neursci. 2022; 45: 881-883.

29. Durban JW, Southall BL, Calambokidis J, Casey C, Fearnbach H, Joyce TW, et al. Integrating remote sensing methods during controlled exposure experiments to quantify group responses of dolphins to navy sonar. Mar Pollut Bull. 2022;174: 113194.

30. Visser, F. Moving in concert: Social and migratory behaviour of dolphins and whales in the North Atlantic Ocean. PhD dissertation, University of Amsterdam. 2014.

31. Hager CA. Assessment of the performance of the near-bottom hydrophones of the US Navy Southern California offshore range in detecting, localizing and reconstructing 10-20KHZ odontocete whistles. PhD dissertation, Naval Postgraduate School. 2008.

32. Gillespie D, Mellinger DK, Gordon J, McLaren D, Redmond P, McHugh R, et al. PAMGUARD: Semiautomated, open-source software for real- time acoustic detection and localization of cetaceans. J Acoust Soc Am. 2009;125(4): 2547–2547.

33. Sakai T, Barlow, J, Griffiths, E, Oswald, J, Schmidt, V.PAMmisc: Miscellaneous functions for passive acoustic analysis. 2023. https://CRAN.R-project.org/package=PAMmisc

34. R Core Team. R: A Language and Environment for Statistical Computing. R Foundation for Statistical Computing, Vienna, Austria. 2023. https://www.R-project.org/

35. Killick R, Eckley I. changepoint: An R package for changepoint analysis. J Stat Softw. 2014;58(3): 1-9.

36. Brooks ME, Kristensen K, Van Benthem KJ, Magnusson A, Berg CW, Nielsen A, et al. glmmTMB balances speed and flexibility among packages for zero-inflated generalized linear mixed modeling. The R Journal. 2017;9(2): 378-400.

37. Goldbogen JA, Southall BL, DeRuiter SL, Calambokidis J, Friedlaender AS, Hazen EL, et al. Blue whales respond to simulated mid-frequency military sonar. Proc R Soc B. 2013;280: 20130657.

38. Croll DA, Acevedo-Gutierrez A, Tershy BR, Urban-Ramırez J. The diving behavior of blue and fin whales: Is dive duration shorter than expected based on oxygen stores? Comp Biochem Physiol, Part A Mol Integ. 2001;129(4): 797-809.

39. Baird RW, Webster DL, Schorr GS, McSweeney DJ, Barlow J. Diel variation in beaked whale diving behavior. Mar Mamm Sci. 2008;24(3): 630–642.

40. Shearer JM, Quick NJ, Cioffi WR, Baird RW, Webster D, Foley HJ, et al. Diving behaviour of Cuvier's beaked whales (*Ziphius cavirostris*) off Cape Hatteras, North Carolina. R Soc Open Sci. 2019;6(2): 181728.

41. Deruiter SL, Southall BL, Calambokidis J, Zimmer WMX, Sadykova D, Falcone EA, et al. First direct measurements of behavioural responses by Cuvier's beaked whales to mid-frequency active sonar. Bio Lett. 2013;9: 20130223.

42. Finneran JJ, Schlundt CE, Bowman V, Jenkins K. Dolphins reduce hearing sensitivity in anticipation of repetitive impulsive noise exposures. J Acoust Soc Am. 2023;153(6): 3372.

43. Egnor SR, Wickelgren JG, Hauser MD. Tracking silence: adjusting vocal production to avoid acoustic interference. J Comp Physiol. 2007;193: 477-483.

44. Kiai A, Clemens J, Kössl M, Poeppel D, Hechavarría J. Flexible control of vocal timing in bats enables escape from acoustic interference. bioRxiv. 2023: 2023-05.

45. Munoz-Santos I, Ríos-Chelén AA. Vermilion flycatchers avoid singing during sudden peaks of anthropogenic noise. acta ethologica. 2022;26(3): 201-210.

46. Bonabeau E, Theraulaz G, Deneubourg J.L, Aron S, Camazine S. Self-organization in social insects. Trends Ecol Evol. 1997;12(188).

47. Sumpter DJT. The principles of collective animal behaviour. Philos Trans R Soc Lond, B. 2005;361: 5–22.

48. Caldwell MC, Caldwell DK. Vocalization of naive captive dolphins in small groups. Science. 1968;159(3819): 1121-1123.

49. Francis CD, Ortega CP, Cruz A. Noise pollution changes avian communities and species interactions. Curr Biol. 2009;19(16):1415–1419.

50. Brown CL, Hardy AR, Barber JR, Fristrup KM, Crooks KR, Angeloni LM. The effect of human activities and their associated noise on ungulate behavior. PloS One. 2012;10;(7): e40505.

51. Southall BL, Tollit D, Amaral J, Clark CW, Ellison WT. Managing human activity and marine mammals: A biologically based, relativistic risk assessment framework. Front Mar Sci. 2023;10.

52. Harris CM, Thomas L, Falcone EA, Hildebrand J, Houser D, Kvadsheim PH, et al. Marine mammals and sonar: Dose- response studies, the risk- disturbance hypothesis and the role of exposure context. J Appl Ecol. 2018;55:396-404.

53. Harwood J, King S, Booth C, Donovan C, Schick RS, Thomas L, et al. Understanding the population consequences of acoustic disturbance for marine mammals. In Popper AN, Hawkins A, editors. The effects of noise on aquatic life II. New York: Springer; 2016. pp. 417-423.

54. McHuron EA, Adamczak S, Costa DP, Booth C. Estimating reproductive costs in marine mammal bioenergetic models: A review of current knowledge and data availability. Conserv Physiol. 2023;11(1): coac080.

55. Natoli A, Cañadas A, Peddemors VM, Aguilar A, Vaquero C, Fernandez-Piqueras P, et al. Phylogeography and alpha taxonomy of the common dolphin (*Delphinus* sp.). J Evol Biol. 2006;19(3): 943-954.

56. McGowen MR, Tsagkogeorga G, Álvarez-Carretero S, Dos Reis M, Truebig M, Deaville R, et al. Phylogenomic resolution of the cetacean tree of life using target sequence capture. Syst Biol. 2020;69(3): 479-501.

57. Oswald JN, Walmsley SF, Casey C, Fregosi S, Southall BL, Janik VM. Species information in whistle frequency modulation patterns of common dolphins. Phil Trans R Soc B. 2021;376: 20210046.

Supporting information

S1 Fig. PAMGuard Whistle Detection Parameters. Detection settings for the PAMGuard Whistle and Moan Detector.

S1 File. CEE-by-CEE Analysis. The main manuscript presents methods and results for analyzing common dolphin vocal response to simulated mid-frequency sonar, pooling data from all CEEs. We acknowledge that some readers may be interested in additional information about each of the 19 CEEs (10 MFAS exposures and nine controls), so we have included additional methods and results for each CEE.

S2 Fig. Plots of raw per-second whistle detections for all included CEEs. CEE-ID (year and number) is given in the bottom right of each page. The red dashed line indicates the start of the exposure period, with dashed grey lines indicating the timing of each ping (in an MFAS exposure) or control treatment. The solid gray vertical line indicates the start of the post-exposure period.

S3 Fig. Boxplots of the change in whistle count from the 5 s before to the 5 s following each of the 24 pings for all CEEs (MFAS and controls). Boxplot shows median, 25th, and 75th

percentiles, with raw whistle count changes as open gray circles. The change following the first ping is shown as a red star.

Click here to access/download;Figure;Fig 1.tif

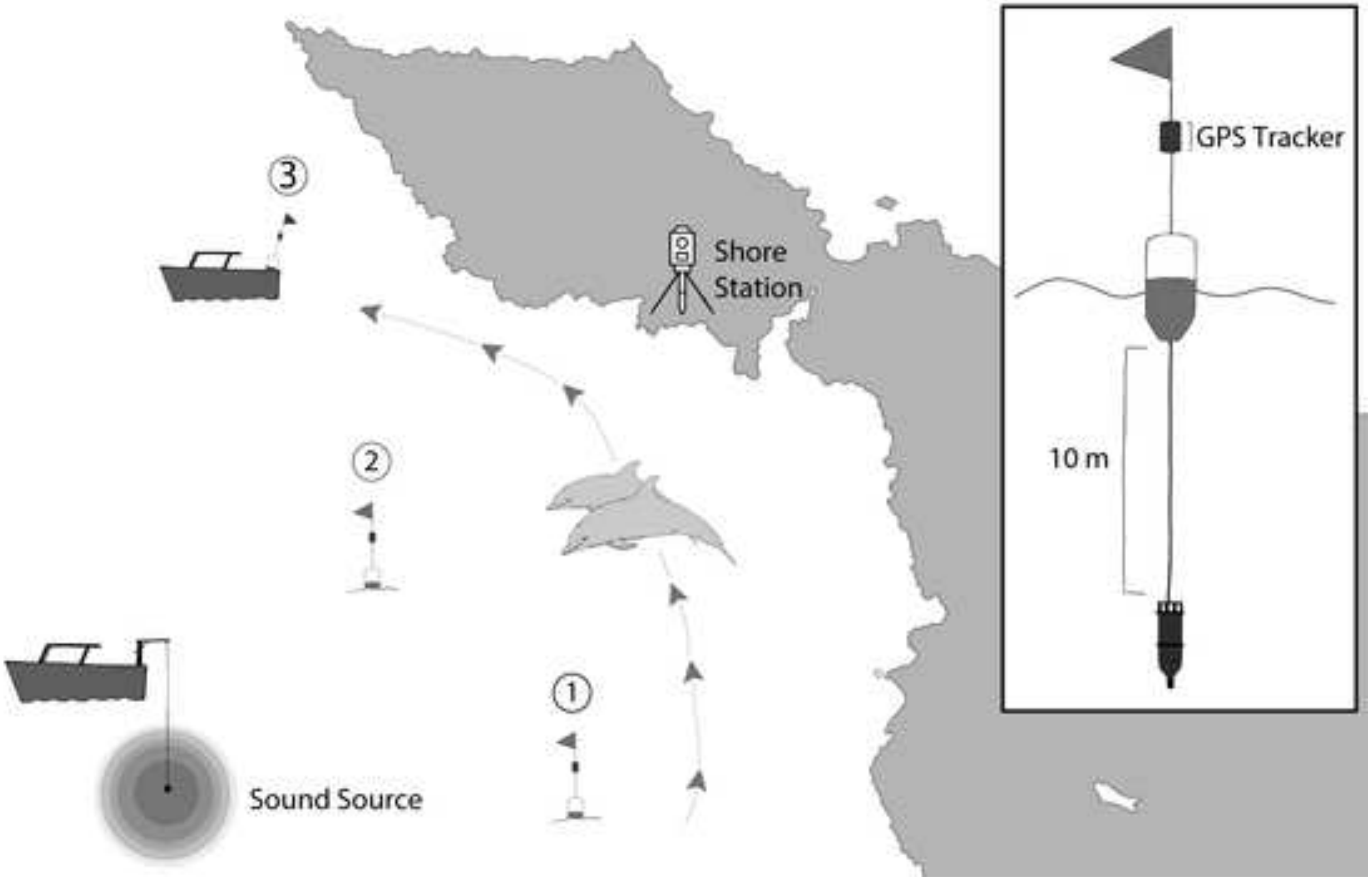

Figure
Click here to access/download;Figure;Fig 2.tif

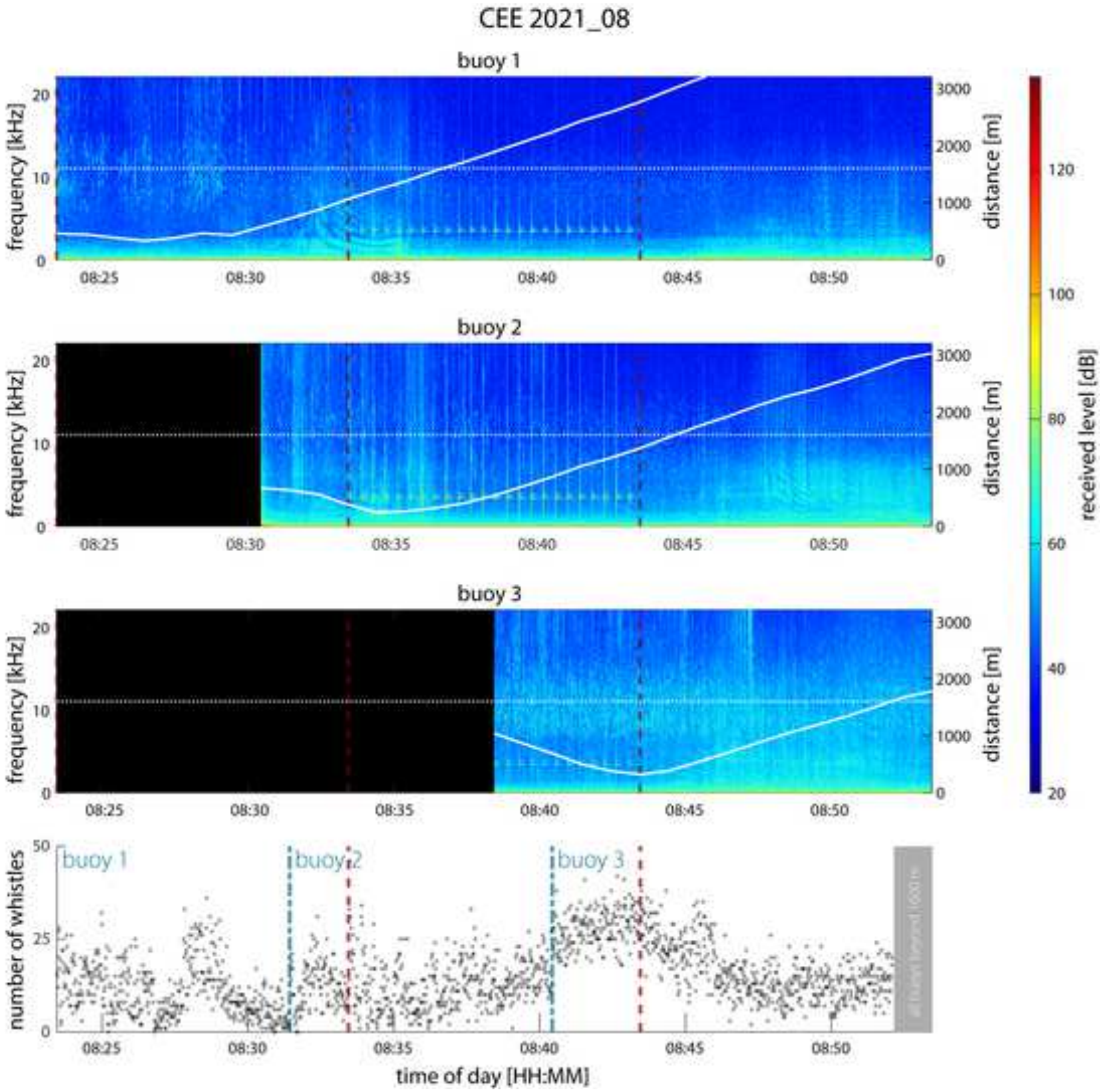

 Click here to access/download;Figure;Fig 3.tif

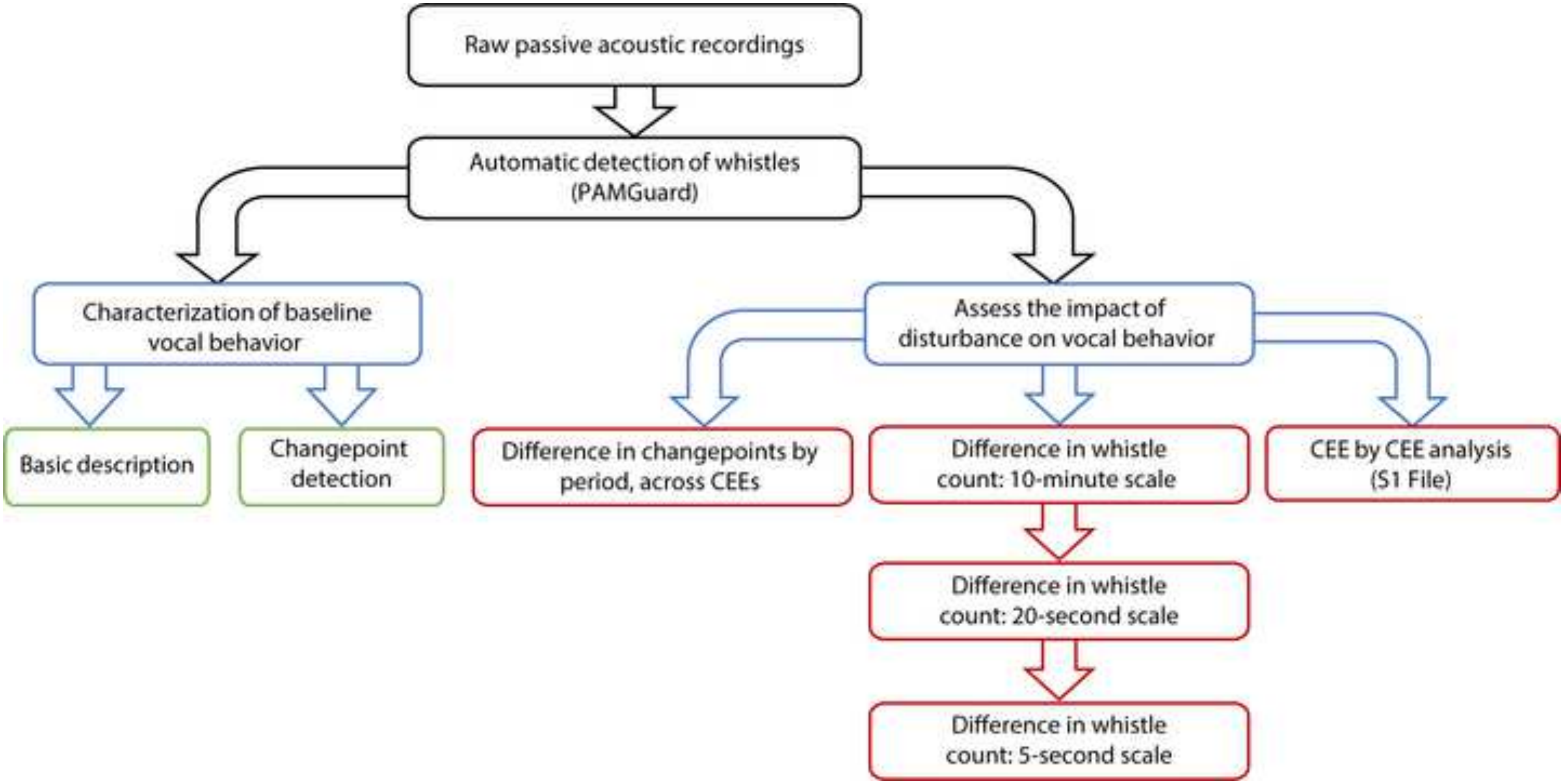

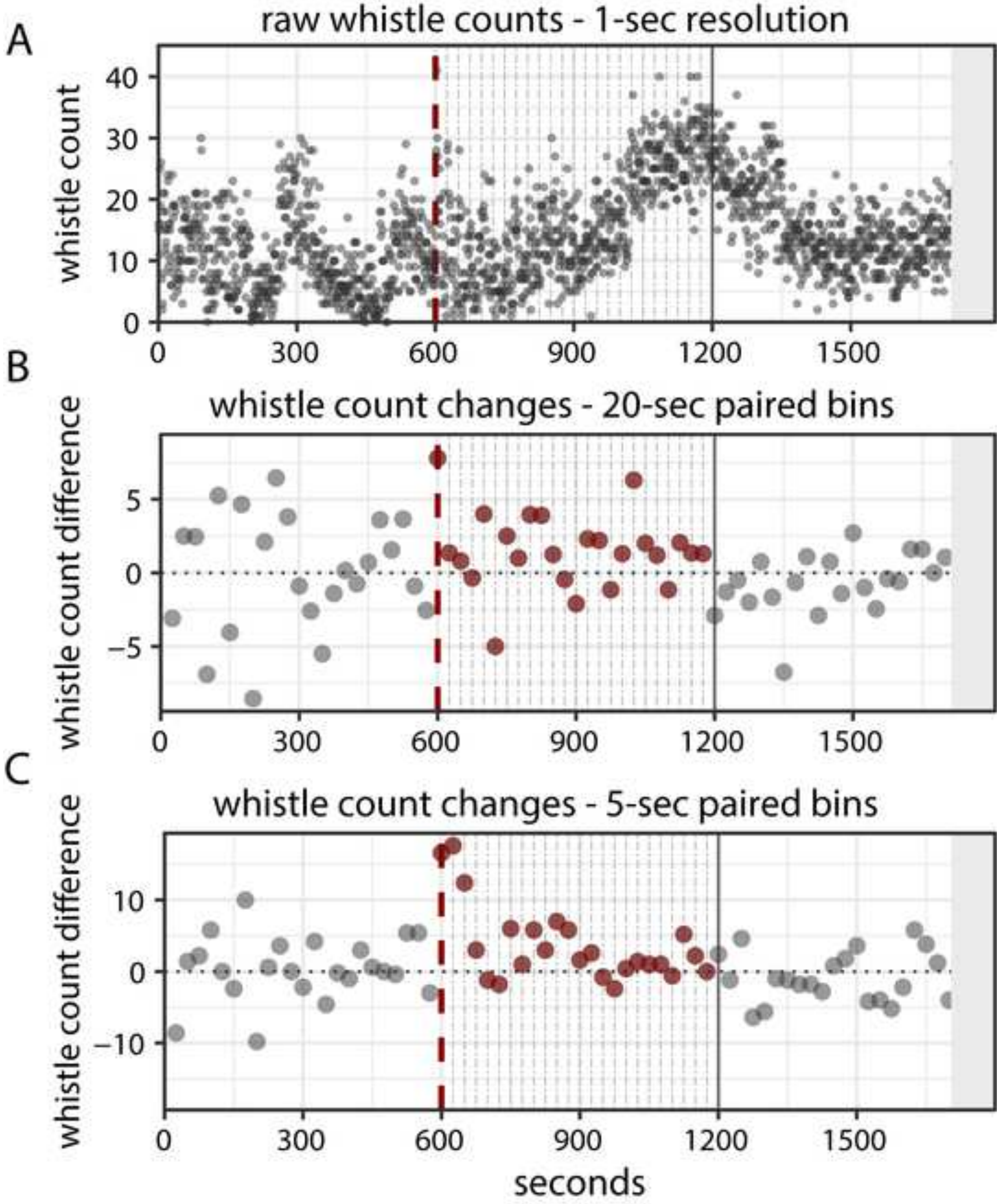
A
raw whistle counts - 1-sec resolution
whistle count
0
10
20
30
40
0
300
600
900
1200
1500
B
whistle count changes - 20-sec paired bins
whistle count difference
−5
0
5
0
300
600
900
1200
1500
C
whistle count changes - 5-sec paired bins
whistle count difference
−10
0
10
0
300
600
900
1200
1500
seconds

Click here to access/download
Supporting Information
S1_Fig.pdf

Supporting Information

Click here to access/download
Supporting Information
S1_File.pdf

Click here to access/download
Supporting Information
S2_Fig.pdf

Click here to access/download
Supporting Information
S3_Fig.pdf

Full title: Common dolphin whistle responses to experimental mid-frequency sonar

Formatted: Centered

Short title: Dolphin acoustic response to sonar

*Caroline Casey[a,b]¶, *Selene Fregosi[a]¶, Julie N. Oswald[c], Vincent M. Janik[c], Fleur Visser[d,e], Brandon Southall[a,b]

[a]Southall Environmental Associates, Inc., ., 9099 Soquel Drive, Aptos, California, United States of AmericaA 95003, USA

[b]Institute of Marine Sciences, University of California Santa Cruz, 115 McAllister Way, Santa Cruz, CA 95060, USACalifornia, United States of America

[c]Sea Mammal Research Unit, Scottish Oceans Institute, School of Biology, University of St. Andrews, St. Andrews, KY16 8LB, United Kingdom

[d]Kelp Marine Research, 1624 CJ, Hoorn, the Netherlands

[e]Department of Coastal Systems, Royal Netherlands Institute for Sea Research, P.O. Box 59, 1790 AB Den Burg, Texel, the Netherlands

*

Corresponding author

E-mail: cbcasey@ucsc.edu

¶CC and SF are Joint Senior Authors of this work. : Caroline Casey, cbcasey@ucsc.edu

Abstract

Oceanic delphinids around naval operations are regularly exposed to high source levelintense military sonar broadcast within the frequency range of in their hearing range on a regular basis. H; however, empirically measuring the its impact of sonar on on the behavior of highly social, free-ranging dolphins has proven logistically is challenging. Additionally, baseline variability or the frequency of vocal state-switching among social oceanic dolphins during undisturbed conditions is lacking among social oceanic delphinids, making it difficult to attribute changes in vocal behavior to anthropogenic disturbance. Using a network of drifting acoustic buoys in a three-phased controlled exposure experiments, we investigated the effects of mid-frequency (3-4 kHz) active sonar (MFAS) on whistle production in short-beaked (*Delphinus delphis delphis*) and long-beaked common dolphins (*Delphinus delphis bairdii*) in southern California. Given the complexity of acoustic behavior exhibited by these group-living animals, we conducted our response analysis over varying temporal windows (10 min – 5 s) to describe both longer-term and instantaneous changes in sound production. We found that common dolphins exhibited an acute and pronounced changes in whistle rate in the 5 s following exposure to experimentalsimulated Navy sonarMFAS. This response was sustained throughout repeated sonarsequential MFAS exposures within an experiments simulating operational conditions to sonar in the experiment, suggesting that dolphins may not habituate to this disturbance. These results indicate that common dolphins exhibit a brief yet significantclearly detectable acoustic responses to MFAS. They also and highlight how different-sizedvariable temporal analysis windows – tuned to key aspects of baseline vocal behavior as well as experimental parameters related to MFAS exposure – impactenable the detection of behavioral responses. We suggest that future work with oceanic delphinids explore baseline vocal rates a-

Formatted: Font: 18 pt

Formatted: Don't add space between paragraphs of the same style

priori and use information on the rate of change in vocal behavior to inform the analysis time window over which behavioral responses are measured.

Millions of oceanic dolphins are exposed and presumably affected by military sonar annually; however empirically measuring its impact on the behavior of free-ranging dolphins has proven logistically challenging. Additionally, baseline variability or the frequency of vocal state-switching during undisturbed conditions is lacking among social oceanic delphinids, making it difficult to attribute changes in vocal behavior to anthropogenic disturbance. Using a network of drifting acoustic buoys and a three-phased controlled exposure experiment, we investigated the effects of mid-frequency (3-4 kHz) active sonar (MFAS) on whistle production in short-beaked (*Delphinus delphis*) and long-beaked common dolphins (*Delphinus bairdii*) in southern California. Given the complexity of acoustic behavior exhibited by these group-living species, we conducted our response analysis over varying temporal windows (10 min – 5 sec) to describe both longer-term and instantaneous changes in sound production. We found that common dolphins exhibited an acute and dramatic change in acoustic behavior in the 5-seconds following exposure to experimental Navy sonar. In some cases, this response was sustained throughout repeated exposures to sonar in the experiment, suggesting that dolphins may not habituate to this kind of disturbance. These results suggest that common dolphins exhibit a brief, yet dramatic acoustic response to MFAS, and highlights how different sized temporal windows – tuned to key aspects of baseline vocal behavior as well as experimental parameters related to MFAS exposure – impacts the detection of behavioral responses. We suggest that future work with oceanic delphinid species explore baseline vocal rates a-priori and use information on the rate of change in vocal behavior to inform the analysis time-window over which behavioral responses are measured.

Introduction

Sound production and reception play a critical role in the lives of cetaceans, aiding in important life-history events, including maintenance of social relationships, coordination of group movement, foraging, and evasion of predators [(1]). Consequently, substantial effort has been directed toward describing cetacean acoustic behavior [(2,3]) and evaluating how it is impacted by human-generated disturbance [(4-8]). There are many sources of anthropogenic noise pollution (*e.g.*, vessel noise, oil and gas exploration, construction and facilities maintenance, fisheries and aquaculture, military activity) which Many sources of anthropogenic noise pollution (*e.g.*, vessel noise, oil and gas exploration, renewable energy, coastal construction and maintenance, fisheries and aquaculture, and military activity) can have varying short and long-term impacts on marine mammal behavior and health and behavior [(4, 9-11]). Concentrated research efforts aimed at characterizing these impacts have led to the systematic development of acoustic exposure criteria, informing and improving effective management strategies for both to characterize these impacts have led to the systematic development of acoustic exposure criteria, informing and improving effective management strategies for regulators and industries (for reviews on auditory and behavioral criteria, see 12). Such assessments and criteria have also highlighted species and noise exposure contexts for which information is sparse or unavailable.

Among cetaceans, oceanic delphinids represent an importantan essential and logistically challenging group of species for which to evaluate the impact of anthropogenic noise on vocal

Formatted: Indent: First line: 0.5", Don't add space between paragraphs of the same style

Formatted: Font: 18 pt

Formatted: Indent: First line: 0.5"

Formatted: Font: Italic

Formatted: Indent: First line: 0.5"

behavior. These animals are highly soniferous, abundant, and often extremely gregarious (pods of > 500 individuals are common for some species). Sound production has been demonstrated to play a particularly importanta vital role in the regulation ofthe maintenance of social interactions relationships and cohesion among group members (*e.g.,* [2,3]). Oceanic delphinids are ubiquitous around some U.S. Navy operational areas where mid-frequency active sonar (MFAS; 1-10 kHz) is commonly used for submarine detection in training exercises and warfare, resulting in associated large numbers of sonar exposures for these federally protected species. Some of the most powerful MFAS systems (*e.g.,* AN/SQS-53C) use emit repeated pings with fundamental frequencies in the 3-4 kHz range, ping lengths of approximately 1-3 seconds, and effective nominal source levels as high as 235 dB dB re 1 µPa at 1 m root-mean-square (RMS) re 1µPa that may be transmitted for several minutesminutemins to hours at high duty cycles (more than 1 ping/min) [(13]). Aside from the elevated background noise and potential disturbance that may result from these training exercises [(*e.g.,* 14)], MFAS signals overlap with the frequencies that oceanic dolphins commonly rely on for social sound (whistle) production. Delphinid whistles are narrowband tonal sounds with most of the acoustic energy concentrated below 20 kHz [(14-1615]).

While MFAS has been linked to mass stranding events of cetaceans [(13,167]) and its effect on cetaceans has been experimentally evaluated in a handful of species (for a recent review, see [179]) , the effectsimpact of sonar on the acoustic behavior of oceanic delphinids have not been systematically explored. This is due in large partmainly due to the logistical challenges of applying previously developed methods used in other behavioral response studies of individual animals to large aggregations of dolphins. Much of the prior research on cetacean

Formatted: Font: (Default) Times New Roman, 12 pt

Commented [SF1]: I would leave this spelled out here vs when it comes after a number leave it short. I think that still addresses their comment but is consistent

Formatted: Indent: First line: 0.5"

behavioral responses to noise has capitalized on the use ofusing suction-cup attachedsuction-cup-attached motion-sensing and acoustic recording tags to characterize responses following controlled exposure to MFAS [(*e.g.,* 21-2318-20]). Unfortunately, such tags are challenging to deploy and are easily shed by small dolphins due to the tag size relative to the smaller body surface of dolphins, and high drag due to fast swimming speeds, and frequent physical social contact. Additionally, oceanic delphinids commonly occur in large groups that display remarkable coordination, making the collective vocal behavior of the group perhaps a more appropriate focus of analysis [(210]). Opportunistic passive acoustic studies relying on large, cabled hydrophone arrays have been used to quantify changes in vocal activity and thus act as a proxy for the presence of or absence of multiple animals before, during, and after exposure to MFAS [(224,235). Such experiments are valuable but require extensive, high-cost moored hydrophone arrays with restricted spatial coverage.

Previous studies on acoustic responses of oceanic dolphins to Navy sonar have observed Findings from previous studies of acoustic responses to Navy sonar in oceanic delphinids include shifting specific frequency components of whistle contours, increasing or decreasing calling rate, increasing call amplitude, and even mimicry of MFAS elements [(14, 236-258). For example, tagged orcas killer whales (*Orcinus orca*) adjust the high-frequency component of their whistles during sonar exposure, and increase the number and amplitude of their calls following each ping [(247]). False killer whales *(Pseudorca crassidens)* appear to increase their whistle rate and produce more MFAS-like whistles after exposure to simulated sonar [(256]).

Formatted: Font: (Default) Times New Roman, 12 pt

Formatted: Indent: First line: 0.5"

One opportunistic study provided initial insights into the behavioral responses of some social oceanic delphinids to MFAS. Bottlenose dolphins (*Tursiops truncatus*), common dolphins (*Delphinus sp.*), Pacific white-sided dolphins (*Lagenorhynchus obliquidens*), and Risso's dolphins (*Grampus griseus*) exposed to incidentalincidentally exposed to MFAS showed a cessation of vocalizations, an increase in the intensity of vocalizations, or a combination of both [(14]). Of all delphinid species, common dolphins displayed the widestbroadest range of responses, including changing their behavioral state or direction of travel when sonar stopped, increasing the intensity of vocalizations when sonar began, vocalizing very little or not at all during sonar exposure, or a combination of these observations [(14]). These results are consistent with a more recent opportunistic evaluation of delphinid responses to an underwater explosionan underwater detonation, which showed that whistle rate, complexity, and frequency content all varied in response to the explosive event [26](30). Depending upon the frequency, intensity, and consistency of these kinds of noise exposures, such behavioral changes could result in physiological consequences that impact overall population health [(2731]). Unfortunately, information on baseline variability or and the frequency of vocal state-switching during undisturbed conditions is lacking among free-ranging, social, oceanic delphinids, making it challenging to interpret the responses observed. While opportunistic studies of delphinid acoustic responses to sonar are insightful, a detailed assessment under controlled experimental conditions is needed to understand the extent to which delphinids are impacted by this kind of disturbanceMFAS impacts oceanic delphinids.

Quantifying vocal behavior in these taxa is complicated by the fact that dolphin acoustic behavior is dynamic, variable, and influenced by a myriad of social and environmental factors

Formatted: Indent: First line: 0.5"

Formatted: Indent: First line: 0.5"

[28](32). Measurements of acoustic behavior and how it changes in response to disturbance must be madeAcoustic behavior and how it changes in response to disturbance must be measured across some predetermined interval of timetime interval. In previous cetacean behavioral response studies using a conventional controlled exposure experimental (CEE) design, this temporal window was often dictated by logistical limitations of the technology being used (*e.g.*, battery power of tags, the feasibility of continuous behavioral observations, etc.), or designed to match the duration of anthropogenic noise source being evaluated. However, averaging vocal behavior over long time windows may result in missing instantaneous or shorter duration responses that occur at the onset of exposure or at scales that are more biologically meaningful to the individuals exposed. One way to address this is to examine acoustic metrics computed over a variety ofvarious time windows to determine if and/orand when we can attribute a change in vocal behavior to a known, controlled disturbance.

For this study, different broad and fine-scale analytical approaches were used to investigate the Here, we apply and compare different broad and fine-scale analytical approaches to analyzing delphinid vocal behavior and effects of experimental controlled MFAS on whistle production using experimental methods in short-beaked (*Delphinus delphis delphis*) and long-beaked common dolphins (*Delphinus delphis bairdii*) in southern California. By assessing group-level vocal behavior across different time scales, we aimed to:

1. Describe the variability in baseline vocal behavior of common dolphin aggregations during control conditions.

1.

Formatted: Font: (Default) Times New Roman, 12 pt

Formatted: Indent: First line: 0.25"

Formatted: Font color: Black

Formatted: Numbered + Level: 1 + Numbering Style: 1, 2, 3, … + Start at: 1 + Alignment: Left + Aligned at: 0.25" + Indent at: 0.5"

2. Characterize Compare the types of vocal responses detected during a controlled exposure to MFAS using broad and fine-scale temporal resolution. across broad and fine temporal scales.

Given the need to establish sampling regimes that can be applied and compared across studies, our objective isour objective is to provide an informative framework for assessing the complex acoustic behavior exhibited by group-living species. We highlight how using different sizeddifferent-sized temporal windows – tuned to key aspects of baseline vocal behavior as well as experimental parameters related to MFAS exposure – impacts the detection of behavioral responses.

Materials and methodsethods

Formatted: Font: 18 pt

A. General Methods

Controlled exposure experiments (CEEs)CEEs were conducted with two subspecies of common dolphins. This study was as part of a broader effort aimed at quantifyingto quantify group-level responsiveness of oceanic delphinids to military sonar using CEEs. Common dolphins occur as two separate species within our study area (33), around Santa Catalina Island, located off the coast of southern California, USA. Since they regularly occur in mixed-species groups, we pooled data for the two speciessubspecies to describe baseline vocal data and included speciessubspecies as a potential explanatory variable in our models. The project integrateds multiple different data streams, including shore-based tracking of dolphin schoolspods, passive acoustics to record vocal activity, and photogrammetry to measure fine-scale behavior [29](34). This work was conducted between 2017-2021. We chose our study area because it lies near the Southern California Offshore Range (SCORE) – a tactical training area for the U.S. Navy Pacific

Fleet located off the west side of San Clemente Island – where animals regularly encounter the types of signals we used in our experiment.

CEEs were composed ofcomprised three discrete phases: pre-exposure (baseline), exposure using intermittent simulated MFAS signals, and post-exposure. In a subset of experiments, no MFAS signals were broadcast during the exposure phase, serving as controls.In control trials, the equipment was lowered into the water from the exposure boat but no MFAS signals were broadcast. For details about the experimental source and sound source characteristics – including calculations of received levels – see Durban et al. 2022 [29]. 2022 (34). Each experimental phase was 10 minuteminS in duration. During exposure phases, MFAS ‘pings’ of 1.6 s in duration consisting of three tonal and frequency modulated elements between 3.5-4 kHz were transmitted (see:frequency-modulated elements between 3.5-4 kHz were transmitted Southall et al., 2012 (35) for additional details on the sound source and signal parameters). Pings were emitted at a broadband source level of 212 dB re 1µPa RMS every 25 seconds, which is similar in repetition rate, duty cycle, and the absence of a ramped-up source level (as used in some previous MFAS CEEs) to some certain active Navy MFAS systems (*e.g.*, helicopter-dipped sonar systems). Up to 24 total pings were emitted per 10-minutemin exposure phase, provided that no permit-mandated shutdowns occurred for animals occurring no permit-mandated shutdowns were implemented for animals within 200 m of the active sound source (this occurredhappened in only one playbackCEE). The sound source was positioned relative to focal animal groups using noise sound propagation modeling to ensure received levels at focal animals occurred at maximum levels ofwere no greater than 140-160 dB dB re 1µPa RMSRMS.

Formatted: Indent: First line: 0.5"

For every CEE, speciessubspecies identity (based on differences in genetics, morphology, and pigmentation) was determined using a combination of aerial images obtained from drone footage, genetic sequencing from biopsy samples, and visual observation. Additionally, group size was characterized by experienced shore-based observers using strong magnification binoculars or a binocular scope located at elevated locations (~70 m) that enabled, enabling a broad overview of the research area (up to 20 km from shore). Shore-based tracking of animals could be conducted for groups up to 7 km from shore. Focal follows included estimation ofestimating low, best, and high group size, the number of subgroups (defined as all individuals in closer proximity to each other than to closer to each other than other individuals in the area), the range of inter-individual spacings within subgroups, and distances between subgroups. These observations were taken continuously throughout the experiment at 2-minutemin intervals. For a fullcomplete description of each of these methods, see Visser et al., 2014 (36) and Durban et al., 2022 (34).[29,30]

B. Acoustic dData cCollection and pProcessing

1. Passive aAcoustic mMonitoring

Passive acoustic recordings were obtained from each target group of dolphins using three drifting, remote-deployed acoustic recording units. Up to three separate recording units were tactically positioned and recovered from a single small (~6 m) rigid-hull inflatable boat, with the objective of placing one recording unit within 500 m of the predicted trajectory of the dolphins during each CEE phase (Fig. 1). Each recording unit consisted of a surface buoy and flag with an underwater recorder suspended by 10 m of line. The recorder was either a SoundTrap ST300

Formatted: Indent: First line: 0.5"

Formatted: Font: 16 pt

Formatted: Font: 16 pt

Formatted: Font: 16 pt

Formatted: Font: 16 pt

Formatted: Font: 16 pt

Formatted: Font: 14 pt, Not Italic

Formatted: Level 2

Formatted: Font: 14 pt, Not Italic

Formatted: Font: 14 pt, Not Italic

Formatted: Font: 14 pt

Formatted: Indent: First line: 0.5"

(Ocean Instruments NZ, Auckland, New Zealand) or a SNAP Recorder (Loggerhead Instruments, Sarasota, FL, USA). Both recorded via a single omnidirectional calibrated hydrophone (SoundTrap: integrated hydrophone, frequency response 0.02–60 kHz ± 3 dB re 1µPa, end-to-end sensitivity -178 dB re 1µPa/V; SNAP: HTI-96-MIN hydrophone, frequency response 1.0-20 kHz ± 3 dB re 1µPa, end-to-end sensitivity -164 dB re 1µPa/V, frequency response 1.0-20 kHz ± 3 dB) which was suspended by a shock-mounted cable at a depth of 10 m. All recording units had a Global Positional System (GPS) tracking device (Trace, SPOT LLC, Chantilly, VA, USA) that recorded the location of the instrument once every minutemin (Fig. 1). Five-minutemin WAV files were continuously recorded at either 96 kHz sampling rate with 16-bit resolution (SoundTrap) or a 96 kHz sampling rate with 44.1 kHz sampling rate with a 16-bit resolution (SoundTrap) or a 44.1 kHz sampling rate with a 16-bit resolution (SNAP).

Figure 1. A schematic representation of the placement of acoustic recorders (see inset for floating acoustic recording unit) relative to the track of the focal group of dolphins. A single buoy and associated hydrophone were placed within 500 m of the animals during the pre-exposure, exposure, and post-exposure period. The first buoy is placed within 500m of the animals during the pre-exposure, the second during the exposure, and the third during the post-exposure period. The sound source is located at approximately 1 km from the dolphins at the onset of the exposure period. Note that the source vessel was idling in neutral and was not moving throughout the duration of the exposure period. The dashed line with associated arrows represents the movement path of the focal group. Note theThe shore station monitoring the group was positioned on landb and is denoted by the theodolite symbol. presence of the shore station

Formatted: Font: Not Bold

Formatted: Font: Not Bold

Formatted: Font: Not Bold

positioned on land.The map was inspired by images from the NASA Earth Observatory (public domain) and was not drawn to scale (for illustrative purposes only).

To evaluate which PAM recording unit was closest to the focal group given the dolphins' frequently unpredictable course, the relative proximity of each hydrophone to the animals was determined *post-hoc*Given the dolphins' frequently unpredictable course, the relative proximity of each hydrophone to the animals was determined post-hoc to evaluate which PAM recording unit was closest to the focal group. The animals' location was known from an associated octocopter drone flight (APO-42, Aerial Imaging Solutions) that utilized a micro 4/3 digital camera (Olympus E-PM2) and 25 mm lens (Olympus M. Zuiko F1.8) mounted to a gimbal. centered over the focal The octocopter flew group at approximately 60 m directly above the dolphins to provide sufficient pixel resolution while decreasing the potential for disturbance [(see 2934 for details]). The relative distances (in meters) between the focal group (from the drone's GPS) and each recorder (from their flag-mounted GPS units) were estimated for every minutemin of the 30-minutemin experiment using the Haversine formula and linear interpolation in in a custom MATLAB script (Mathworks, Natick, MA, USA, Fig. 2). Recordings from the buoy closest to the focal group at 1-minutemin intervals were used for all subsequent analyses. Any recordings made when a recorder s that exceeded 1600 meters1.6 km from the focal group (even if ththe recorder was the closest availableey were the closest recorder deployed) were excluded. This threshold was selected based on a previous assessment of detection ranges of playbacks of odontocete whistles (10-20 kHz) by bottom-mounted hydrophones in southern California, which demonstrated a 95% probability of detection of a 135 dB re 1 µPa dolphin whistle at 1.600 meters km with an SNR of 2.2 dB re 1µPa [31](37). This assessment was

Formatted: Indent: First line: 0.5"

supported by the drop-off in whistle amplitude observed in the spectrograms (*post-hoc*) when any buoy surpassed 1600-18001.6-1.8 km distance from the focal group (Fig. 2). After accounting for this distance cut-off, 9.25 total hours of recordings remained and were used in subsequent analysis.

Figure 2. Spectrograms of each of the three recorders strategiciallystrategically placed on the tracklinetrack line of a moving group of dolphins and associated whistle countsdetections from the buoy closest to the animals at each minute. from each of the three recorders that were strategically placed on the track-linetrack line of a moving group of dolphins. The solid white line represents the distance between the recorder and the drone flight centered over the focal group (units on the right y-axis), the red dashed vertical lines denote the experimental period, and the horizontal white dashed line marks the 1600 m1.6 km threshold. Estimates of the relative distance between the focal group and each of the recordersrecorder werewere produced assessed every minutemin of the 30-minutemin experiment. The bottom panel shows the number of whistles detected on the closest recorder using the PAMGuard Whistle and Moan Detector. Times when the closest buoy switched is indicated by the blue dashed lines and associated blue numbersThe blue dashed lines and associated blue numbers indicate times when the closest buoy switched and which buoy was closest. The grey area denotes where whistle detections were excluded due to the recorder distance exceeding 1.6 km.MFAS pings denoted by the vertical lines on the spectrogram during the experimental period.

2. Quantifying whistle production and variability

Formatted: Font: 14 pt, Not Italic

Formatted: Font: 14 pt

While common dolphins are known to emit buzzes, echolocation clicks, and whistles, we focused our efforts on characterizing whistle production since they are the keycritical signal for long distancelong-distance communication and play a majorsignificant role in group cohesion and coordination [(2], 38). Extraction of whole whistle contours in high background noise with overlapping whistles is extremelyexceptionally challenging and results in high rates of missed detections, irrespective of methodology. To create a dataset in which error rates were kept constant across different phases of the experimentexperimental phases, we used the Whistle and Moan Detector (WMD) module in PAMGuard (v 2.01.05), 39)[32]. The WMD deals with uncertainties by only detecting parts of whistles that clearly stand out above noise using standardized settings across extractions. It is important to note that this often leads to a fragmentation of whistles, with one whistle being split into several independent sections. Thus, whistle counts detections reported here are not comparable to ones obtained with whole whistle extraction in other studies. However, for our assessment of changes in vocal activity between different experimental phases, it was more important to keep error rates constant to allow for relative comparisons. A qualitative assessment of detector performance within each CEE ensured that variation in whistle detections accurately reflected variation in whistle activity observed in the spectrograms.

Formatted: Indent: First line: 0.5"

Formatted: Font color: Text 1

Formatted: Font: Times New Roman, 12 pt, Font color: Text 1

Formatted: Font color: Text 1

The WMD operates on the spectrogram output of the PAMGuard Fast Fourier Transform (FFT) Engine module. We optimized settings for the FFT Engine to provide comparable frequency and temporal resolution of the calculated spectrograms across the two recorders and sampling rates. For the SoundTrap recorders, which had a sampling rate of 96 kHz, the FFT Engine module calculatedcomputed spectrograms with an FFT length 1024, hop size of 1024,

Formatted: Indent: First line: 0.5"

hop size of 512, and a Hann window. This resulted in providing a frequency resolution of 93.75 Hz and time resolution of 10.67 ms. For the SNAP recorders, which had a sampling rate of 44.1 kHz, spectrograms were calculated with a Hann window, FFT length 512 and hop size 256, and hop size 256, resulting resulting in a frequency resolution of 86.13 kHz and time resolution of 11.61 ms. The WMD was set to detect whistles between 5 kHz and 20 kHz to exclude detection of the tonal sounds from the simulated mid-frequency sonar playbacks source (below 5 kHz) and to standardize the upper detection limit across the two sampling rates and avoid any possible edge effects near the Nyquist frequency of the lower sampling rate. The detection threshold was set at 6.0 dB re 1µPa. Full WMD settings can be foundare in the supplementary materials (S1 AppendixFig). While the fundamental sonar tonals were excluded by the 5 kHz low-pass cuthigh-pass cut-off for detections, the high source level of the simulated MFAS resulted in the presence of harmonics in some of the recordings. All harmonics were manually annotated in PAMGuard Viewer using the Spectrogram Annotation module for later removal.

Commented [SF2]: This would be high-pass, correct? Or just 'low cut-off'?

Detected wWhistles were automatically exported from the PAMGuard detection database using the PAMGuard MATLAB tools (https://github.com/PAMGuard/PAMGuardMatlab). Annotated MFAS harmonics were removed using and R package `PAMmiscPal` in R version 4.3.1 [33,3432,33](40). MFAS harmonics were removed, and Wwhistles were quantified at 1--second resolution; . Bbecause whistles are were often longer than 1-second1 s in duration, the total number of whistles starting within a 1-second bin was counted, providing a metric for whistle activity as detected whistles detections per second. For brevity, this is referred to as whistle count throughout the remainder of this manuscript.

Formatted: Indent: First line: 0.5"

C. Characterization of baseline vocal behavior

1. Overall whistle count

To assess common dolphin vocal behavior in control conditions, we calculated the mean and median whistle count per second for the full 30-minute experiment for each control CEEWe calculated the mean and median whistle count per second for the entire 30-min experiment for each control CEE to assess common dolphin vocal behavior under control conditions. These data were evaluated with reference to concurrent data collectgroup size estimates collecteded by experienced shore-based focal follow observations that quantified group size. observers.

2. Changepoint analysis

To describe the natural variability in vocal behavior during control conditions, we applied a changepoint analytical approach to the control CEE data collected for both common dolphin speciesWe applied a changepoint analytical approach to the control CEE data collected for both common dolphin subspecies to describe the natural variability in vocal behavior during control conditions. Change point detection is used to pinpoint times when the probability distribution of a time series changes (*i.e.*, vocal state changes). The aim is to identify times at whichwhen either the mean or variance deviates from the expected trends in the dataset and estimate the number and position of all changepoints. Effectively, this approach detects points in time when a significant change in whistle count occurs. First, a 5--second smoothing window was applied to the raw 1-second whistle count data. Then, changepoints in both mean whistle count and whistle count variance were detected using the 'changepoint' package in R version 2.2.4 [(34-35]41). The "BinSeg" (Binary Segmentation) algorithm was used. This provided the number and

Formatted: Font: 16 pt

Formatted: Font: 14 pt, Not Italic

Formatted: Font: 14 pt

Formatted: Indent: First line: 0.5"

Formatted: Font: 14 pt, Not Italic

Formatted: Font: 14 pt

Formatted: Indent: First line: 0.5"

locations of all state changes in both the mean and the variance of whistle count over the total the mean and the variance of whistle count over the 30-minutemin sampling period.

D. Assessing the impact of disturbance on vocal behavior

Formatted: Font: 14 pt

We employed a hierarchical approach to characterizing the types of vocal responses that might be detected during controlled exposure to MFAS using broad and fine-scale time windows Using broad and fine-scale time windows, we employed a hierarchical approach to characterizing the types of vocal responses that might be detected during controlled exposure to MFAS (Fig. 3). All statistical analyses were carried out using R version 4.3.1 [34](R Core Team 2023).

Formatted: Indent: First line: 0.5"

Figure 3. Flowchart of methods implemented to assess changes in common dolphin vocal behavior during controlled exposure to MFAS. Methods include pre-processing of acoustic data and to baseline vocal behavior analysis and as well as a hierarchical assessment of disturbance on vocal behavior at four three temporal scales. Alpha-numeric references corresponds to the sub-heading in the methods section.

1. Difference in changepoints by period across CEEs

Formatted: Font: 14 pt, Not Italic

Formatted: Font: 14 pt

We conducted a changepoint analysis on all CEEs (both controls and MFAS) to evaluate whether common dolphins change the frequency of vocal state switching as a result of exposure to MFAS. We used the same general method as in Section C.2described above but quantified the number of changepoints in the 10-minutemin pre-exposure and 10-minutemin exposure periods separately. Changepoints were detected for both the mean and variance of the whistle count data.

Formatted: Indent: First line: 0.5"

The difference in the number of changepoints between the two periods was calculated, and an unpaired t-test was used to evaluate any significant differences between controls and MFAS experiments.

2. Characterize the impact of MFAS exposure on whistle count: 10-minutemin time scale

Formatted: Font: 14 pt, Not Italic

Formatted: Font: 14 pt, Not Italic

Formatted: Font: 14 pt

To identify potential broad scale changes in whistle count in response to a simulated MFAS exposurebroad-scale changes in whistle count in response to simulated MFAS exposure, we pooled and analyzed all CEEs (both controls and MFAS exposures) and analyzed them using a Generalized Linear Mixed Model (GLMM) approach, implemented using R package `glmmTMB` [(36542]). We modeled the absolute difference in median whistle count between the 10-minutemin pre-exposure period and the 10-minutemin exposure period (*periodDiff*) as a function of CEE type (*ceeType*; either control or simulated MFAS), a random identity variable (*ceeNum*), dolphin speciessubspecies (*subSspecies*), the best estimate of total group size from the shore basedshore-based observers (*groupSize*), and the mean distance between the focal group and the closest buoy for the fullentire CEE (*buoyDistance*). Using the absolute value for difference in median whistle count enabled us to explore the magnitude of a potential response. We modeled the relationship using a negative binomial distribution, which fit the count-type data after the transformation. Our full model was:

Formatted: Font: Italic

Formatted: Font: Italic

Formatted: Font: Italic

Formatted: Font: Italic

Formatted: Font: Italic

Formatted: Font: Italic

periodDiff ~ ceeType + ceeNum + subSspecies+ groupSize + buoyDist

Formatted: Font: Italic

We used backward elimination, ΔAIC, and analysis of variance (ANOVA) to select the best model. We used backward elimination and resulting AIC scores to select our final model and present the results of the final model below.

3. Characterize the impact of MFAS exposure on whistle count: 20-second time scale

Formatted: Font: 14 pt, Not Italic

Formatted: Font: 14 pt

Formatted: Indent: First line: 0.5"

To characterize more instantaneous changes in whistle production in response to MFAS exposure, we compared dolphin whistle counts detections in the 20 seconds before and 20 seconds after each individual ping (n = 24 1-second pings per 10-minute experimental period, ~25 seconds between each ping) for both MFAS experiments and controls. We selected this time window to capture sustained variation in whistling behavior within a single ping cycle, without overlap between cycles. Differences between these two sequential time bins were calculated by subtracting the mean whistle count for the first bin from the mean whistle count of the second bin (Fig. 4B). The first ping started at time 0, the second ping at timeat 25 seconds, and so on. Because no actual pings were present in the control experiments, we calculated the change in whistle count surrounding 'ghost pings' which were chosen to be timed at the same time as when real pings would have occurred during atime points placed at the same time as when actual pings would have occurred during an MFAS CEE.

Figure 4. Example plots of (A) raw whistle counts detections over time, (B) changes in whistle count between 20 second duration sequential bins, and (C) changes in whistle count between 5 second duration sequential bins, for the pre-exposure, exposure,20-s duration sequential bins, and (C) changes in whistle count between 5-s duration sequential bins for

the pre-exposure, exposure, and post-exposure period within one CEE. The dashed vertical red line indicates the onset of exposure, and the sequential gray dashed lines represent each ping within the exposure period.. Pre-exposure and postPost-exposure periods were not included in the modeling analysis but are presented here for reference. The grey shaded area denotes where whistle detections were excluded due to the recorder distance exceeding 1.6 km.

Similar toLike the analysis at the 10-minutemin time scale, we used a generalized linear mixed modelGLMM approach (using R package `glmmTMB`) to identify potential significant differences immediately following pings (*pingChange*) in MFAS experiments compared to controls where no pings were present. In addition to the previous fixed effects included at the 10-minutemin scale (*ceeType, ceeNum, subSspecies, buoyDistance, and groupSize*), we also included median whistle count per second for the entire experimental period to account for the varied baseline whistling activity across CEEs (*medWhist*).

pingChange ~ ceeType + ceeNum + subSspeciessubspecies + groupSize + buoyDist + medWhist

Binned ping change data were generally normally distributed, but were zero- inflated, so we used a Gaussian distribution for the primary model and additionally modeled the zero inflation as a function of median whistle count (*medWhist*). We used backward elimination, ΔAIC, and analysis of variance (ANOVA) to select the best model.

4. Characterize the impact of MFAS exposure on whistle count: 5-second time scale

Formatted: Font: Not Bold

Formatted: Font: Not Bold

Formatted: Indent: First line: 0.5"

Formatted: Font: Italic

Formatted: Indent: First line: 0.5"

Formatted: Font: 14 pt, Not Italic

We repeated the analysis conducted abovein section D3, but over a shorter 5 second5-s time window to investigate potential shorter durationinstantaneous changes immediately following pings (Fig. 4C).

Similar toLike the above analysis, we used a generalized linear mixed modelGLMM approach to identify potential significant differences in whistle count changes in the 5 seconds following compared to the 5 seconds before each ping (*pingChange*) for MFAS experiments compared to controls. We used the same fixed effects implemented at the 20-second scale (*ceeType, ceeNum,* *[subSspecies, buoyDistance, and groupSize, medWhist)* but also included an autocorrelation structure to this analysis *AR(1)* to account for clearapparent temporal lag effects in exploratory plots.

$$pingChange \sim ceeType + ceeNum + subSspecies + groupSize + buoyDist + medWhist + AR(1)$$

Like the 20-ssecond scale, binned ping change data were normally distributed and zero- inflated; a Gaussian distribution was used for the conditional model and zero-inflation was modeled as a function of median whistle count (*medWhist)*. We used backward elimination, ΔAIC, and ANOVA to select the final model.

5. CEE-by-CEE analysis

We assessed each playback individually at each time scale to better contextualize the severity and , persistence of responses and whether dolphins increased or decreased their whistle behavior following sonar exposure., and directionality (*i.e.*, increase or decrease in whistle

Formatted: Indent: First line: 0.5"

Formatted: Font: Italic

Formatted: Font: 14 pt, Not Italic

Formatted: Font: 14 pt

Formatted: Indent: First line: 0.5"

count) of responses, which were not measured by either the modeling approach or changepoint analysis. The methods and results for the CEE-by-CEE analysis can be found in the supplementary materials (S12 FileAppendix).

Ethics Statement

Animal research was conducted under authorization of the United States National Marine Fisheries Service marine mammal research permits 19116 and 19091.

Results and discussion

A. Characterization of baseline vocal behavior

1. Basic dDescription

This analysis includes nine control experiments, each conducted on separate days. Four control of these experiments were conducted with *Delphinus delphis*short-beaked common dolphins, and five were conducted with Delphinus bairdiilong-beaked common dolphins (Table 1). This resulted in 270 minuteminss of baseline acoustic data for both speciessubspecies (pooled). The average group size across speciessubspecies was 190 individuals (range 45-300 animals). The dispersion of of animals varied considerably within and between control experiments, including small to large groups (55-300 individuals) in tight to looselose organization, joint (in a single group with no subgroups), or spread out over several subgroups (range: 2-6) at tens to several hundreds of meters apart (range: 10-800 m).

Formatted: Don't add space between paragraphs of the same style

Formatted: Font: 18 pt

Formatted: Font: 18 pt

Formatted: Font: 16 pt

Formatted: Font: 14 pt, Not Italic

Formatted: Font: 14 pt, Not Italic

Formatted: Font: 14 pt

Formatted: Indent: First line: 0.5"

Formatted: Font: Not Italic

Whistles were successfully detected across all control experimental deployments. Mean (SD) and Median (IQR) whistle count per second varied between control CEEs (Table 1). In our assessment of the control experiments, we found the total number of whistles varied between 0.3-4.6 whistles/second. Note, however, that the inherent uncertainty error in our group size assessment for large groups did not allow us to calculate accurate whistle rates/individual (see Ttable 1 for group size estimates and whistle countsdetections).

Table 1. Summary of each controlled exposure experiment, including controls (no sound emitted) and 'simMFAS'MFAS (playback of simulated mid-frequency active sonar).

CEE ID	speciesSsubspecies	Eestimated group size	Ttype	RL (mMax)	RL (rRange)	Median [IQR] whistles per secondecond Median [IQR] (full 30 min period)	# cChangepoints (mean, pre-exposure) - mean - pre-exposure	C# changepoints (- mean, - exposure)	#C changepoints - (variance - pre-exposure)	C# changepoints (- variance - exposure)
2019_01	Db	260	simMFAS	147 dB re 1µPa RMS	140-147 dB re 1µPa RMS	6.96 [6.39]	26	39	5	1
2019_02	Dd	350	control	n/a	n/a	4.80 [5.19]	37	29	0	5
2019_04	Db	200	control	n/a	n/a	0.25 [0.77]	2	0	4	2
2019_06	Db	45	control	n/a	n/a	0.32 [1.38]	5	0	4	9
2019_07	Db	300	simMFAS	154 dB re 1µPa RMS	150-154 dB re 1µPa RMS	3.11 [3.81]	36	20	3	3
2019_08	Db	250	simMFAS	142 dB re 1µPa RMS	131-142 dB re 1µPa RMS	1.50 [3.91]	30	12	4	4
2019_09	Dd	250	control	n/a	n/a	4.21 [5.38]	46	44	2	4
2019_10	Dd	30	simMFAS	149 dB re 1µPa RMS	146-149 dB re 1µPa RMS	0.091 [0.38]	0	0	3	7
2021_01	Db	150	control	n/a	n/a	3.14 [4.47]	3	51	1	3
2021_02	Db	200	control	n/a	n/a	4.68 [3.89]	18	29	0	4
2021_03	Dd	150	control	n/a	n/a	0.99 [2.01]	11	0	2	0
2021_04	Db	150	control	n/a	n/a	4.66 [4.20]	27	37	4	2
2021_05	Dd	250	control	n/a	n/a	0.36 [1.87]	0	5	8	4
2021_08	Db	30	simMFAS	153 dB re 1µPa RMS	145-153 dB re 1µPa RMS	14.13 [7.85]	50	49	2	4
2021_09	Db	200	simMFAS	157 dB re 1µPa RMS	152-157 dB re 1µPa RMS	1.17 [2.17]	3	6	2	2
2021_10	Db	300	simMFAS	159 dB re 1µPa RMS	150-159 dB re 1µPa RMS	15.34 [9.16]	42	61	3	2
2021_11	Db	10	simMFAS	153 dB re 1µPa RMS	150-153 dB re 1µPa RMS	0.0029 [0.063]	0	0	0	6
2021_12	Dd	150	simMFAS	152 dB re 1µPa RMS	149-152 dB re 1µPa RMS	2.85 [3.07]	7	24	4	6
2021_13	Dd	200	simMFAS	147 dB re 1µPa RMS	139-147 dB re 1µPa RMS	2.21 [4.73]	2	2	9	4

Table 1. Summary of each controlled exposure experiment including controls (no sound emitted) and 'simMFAS' (playback of simulated mid-frequency active sonar). CEE -ID contains denotes the year and the CEE number. Species abbreviations are Db for *D. bairdii* and Dd for *D. delphis* and Subspecies abbreviations are Db for *D. d. bairdii* and Dd for *D. d. delphis*, and the estimated group size is taken from shore observations. Received sound levels (RLs) are reported fromby Durban et al. 2022 [29].. MedianThe median and interquartile range (IQR, 25-75th percentiles) are given for the raw whistle counts detections per secondnd of the entire 30-minutemin CEE. Changepoints were calculated on for both the mean whistle count and the variance in whistle count, separately for the pre-exposure and exposure periods. Note that "exposures" in controls were quiet periods for comparisoncompared to sound exposure in MFAS trials.

Formatted: Font: Italic
Formatted: Font: Italic
Formatted: Font: Italic
Formatted: Font: Italic

2. Baseline Changepoint Analysis

The baseline changepoint analysis revealed that mean detected whistle counts over the 30-minutemin control sampling periods changed once every minutemin, and variance in detected whistle countcounts changed once every 3.5 minutemin.s in control conditions.

Formatted: Font: 14 pt, Not Italic
Formatted: Font: 14 pt
Formatted: Indent: First line: 0.5"

B. Assessing the impact of disturbance on whistle behavior

A total of 10 MFAS CEEs were conducted – - eightseven of which included Delphinus bairdii,long-beaked common dolphins and two three of which included Delphinus delphisshort-beaked common dolphins. The calculated average received level across all experiments was 151 dB re 1µPa RMS (range 142-159 dB re 1µPa RMS, Table 1). The average group size for MFAS

Formatted: Font: 16 pt
Formatted: Indent: First line: 0.5"
Formatted: Font: Not Italic
Formatted: Font: Not Italic
Formatted: Font: Not Italic

CEEs was ~173 individuals (range 10-300, Table 1). For the changepoint analysis, all 10 MFAS experiments were included. However, for the assessment of changes in whistle behaviors across different time scales, we excluded CEE 2021_11 (conducted with long-beaked common dolphins) had to be excluded because the overall lack of detected whistles could not be successfully modeleddue to the lack of detected whistles, which prohibited its inclusion in the modeling. Consequently, the modeling results include nine controls and nine MFAS experiments.

Formatted: Font: Not Italic

1. Number of changepoints in pre-exposure vs exposure

Formatted: Font: 14 pt, Not Italic

Formatted: Font: 14 pt

Formatted: Indent: First line: 0.5"

Changepoint analysis was run for all control (9) and MFAS (10) experiments. The number of changepoints detected in both the mean and variance of whistle count during the pre-exposure and exposure periods areis presented in Table 1 for both controls and MFAS experiments. There was no significant increase or decrease in the mean and variance of detected whistle counts following MFAS exposure when compared to the natural variance present during control conditions. The difference (Δ) in change points in variance in of whistle detections count between the pre-exposure and exposure period did not differ significantly between controls and MFAS CEEs (controls: $M = 3.1$, $SD = 1.4$; MFAS: $M = 2.5$, $SD = 2.3$; $t(17) = 0.69$, $p = 0.5$). The same was true when comparing the mean whistle count between both experimental types (controls: $M = 11.3$, $SD = 14.2$; MFAS, $M = 8.7$, $SD = 8.5$; $t(17) = 0.5$, $p = 0.63$).

2. Impact of MFAS exposure on whistle count: 10-minutemin time scale

Formatted: Font: 14 pt, Not Italic

Formatted: Font: 14 pt, Not Italic

Formatted: Font: 14 pt

Formatted: Indent: First line: 0.5"

Whistle counts detections did not change between the pre-exposure and exposure period during MFAS experiments at the 10-min time scale. At the 10-minute time scale, theThe

preferred model was the simplest model with the absolute value of the change in median whistle count as a function of only CEE type (either MFAS or control). There was no significant effect of CEE type on the change in median whistle count detections (negative binomial GLMM, n = 18, p = 0.8). The full model (ΔAIC 5.4) indicated that no proposed predictor variables (CEE type, CEE number, speciessubspecies, buoy distance, or group size) had a significant effect on the change in median whistle count between the pre-exposure and exposure periods for both MFAS and control CEEs (negative binomial GLMM, n = 19: P > 0.05 for all variables, Ttable 2).

Table 2. Overview of GLMMs used at three time-scalestime scales -– 10- minutemins, 20- seconds, and 5- seconds.

Cconditional model	Zzero-inflation model	Ddistribution	ΔAIC	Ddegrees of freedom	Ddispersion (σ^2)
10-minutemin scale					
abs(*wrperiodDiff*) ~ *ceeType*	n/a	nbinom2	0	3	5.44
abs(*periodDiff*wrDiff) ~ *ceeType* + (1 | *ceeNum*)	n/a	nbinom2	2	4	1.04
abs(*periodDiff*wrDiff) ~ *ceeType* + (1 | *ceeNum*) + *subS*species + *buoyDist* + *groupSize*	n/a	nbinom2	5.4	7	1.48
20-second scale					
pingDdiff ~ *ceeType* + *medWhistexpMed*	~*medWhist*expMed	gaussian	0	6	7.1
*pingDiff*diff ~ *ceeType*	~*medWhist*expMed	gaussian	5.1	5	7.23
*pingDiff*diff ~ *ceeType* + *subSpecies*species + *groupSize* + *buoyDist* + *medWhist*expMed	~*medWhist*expMed	gaussian	5.3	9	7.09
*pingDiff*diff ~ *ceeType* + (1 | *ceeNum*)	~*medWhist*expMed	gaussian	7.1	6	7.23
*pingDiff*diff ~ *ceeType*	none	gaussian	28.3	3	6.71
5-second scale					
*pingDiff*diff ~ *ceeType* + *groupSize* + *medWhist*expMed + ar1(*times* + 0 | *ceeNum*)	~*medWhist*expMed	gaussian	0	9	10.1

pingDiffdiff ~ ceeType + (1 | ceeNum) + subSpeciesspecies + groupSize + buoyDist + medWhistexpMed + ar1(times + 0 | ceeNum)	~medWhistexpMed	gaussian	2.6	12	10.1
pingDiffdiff ~ ceeType + ar1(times + 0 | ceeNum)	~medWhistexpMed	gaussian	13.8	7	9.83
pingDiffdiff ~ ceeType + (1 | ceeNum) + ar1(times + 0 | ceeNum)	~medWhistexpMed	gaussian	15.8	8	9.83
pingDiffdiff ~ ceeType	none	gaussian	207.8	3	10.9

Model formulas (conditional and zero-inflation if included) are listed for each time scale of analysis, and within each time scale are given in ascending order of ΔAIC relative to the best model (ΔAIC = 0).

3. Characterize the impact of MFAS exposure on whistle count: 20-second time scale

Whistle counts detections did not differ significantly over the 20-second time windows surrounding each ping. The preferred model at the 20 second20-s scale included only predictor variables for CEE type and median whistle count (Ttable 2). The results of this model showed that CEE ceetType did not have a significant effect on changes in whistle count in the 20-seconds20 s after each ping (GLMM, n=18, slope = 0.47, SE = 0.27, P > 0.05, Ttable 2b), but that the baseline median whistle count for that experimental period was a significant predictor for the change in whistle count following a ping or ghost pingcontrol treatment (GLMM, n = 19, slope = 0.76, SE = 0.028, p = 0.0075).

4. Characterize the impact of MFAS exposure on whistle count: 5-second time scale

The preferred model at the 5 second5-s scale included the temporal autocorrelation structure and three explanatory variables, CEE type, group size, and median whistle count, all of

Formatted: Font: Italic
Formatted: Font: Italic
Formatted: Font: Italic
Formatted: Font: Italic
Formatted: Don't add space between paragraphs of the same style, Line spacing: Double
Formatted: Font: Italic
Formatted: Font: Italic
Formatted: Font: Italic
Formatted: Don't add space between paragraphs of the same style, Line spacing: Double
Formatted: Font: Italic
Formatted: Font: Italic
Formatted: Font: Italic
Formatted: Don't add space between paragraphs of the same style, Line spacing: Double
Formatted: Font: Italic
Formatted: Font: Italic
Formatted: Font: Italic
Formatted: Font: Italic
Formatted: Don't add space between paragraphs of the same style, Line spacing: Double
Formatted: Font: Italic
Formatted: Font: Not Bold
Formatted: Font: Not Bold
Formatted: Font: 14 pt, Not Italic
Formatted: Font: 14 pt
Formatted: Indent: First line: 0.5"
Formatted: Font: 14 pt, Not Italic
Formatted: Font: 14 pt
Formatted: Indent: First line: 0.5"

which had a significant effect on the change in whistle count in the 5 seconds immediately following a ping compared to the 5 seconds immediately before a ping. When accounting for all other variables, the magnitude of the change in whistle count in the 5 seconds following a truean actual MFAS ping was 1.4 timesx greater than any change in whistle count following ghost pings in control CEEs control treatments (GLMM, n=18, slope = 1.43, SE = 0.47, p = 0.002, Ttable 2c). The results of the CEE by CEE analysis (S2 AppendixS1 File) showed that in four five of the nine MFAS experiments in which sound was broadcasted, detected whistle counts were significantly elevated dolphins exhibited a significant elevation in whistle production\ in the 5-seconds5 s after each ping for throughout the entire 10-minute exposure period, while in three of the nine MFAS exposures, animals showed an initial response to the first ping, with vocal responses slowly abating over the course of the experiment (plots of all raw whistles are provided in S2 FigS3 Appendix). The effect was particularly pronounced (outside the 75th percentile; Appendix S2 Fig. S2.1S3 Fig) in the first ping of six6 of the MFAS CEEs; whistle activity increased in the 5 seconds following the first MFAS ping up to 15 times the whistle count in the 5 seconds before the first MFAS ping (mean of all MFAS CEEs 3.9, SD 5.2), compared to increases of only up to 1.4 times (mean 0.46, SD 0.88) at the first ghost ping of controlsat the start of control treatments (Fig. 5, Appendix S2.1S3 Fig). Additionally, group size and median whistle count for the exposure period were found to be significant predictor variables. Larger groups showed largermore extensive changes in whistle count following pings and ghost pingscontrol treatments (GLMM, n=18, slope = 0.007, SE = 0.003, p = 0.008, Ttable 2c), and when the median background whistle count was higher, so too were the changes following pings or and ghost pingscontrol treatments (GLMM, n=18, slope = 0.25, SE = 0.048, p = < 0.005, Ttable 2).

Figure 5. (A) Spectrogram example of 5 seconds before and 5 seconds after the first ping for MFAS CEE 2021_08, illustrating illustrates the largesignificant increase in whistle count immediately following the cessation of the ping. The grey area denotes where whistle detections were excluded due to the recorder distance exceeding 1.6 km. **.** FocalThe focal group was comprised of approximately 30 long-beaked common dolphins. The MFAS signal can be seen between 3 and 4 kHz. (B) Boxplot of the change in whistle count from the 5 seconds before to the 5 seconds following each of the 24 pings for CEE 2021_08. Boxplot shows median, 25th, and 75th percentiles, with raw whistle count changes as open gray circles. The change following the first ping is shown as a red star.

DiscussionDiscussionConclusions

We present a hierarchical approach to quantifying the vocal response of large groups of common dolphins to Navy Sonar and find that the most pronounced acoustic response occurs within the 5-seconds following each ping during MFAS exposure. As in previous observational studies evaluating delphinid acoustic behavior (14), determining a singular behavioral response to MFAS presents several challenges. Multiple factors – including rapid changes in the behavioral state over the course ofthroughout the experiment and variation in group size and composition – make it difficult to assess whether ascribe changes in vocal behavior are due to disturbance or versus natural variability. To better understand typical acoustic variation among common dolphins, we assessed vocal behavior during control conditionsWe assessed vocal behavior during control conditions to understand typical acoustic variation among common dolphins and found. We found that they dolphins exhibited natural vocal state changes (identified

Commented [SF3]: I think ok to still call this Discussion and above just Results. The example in the formatting website has 'Results and discussion' but then the balloon on the side lists the possible sections as Results, Discussion

Formatted: Font: 18 pt

Formatted: Font: 18 pt

Formatted: Indent: First line: 0.25"

by the changepoint analysis) in whistle production approximately once every minutemin. This rapid acoustic state switching informed our analytical approach, which utilized a range of temporal windows to test for changes in whistle count (5 s – 10 min10 minutes - 5 seconds) before and after MFAS exposure. Across the larger longer time windows selected,selected; we did not detect a changeshift in whistle behavior that was attributable to MFAS. However, CEE type did have a significant effect onsignificantly affected the change in whistle count in the 5-seconds immediately following a ping compared to the 5-seconds5 s following a ping compared to the 5 s immediately before a ping.

The initial selection of the time time-periodperiod over which behavior was assessed in response to Navy sonar (10 -minutes) for this project was influenced by multiple factors, including the flight endurance of the drone used for calculating animal distance to our recording buoys (34), our ability to consistently track large groups of fast-moving dolphins, and other previous BRS methods studies using MFAS (*e.g.*, 18, 37643, 21]). Many of the aforementioned aforementioned constraints are imposed by the logistics of field workfieldwork. An informed approach to identifyidentifying behavioral responses to anthropogenic disturbances sources also requires some prior knowledge of the timing of behavioral state switching in the study species. For example, previous work with blue whales (*Balaenoptera musculus*) evaluated several behavioral metrics (*e.g.,* maximum depth, dive time, ascent/descent rate) in responses to simulated MFAS exposure over a 30-minute time window [376](43). This exposure duration (which included a 30-minutemin pre-exposure period) was adequate to capture a behavioral change given the typical duration of their dive cycles ((5-8 minutemins) [387], 44). In contrast, beaked whales are known to exhibit extremelyincredibly long, deep foraging dives that often last

Formatted: Indent: First line: 0.25"

over an hour, followed by long periods of recovery [(*e.g.*, 39845,403946). Consequently, studies focused on direct measurements of behavioral response by Cuvier's beaked whales to MFAS extended their pre-exposure baseline period up to 9.3 hours and to perform sound exposure during foraging dives and evaluated their response to sonar for up to 1.7 hours after the exposure period [410](47).

While the 10-minutemin exposure period seemed appropriate given the fast-paced lifestyle of common dolphins, neither the aggregate model nor the individual assessment of each CEE detected a change in acoustic behavior that could be ascribed to sonar exposure at this time scale. Even when we explored variation in whistle production during the 20-seconds surrounding each ping, the experimental period was not a significant predictor of changes in whistle count in either the MFAS or control experiments, as vocal state switching often occurs within a 40 second20-s surrounding each ping, the experimental period was not a significant predictor of changes in whistle count in either the MFAS or control experiments, as vocal state switching often occurs within a 40-s time window under natural baseline conditions. While the impacts of sonar may be evident over the 10-minnute exposure period for other behavioral metrics (*i.e.*, changes in behavioral state, group composition, diving behavior), our analysis reveals that changes in acoustic behavior are limited to an extremely narrow time-windowtime window in these two speciessubspecies.

Formatted: Indent: First line: 0.25"

Formatted: Font: Italic

It was only at the 5-second time scale surrounding each ping that we observed dolphins exhibiting an acute acoustic response, which often included a rapid increase in whistle production relative to the 5 seconds immediately prior tobefore sound exposure. On average,

Formatted: Indent: First line: 0.25"

dolphins increased their whistle count 4four times the average count in the 5 seconds preceding the first ping of the exposure. In one MFAS experiment, dolphins increased their whistle production 15 times compared to the whistle count in the 5 seconds immediately before the first ping (S3 Fig) (Appendix S2.1). This elevated vocal response following the first ping of the exposure was seen in six of the nine MFAS exposures where whistles were present.

Elevations in whistle counts detections did not occur during the sonar signal transmission itself – which lasted for 1.6 seconds. Rather, the elevated increased vocal production occurred once the signal had been transmitted, often abating within ~ 10 seconds. The lack of whistle production occurring during the sonar transmission may be a tactic for reducing acoustic interference and masking, which has been shown to impact the detection, discrimination, and localization of relevant signals [412](48). If the interfering signal is predictable (as is the case in our experiment), then animals should be able to adjust the timing of sound production to limit communication to periods in which noise is reduced [(*e.g.*, 4214-4458-50]). The ability of dolphins to learn the timing of intermittent noise has previously been demonstrated by Finneran et al., 2023 [421](51), who showed that individuals can modify their hearing sensitivity prior tobefore the onset of predictably timed impulses, presumably to mitigate negative adverse auditory impacts. Surprisingly, little is known about their capacity to modifyalter the timing of vocal production in response to interfering signals under natural baseline conditions.

The sudden increase in vocal behavior following the first ping could be an example of the amplification of the behavior of group members through recruitment or reinforcement (*i.e.*, positive feedback), [45652]). In this scenario, one dolphin may whistle in response to a

Formatted: Indent: First line: 0.25"

Formatted: Indent: First line: 0.25"

surprising, salient stimuli and others , and others may follow suit. As this recruitment response continues, the number of dolphins producing whistles will increase s further, and information is spread rapidly throughout the group [(45763]). In such cases, a few key individuals could catalyze the collective behavior of the rest of the group. While it would be difficult to evaluate this process using acoustic data alone, concurrent video data collected from the associated drone flights is currently being assessed to explore the spatial movement patterns of the group and identify those individuals that successfully initiate changes in group movement. Alternatively, it the rapid increase in vocal behavior following each ping could be an indicator that multiple animals are exchangingindicate that multiple animals exchange whistles to contact their closest social partners within the group in the presence of an unknown stimulus. This could be expected given the role of whistles in group cohesion and coordination [(2,48754]) .

Surprisingly, in the playbacks when dolphins showed a significant increase in vocal behavior in the 5-sec following the first ping (6 of 9 CEEs, S3 Fig), the severity of their acoustic response did not abate over the course of the exposure Whether animals continued to show an elevated acoustic response immediately following each ping varied between MFAS exposures. In four of the nine experiments in which sound was broadcasted, dolphins exhibited a significant elevation in whistle production in the 5-seconds after each ping throughout the entire 10-minute exposure period (S22 AppendixFig). This suggests that in these cases, dolphins did not habituate to successive pings (*i.e.*, show a progressive decrease in the amplitude of a vocal behavioral response after repeated exposure). This observation is surprising given that However, in three of the nine MFAS exposures, animals showed an initial response to the first ping, with vocal responses slowly abating over the course of the experiment (S2 Appendix). Thethe population of

Formatted: Font color: Black

Formatted: Indent: First line: 0.25"

Formatted: Not Highlight

Formatted: Not Highlight

dolphins tested in this study likely live a large portion of their lives in areas that are regularly ensonified by Navy sonar, and thus may. Continued work is needed to confirm these initial findings; for example, future studies exhibit an attenuated response when compared to individuals not regularly exposed to MFAS. Future work could compare the responsiveness of animalsanimals' responsiveness in the Southern California Bight to nearby populations that occur in areas where Navy sonar is not regularly present (*e.g.*, Monterey Bay). Considering whether dolphins may be learning to modify their vocal behavior in response to repeated noise exposure may provide foundational evidence to support using vocal rates as a measure of sensitization or habituation to anthropogenic stimuli, as has been done in terrestrial species [as in (45985,50,4596]).

Formatted: Not Highlight

Formatted: Not Highlight

Formatted: Font color: Black

Future management decisions mitigating the impact of sonar on oceanic delphinids should consider our reported results of clear responses during CEEs when analyses were conducted at the appropriate temporal resolution . With respect toConcerning the most recent methodology for assessing the relative response severity for free-ranging marine mammals to acoustic disturbance [(51057]) – had the acoustic response of animals to MFAS been pooled across the 10-minutemin time window – common dolphins likely would have been assigned a behavioral response severity score of 0 (no response detected). However, when evaluating vocal behavior across a shorter time-time 5-second time window, this species' response would be elevated to a category 3 severityanimal's response would be elevated to a category three severity, which includes an increase in possible contact or alarm calls [(51057]). Ultimately, continued work with this (and other closely related) species should also consider how observed behavioral responses vary with respect to other contextual parameters, including behavioral state, group composition (*e.g.*,

Formatted: Indent: First line: 0.25"

presence or absence of calves), seasonality, and environmental covariates. Concurrent efforts from this project aim to integrate passive acoustics with other remotely sensed datasets (*i.e.*, shore-based group tracking, and aerial photogrammetry) to identify group-level behavioral changes and quantify exposure-response relationships [498](58). Paired with energetic modeling methods [(*e.g.*, 2731,53259,54360]), these data can be used to link these observed short-term behavioral responses to long-term fitness outcomes in this species and inform the implementation of effective mitigation strategies.

Our study has some limitations that can be addressed in future work. Given the close phylogenetic relationship between short-beaked (*Delphinus delphis*) and long-beaked common dolphins (*Delphinus bairdii*), [(55461,56562]), the basic description of baseline vocal behavior (*i.e.*, whistle count data) was combined across speciessubspecies in our analysis. However, recent work by Oswald et al. discovered unique species-specific whistle frequency contours in short-beaked common dolphins both subspecies, *D. delphis and D. bairdii* and suggestedsuggesting that these distinctive acoustic signals could help facilitate recognition between these two *Delphinus* speciessubspecies [576](38). An increase in sample size for both long-beaked and short-beaked common dolphins would provide a more detailed understanding of their baseline vocal behavior and allow for the explorationenable the exploration of whether each speciessubspecies shows a differential or similar response in whistle typewhistle-type usage to MFAS. Additionally, photo-identificationphoto identification of individuals within these large, ephemeral groups is extremely difficultchallenging. Consequently, it is unknown whether individual dolphins around Catalina Island were exposed more than once to the experimental treatmentwhether individual dolphins around Catalina Island were exposed more than once to the

Formatted: Indent: First line: 0.25"

experimental treatment is unknown. Future research could direct effort towardsefforts toward other oceanic delphinids with smaller group sizes sizes where photo-identification is feasible and reliable (*e.g.*, bottlenose or rough-toothed dolphins dolphins).

In any behavioral experiment, the response of the individual or group that is tested should be measured and interpreted over a time-windowtime window that is informed by their natural behavior. We suggest that future work with other oceanic delphinid species explore baseline vocal rates a-priori and use information on vocal state-switching to inform the analysis time-windowtime window over which behavioral responses are measured. Given these species' susceptibility to frequent MFAS exposure in Navy operational areas, it is of particular interest to evaluate how repeated exposure influences responsesanimals' susceptibility to frequent MFAS exposure in Navy operational areas, evaluating how repeated exposure influences responses is of particular interest. Future work should continue to explore keycritical factors that are likely to influence the probability of response among these large groups, including their behavioral state and their likely to affect the probability of response among these large groups, including their behavioral state and proximity to the sound source and received level at the onset of exposure.

Formatted: Indent: First line: 0.25"

AcknowledgementsAcknowledgments

Formatted: Font: 18 pt

Formatted: Font: 18 pt

Funding for this project was provided by the U.S. Navy's Office of Naval Research (Award Numbers N000141713132, N0001418IP-00021, N000141712887, N000141912572). Animal observations, octocopter flights over dolphins, close approaches and CEEs were conducted under NMFS permit, and CEEs were conducted under NMFS permits 19116 and 19091. Additionally, all activities reported in this study were reviewed and approved by the Institutional Animal Care

and Use Committee (IACUC Protocol No: CRC-2021-AUP-06, CRC-2021-AUP-08). We thank the *M/V Magician* and Captain Carl Mayhugh for support in the field, as well as the University of Southern California's Wrigley Institute for their hospitality throughout the duration of and the University of Southern California's Dornsife Wrigley Institute for Environment and Sustainability for their hospitality throughout this project. This project would not have been possible without the dedicated contribution of all members of the Tagless-BRS team. Thank you to Stacy DeRuiter for extensive advice on statistical modeling and analysis. Additionally, we appreciate the thoughtful feedback provided by Dr. Ari Friedlaender, Dr. Colleen Reichmuth, Dr. Peter Cook, and Dr. John Durban during analysis and manuscript preparation. We also thank Ryan Jones, who created the line drawings for Fig. 1.

Formatted: Font: Italic

References

1. Tyack PL. Acoustic cCommunication uUnder the Ssea. In: Hopp SL, Owren MJ, Evans CS, editors. Animal aAcoustic cCommunication. Springer Berlin / Heidelberg: Springer; 1998.

2. Janik VM. Acoustic cCommunication in dDelphinids. Adv Study Behav. 2009; ;40: 123–157.

3. Lammers MO, Oswald JN. Analyzing the acoustic communication of dolphins. In: Herzing DL, Johnson CM, editors. Dolphin communication and cognition: Past, present, and future. Cambridge: MIT Press; 2015. pp. 107-130.

4. Erbe C, Dunlop R, Dolman S. Effects of nNoise on mMarine mMammals. In: Slabbekoorn H, Dooling R, Popper A, Fay R, editors. Effects of aAnthropogenic nNoise on aAnimals. New York : Springer; 2018.

5. Williams R, Wright AJ, Ashe E, Blight LK, Bruintjes R, Canessa R, et al. Impacts of anthropogenic noise on marine life: Publication patterns, new discoveries, and future directions in research and management. Ocean Coast Manag. 2015;115: 17–24.

6. Southall BL, Finneran JJ, Reichmuth C, Nachtigall PE, Ketten DR, Bowles AE, et al. Marine mammal noise exposure criteria: Updated scientific recommendations for residual hearing effects. Aquat Mamm. 2019;45(2): 125–232.

7. Hatch LT, Wahle CM, Gedamke Jason, Harrison Jolie, Laws Benjamin, Moore SE, et al. Can you hear me here? Managing acoustic habitat in US waters. Endanger Species Res. 2016;30(1): 171–86.

8. Tyack PL, Janik VM. Effects of nNoise on aAcoustic sSignal pProduction in mMarine mMammals. In: Brumm H, editor. Animal cCommunication and nNoise Animal Signals and Communication. New York: Springer; 2013. pp. 251-271.

9. Nowacek DP, Thorne LH, Johnston DW, Tyack PL. Responses of cetaceans to anthropogenic noise. Mamm Rev. 2007;37(2): 81–115.

10. Hastie G, Merchant ND, Götz T, Russell DJF, Thompson P, Janik VM. Effects of impulsive noise on marine mammals: Iinvestigating range-dependent risk. Ecol Appl. . 2019;29: e01906.

11. Harris CM, Sadykova D, DeRuiter SL, Tyack PL, Miller PJO, Kvadsheim PH, et al. Dose response severity functions for acoustic disturbance in cetaceans using recurrent event survival analysis. Ecosphere. 2015;6(11): 1–14.

12. Southall EBL, Finneran JJ, Reichmuth C, Nachtigall PE, Ketten DR, Bowles AE, et al. Marine mammal noise exposure criteria: Updated scientific recommendations for residual hearing effects. Aquat Mamm. 2019;45(2): 125–232.

Formatted: Font: 12 pt

13. D'Amico A, Pittenger R. A brief history of active sonar. Aquat Mamm. 2009 Dec;35(4): 426–34.

14. Henderson EE, Smith MH, Gassmann M, Wiggins SM, Douglas AB, Hildebrand JA. Delphinid behavioral responses to incidental mid-frequency active sonar. J Acoust Soc Am. 2014;136(4): 2003–2014. Available from: http://dx.doi.org/10.1121/1.4895681

156. Oswald JN, Rankin S, Barlow J. The effect of recording and analysis bandwidth on acoustic identification of delphinid species. J Acoust Soc Am. 2004 Nov 1;116(5): 3178–3185.

16. Filadelfo R, Mintz J, Michlovich E, D'Amico A, Tyack PL, Ketten DR. Correlating military sonar use with beaked whale mass strandings: What do the historical data show? Aquat Mamm. 2009;35(4): 435–444.

17. Guan S, Brookens T. An overview of research efforts to understand the effects of underwater sound on cetaceans. Vol. 2, Water Biology and Security. 2023;2: 100141.

1821. Southall BL, DeRuiter SL, Friedlaender A, Stimpert AK, Goldbogen JA, Hazen E, et al. Behavioral responses of individual blue whales (*Balaenoptera musculus*) to mid-frequency military sonar. Journal of Experimental BiologyJ Exp Biol. 2019;222: (5).

1922. Sivle LD, Kvadsheim PH, Fahlman A, Lam FPA, Tyack PL, Miller PJO, et al. Changes in dive behavior during naval sonar exposure in killer whales, long-finned pilot whales, and sperm whales. Frontiers in PhysiologyFront Physiol. 2012; 11;3: 400. Available from: www.frontiersin.org

20. Deruiter SL, Boyd IL, Claridge DE, Clark CW, Gagnon C, Southall BL, et al. Delphinid whistle production and call matching during playback of simulated military sonar. Mar Mamm Sci. 2013;29(2): E46-E59.

21. Ioannou CC, Laskowski KL. A multi-scale review of the dynamics of collective behaviour: from rapid responses to ontogeny and evolution. Philos Trans R Soc Lond, B. 2023;10(378): 20220059.

22. Durbach IN, Harris CM, Martin C, Helble TA, Henderson EE, Ierley G, et al. Changes in the mMovement and cCalling bBehavior of mMinke wWhales (*Balaenoptera acutorostrata*) in Response to Navy Training. Front Mar Sci. 2021;8: 660122.

23. Rendell LE, Gordon JCD. Vocal response of long-finned pilot whales (*Globicephala melas*) to military sonar in the Ligurian Sea. Mar Mamm Sci. 1999;15(1): 198–204.

24. Miller PJO, Antunes RN, Wensveen PJ, Samarra FIP, Catarina Alves A, Tyack PL, et al. Dose-response relationships for the onset of avoidance of sonar by free-ranging killer whales. J Acoust Soc Am. 2014;135(2): 975–93.

25. DeRuiter SL, Boyd IL, Claridge DE, Clark CW, Gagnon C, Southall BL, et alTyack PL. Delphinid whistle production and call matching during playback of simulated military sonar. Mar Mamm Sci. 2013;2: 46-59.

26. Lara G, Bou-Cabo M, Llorens S, Miralles R, Espinosa V. Acoustical behavior of delphinid whistles in the presence of an underwater explosion event in the Mediterranean coastal waters of spain. J Mar Sci. 2023;11(4): 780.

27. Keen KA, Beltran RS, Pirotta E, Costa DP. Emerging themes in population consequences of disturbance models. Proc R Soc B. 2021;288(1957): 20210325.

28. King SL, Connor RC, Montgomery SH. Social and vocal complexity in bottlenose dolphins. Trends Neursci. 2022; 45: 881-883.

29. Durban JW, Southall BL, Calambokidis J, Casey C, Fearnbach H, Joyce TW, et al. Integrating remote sensing methods during controlled exposure experiments to quantify group responses of dolphins to navy sonar. Mar Pollut Bull. 2022;174: 113194.

30. Visser, F. Moving in concert: Social and migratory behaviour of dolphins and whales in the North Atlantic Ocean. PhD dissertation, University of Amsterdam. 2014.

31. Hager CA. Assessment of the performance of the near-bottom hydrophones of the US Navy Southern California offshore range in detecting, localizing and reconstructing 10-20KHZ odontocete whistles. PhD dissertation, Naval Postgraduate School. 2008.

32. Gillespie D, Mellinger DK, Gordon J, McLaren D, Redmond P, McHugh R, et al. PAMGUARD: Semiautomated, open-source software for real- time acoustic detection and localization of cetaceans. J Acoust Soc Am. 2009;125(4): 2547–2547.

Formatted: Indent: Left: 0", First line: 0"

332. Sakai T, Barlow, J, Griffiths, E, Oswald, J, Schmidt, V. et al. R Package 'PAMpal'. 2022. PAMmisc: Miscellaneous functions for passive acoustic analysis. 2023. https://CRAN.R-project.org/package=PAMmisc

Field Code Changed

34. R Core Team. R: A Language and Environment for Statistical Computing. R Foundation for Statistical Computing, Vienna, Austria. 2023. https://www.R-project.org/

33. Gillespie D, Mellinger DK, Gordon J, David McLaren, et al. PAMGUARD: Semiautomated, open-source software for real- time acoustic detection and localization of cetaceans. J Acoust Soc Am. 2009;125(4): 2547–2547.

354. Killick R, Eckley I. changepoint: An R package for changepoint analysis. J Stat Softw. 2014;58(3): 1-9.

365. Brooks ME, Kristensen K, Van Benthem KJ, Magnusson A, Berg CW, Nielsen A, et al. glmmTMB balances speed and flexibility among packages for zero-inflated generalized linear mixed modeling. The R Jjournal. 2017;9(2): 378-400.

367. Goldbogen JA, Southall BL, DeRuiter SL, Calambokidis J, Friedlaender AS, Hazen EL, et al. Blue whales respond to simulated mid-frequency military sonar. Proc R Soc B. 2013;280: 20130657.

387. Croll DA, Acevedo-Gutierrez A, Tershy BR, Urban-Ramırez J. The diving behavior of blue and fin whales: Iis dive duration shorter than expected based on oxygen stores? Comp Biochem Physiol, Part A Mol Integ. 2001;129(4): 797-809.

398. Baird RW, Webster DL, Schorr GS, McSweeney DJ, Barlow J. Diel variation in beaked whale diving behavior. Mar Mamm Sci. 2008;24(3): 630–642.

4039. Shearer JM, Quick NJ, Cioffi WR, Baird RW, Webster D, Foley HJ, et al. Diving behaviour of Cuvier's beaked whales (*Ziphius cavirostris*) off Cape Hatteras, North Carolina. R Soc Open Sci. 2019;1(6) 26(2): 181728.

410. Deruiter SL, Southall BL, Calambokidis J, Zimmer WMX, Sadykova D, Falcone EA, et al. First direct measurements of behavioural responses by Cuvier's beaked whales to mid-frequency active sonar. Bio Lett. 2013;9: 20130223.

421. Finneran JJ, Schlundt CE, Bowman V, Jenkins K. Dolphins reduce hearing sensitivity in anticipation of repetitive impulsive noise exposures. J Acoust Soc Am. 2023;153(6): 3372.

432. Egnor SR, Wickelgren JG, Hauser MD. Tracking silence: adjusting vocal production to avoid acoustic interference. J Comp Physiol. 2007;193: 477-483.

443. Kiai A, Clemens J, Kössl M, Poeppel D, Hechavarría Jet al. Flexible control of vocal timing in bats enables escape from acoustic interference. bioRxiv. 2023: 2023-05.

Formatted: Font: Italic

454. Munoz-Santos I, Ríos-Chelén AA. Vermilion flycatchers avoid singing during sudden peaks of anthropogenic noise. aActa ethologica. 2022;26(3): 201-210.

465. Bonabeau E, Theraulaz G, Deneubourg J.L, Aron S, Camazine S. Self-organization in social insects. Trends Ecol Evol. 1997;12(188).

476. Sumpter DJT. The principles of collective animal behaviour. Philos Trans R Soc Lond, B. 2005;361: 5–22.

487. Caldwell MC, Caldwell DK. Vocalization of naive captive dolphins in small groups. Science. 1968;159(3819): 1121-1123.

498. Francis CD, Ortega CP, Cruz A. Noise pollution changes avian communities and species interactions. Curr Biol.. 2009;19(16):1415–1419.

5049. Brown CL, Hardy AR, Barber JR, Fristrup KM, Crooks KR, Angeloni LM.et al. The effect of human activities and their associated noise on ungulate behavior. PloS One. 2012;10;(7): e40505.

510. Southall BL, Tollit D, Amaral J, Clark CW, Ellison WT. Managing human activity and marine mammals: A biologically based, relativistic risk assessment framework. Front Mar Sci. 2023;10.

521. Harris CM, Thomas L, Falcone EA, Hildebrand J, Houser D, Kvadsheim PH, et al. Marine mammals and sonar: Dose- response studies, the risk- disturbance hypothesis and the role of exposure context. J Appl Ecol. 2018;55:396-404.

53.2. Harwood J, King S, Booth C, Donovan C, Schick RS, Thomas L, et al. Understanding the population consequences of acoustic disturbance for marine mammals. In Popper AN, Hawkins A, editors. The effects of noise on aquatic life II. New York: Springer; 2016. pp. 417-423.

Formatted: Don't add space between paragraphs of the same style

543. McHuron EA, Adamczak S, Costa DP, Booth C. Estimating reproductive costs in marine mammal bioenergetic models: aA review of current knowledge and data availability. Conserv Physiol. 2023;11(1): coac080.

554. Natoli A, Cañadas A, Peddemors VM, Aguilar A, Vaquero C, Fernandez-Piqueras P, et al. Phylogeography and alpha taxonomy of the common dolphin (*Delphinus* sp.). J Evol Biol. 2006;19(3): 943-954.

565. McGgowen MR, Tsagkogeorga G, Álvarez-Carretero S, Dos Reis M, Truebig M, Deaville R, et al.Reis M Dos, et al. Phylogenomic resolution of the cetacean tree of life using target sequence capture. Syst Biol. 2020;69(3): 479-501.

576. Oswald JN, Walmsley SF, Casey C, Fregosi S, Southall BL, Janik VMet al. Species information in whistle frequency modulation patterns of common dolphins. Phil Trans R Soc B. 2021;376: 20210046.

15. Richardson WJ, Thomson DH, Greene CR, Malme CI. Marine mammal sounds. In: Marine Mammals and Noise. San Diego, CA: Academic Press; 1995.

16. Oswald JN, Rankin S, Barlow J. The effect of recording and analysis bandwidth on acoustic identification of delphinid species. J Acoust Soc Am. 2004 Nov 1;116(5):3178–85.

17. Filadelfo R, Mintz J, Michlovich E, D'Amico A, Tyack PL, Ketten DR. Correlating military sonar use with beaked whale mass strandings: What do the historical data show? Aquat Mamm. 2009;35(4):435–44.

18. D'Amico A, Gisiner RC, Ketten DR, Hammock JA, Johnson C, Tyack PL, et al. Beaked whale strandings and naval exercises. Aquat Mamm. 2009;35(4):452–72.

Formatted: Indent: Left: 0", Hanging: 0.44", Don't add space between paragraphs of the same style

Formatted: Don't add space between paragraphs of the same style

Formatted: Indent: Left: 0", First line: 0", Don't add space between paragraphs of the same style

19. Guan S, Brookens T. An overview of research efforts to understand the effects of underwater sound on cetaceans. Vol. 2, Water Biology and Security. Institute of Hydrobiology, Chinese Academy of Sciences; 2023; 2:100141.

20. Ioannou CC, Laskowski KL. A multi-scale review of the dynamics of collective behaviour: from rapid responses to ontogeny and evolution. Philosophical Transactions of the Royal Society B. 202310;378(1874):20220059.Available from: https://doi.org/10.1098/rstb.2022.0059

21. Southall BL, DeRuiter SL, Friedlaender A, Stimpert AK, Goldbogen JA, Hazen E, et al. Behavioral responses of individual blue whales (*Balaenoptera musculus*) to mid-frequency military sonar. Journal of Experimental Biology. 2019;222(5).

22. Sivle LD, Kvadsheim PH, Fahlman A, Lam FPA, Tyack PL, Miller PJO, et al. Changes in dive behavior during naval sonar exposure in killer whales, long-finned pilot whales, and sperm whales. Frontiers in Physiology. 2012; 11;3:400. Available from: www.frontiersin.org

23. Deruiter SL, Boyd IL, Claridge DE, Clark CW, Gagnon C, Southall BL, et al. Delphinid whistle production and call matching during playback of simulated military sonar. Mar Mamm Sci. 2013;29(2).

24. Tyack PL, Zimmer WMX, Moretti D, Southall BL, Claridge DE, Durban JW, et al. Beaked whales respond to simulated and actual navy sonar. PLoS One. 2011;6(3).

25. Durbach IN, Harris CM, Martin C, Helble TA, Henderson EE, Ierley G, et al. Changes in the Movement and Calling Behavior of Minke Whales (*Balaenoptera acutorostrata*) in Response to Navy Training. Front Mar Sci. 2021;8.

26. Rendell LE, Gordon JCD. Vocal response of long-finned pilot whales (*Globicephala melas*) to military sonar in the Ligurian Sea. Mar Mamm Sci. 1999;15(1):198–204.

27. Miller PJO, Antunes RN, Wensveen PJ, Samarra FIP, Catarina Alves A, Tyack PL, et al. Dose-response relationships for the onset of avoidance of sonar by free-ranging killer whales. J Acoust Soc Am. 2014;135(2):975–93. Available from: http://asa.scitation.org/doi/10.1121/1.4861346

28. DeRuiter SL, Boyd IL, Claridge DE, Clark CW, Gagnon C, Southall BL, Tyack PL. Delphinid whistle production and call matching during playback of simulated military sonar. Marine Mammal Science. 2013 Apr;29(2):E46-59.Available from: https://onlinelibrary.wiley.com/doi/10.1111/j.1748-7692.2012.00587.x

30. Lara G, Bou-Cabo M, Llorens S, Miralles R, Espinosa V. Acoustical Behavior of Delphinid Whistles in the Presence of an Underwater Explosion Event in the Mediterranean Coastal Waters of Spain. Journal of Marine Science and Engineering. 2023;11(4):780. Available from: https://doi.org/10.3390/jmse11040780

31. Keen KA, Beltran RS, Pirotta E, Costa DP. Emerging themes in population consequences of disturbance models. Proceedings of the Royal Society B. 2021;288(1957):20210325.

32. King SL, Connor RC, Montgomery SH. Social and vocal complexity in bottlenose dolphins. Vol. 45, Trends in Neurosciences. Elsevier Ltd; 2022. p. 881–3.

33. Cunha HA, Loizaga De Castro R, Secchi ER, Crespo EA, Lailson-Brito J, Azevedo AF, et al. Molecular and morphological differentiation of common dolphins (*Delphinus* sp.) in the Southwestern Atlantic: testing the two species hypothesis in sympatry. PloS one. 2015 Nov 11;10(11):e0140251.

34. Durban JW, Southall BL, Calambokidis J, Casey C, Fearnbach H, Joyce TW, et al. Integrating remote sensing methods during controlled exposure experiments to quantify group

responses of dolphins to navy sonar. Mar Pollut Bull. 2022;174:113194. Available from: https://doi.org/10.1016/j.marpolbul.2021.113194

35. Southall BL, Moretti D, Abraham B, Calambokidis J, Deruiter SL, Tyack PL. Marine mammal behavioral response studies in southern California: advances in technology and experimental methods. Marine Technology Society Journal. 2012 Jul 1;46(4):48-59

36. Visser, F. (2014). Moving in concert: Social and migratory behaviour of dolphins and whalesin the North Atlantic Ocean. PhD dissertation. University of Amsterdam. 2014.

37. Hager CA. Assessment of the performance of the near-bottom hydrophones of the US Navy Southern California offshore range in detecting, localizing and reconstructing 10-20KHZ odontocete whistles. PhD dissertation. Naval Postgraduate School. 2008.

38. Oswald JN, Walmsley SF, Casey C, Fregosi S, Southall B, Janik VM. Species information in whistle frequency modulation patterns of common dolphins. Phil TransR Soc B. 2021;376:20210046.

39. Gillespie D, Mellinger DK, Gordon J, David McLaren, Redmond P, McHugh R, et al. PAMGUARD: Semiautomated, open source software for real- time acoustic detection and localization of cetaceans. J Acoust Soc Am. 2009;125(4):2547–2547.

40. Sakai T et al. R Package 'PAMpal'. https://doi.org/10.1121/1.2743157;2022.

41. Killick R, Fearnhead P, Eckley IA. Killick R, Fearnhead P, Eckley IA. Optimal detection of changepoints with a linear computational cost. Journal of the American Statistical Association. 2012 Dec 1;107(500):1590-8.

42. Brooks ME;, Kristensen K;, Van Benthem KJ;, Magnusson A;, Berg CW;, Nielsen A;, et al. glmmTMB balances speed and flexibility among packages for zero-inflated generalized linear

mixed modeling. The R journal. 2017;9(2):378-400. Available from: https://doi.org/10.3929/ethz-b-000240890

43. Goldbogen JA, Southall BL, DeRuiter SL, Calambokidis J, Friedlaender AS, Haz EL, et al. Blue whales respond to simulated mid-frequency military sonar. Proceeding of the Royal Society B. 2013;280:20130657.

44. Croll DA, Acevedo-Gutierrez A, Tershy BR, Urban-Ramırez J, Croll D. The diving behavior of blue and fin whales: is dive duration shorter than expected based on oxygen stores? Comparative Biochemistry and Physiology Part A: Molecular & Integrative Physiology. 2001 Jul 1;129(4):797-809.

45. Baird RW, Webster DL, Schorr GS, McSweeney DJ, Barlow J. Diel variation in beaked whale diving behavior. Mar Mamm Sci. 2008 Jul;24(3):630–42.

46. Shearer JM, Quick NJ, Cioffi WR, Baird RW, Webster DL, Foley HJ, et al. Diving behaviour of Cuvier's beaked whales (Ziphius cavirostris) off Cape Hatteras, North Carolina. R Soc Open Sci. 2019 Feb 1;6(2).

47. Deruiter SL, Southall BL, Calambokidis J, Zimmer WMX, Sadykova D, Falcone EA, et al. First direct measurements of behavioural responses by Cuvier ' s beaked whales to mid-frequency active sonar. 2013;9:20130223.

48. Egnor SR, Wickelgren JG, Hauser MD. Tracking silence: adjusting vocal production to avoid acoustic interference. Journal of Comparative Physiology A. 2007 Apr;193:477-83.

49. Kiai A, Clemens J, Kössl M, Poeppel D, Hechavarría JC. Flexible control of vocal timing in bats enables escape from acoustic interference. bioRxiv. 2023:2023-05.

Formatted: Don't add space between paragraphs of the same style

Formatted: Indent: Left: 0", First line: 0", Don't add space between paragraphs of the same style

50. Munoz-Santos I, Ríos-Chelén AA. Vermilion flycatchers avoid singing during sudden peaks of anthropogenic noise. acta ethologica. 2022 Dec 26:1-0.

51. Finneran JJ, Schlundt CE, Bowman V, Jenkins K. Dolphins reduce hearing sensitivity in anticipation of repetitive impulsive noise exposures. The Journal of the Acoustical Society of America. 2023 Jun 1;153(6):3372.

52. Bonabeau E, Theraulaz G, Deneubourg J.L, Aron S, Camazine S. Self-organization in social insects. Trends Ecol Evol. 1997;12(188).

53. Sumpter DJT. The principles of collective animal behaviour. Philosophical Transactions of the Royal Society B. 2005;361:5–22. Available from: https://royalsocietypublishing.org/

54. Caldwell MC, Caldwell DK. Vocalization of naive captive dolphins in small groups. Science. 1968 Mar 8;159(3819):1121-3.

55. Francis CD, Ortega CP, Cruz A. Noise Pollution Changes Avian Communities and Species Interactions. Current Biology. 2009 Aug 25;19(16):1415–9.

56. Brown CL, Hardy AR, Barber JR, Fristrup KM, Crooks KR, Angeloni LM. Brown CL, Hardy AR, Barber JR, Fristrup KM, Crooks KR, Angeloni LM. The effect of human activities and their associated noise on ungulate behavior. PloS one. 2012 Jul 10;7(7):e40505.

57. Southall BL, Tollit D, Amaral J, Clark CW, Ellison WT. Managing human activity and marine mammals: A biologically based, relativistic risk assessment framework. Front Mar Sci. 2023;10.

58. Harris CM, Thomas L, Falcone EA, Hildebrand J, Houser D, et al. Marine mammals and sonar: Dose- response studies, the risk- disturbance hypothesis and the role of exposure context.

J Appl Ecol. 2018;55:396-404. Available from: https://besjournals.onlinelibrary.wiley.com/doi/10.1111/1365-2664.12955

59. Harwood J, King S, Booth C, Donovan C, Schick RS, Thomas L, et al. Understanding the population consequences of acoustic disturbance for marine mammals. InThe effects of noise on aquatic life II 2016 (pp. 417-423). Springer New York.

60. McHuron EA, Adamczak S, Costa DP, Booth C. Estimating reproductive costs in marine mammal bioenergetic models: a review of current knowledge and data availability. Conservation Physiology. 2023;11(1):coac080.

61. Natoli A, Cañadas A, Peddemors VM, Aguilar A, Vaquero C, Fernandez- Piqueras P, Hoelzel AR. Phylogeography and alpha taxonomy of the common dolphin (*Delphinus* sp.). Journal of Evolutionary Biology. 2006 May;19(3):943-54.

62. Mcgowen MR, Tsagkogeorga G, Álvarez-Carretero S, Reis M Dos, Struebig M, Deaville R, et al. Phylogenomic resolution of the cetacean tree of life using target sequence capture. Systematic biology. 2020 May 1;69(3):479-501.

Supporting information

S1 Fig. PAMGuard Whistle Detection Parameters. Detection settings for the PAMGuard Whistle and Moan Detector.

S1 File. CEE-by-CEE Analysis. The main manuscript presents methods and results for analyzing common dolphin vocal response to simulated mid-frequency sonar, pooling data acrossfrom all CEEs. We acknowledge that some readers may be interested in additional

Formatted: Font: Bold

Formatted: Font: 18 pt, Bold

Formatted: Don't add space between paragraphs of the same style

Formatted: Don't add space between paragraphs of the same style, Line spacing: Double

Formatted: Don't add space between paragraphs of the same style

information about each of the 19 CEEs (10 MFAS exposures and nine controls), so we have included additional methods and results for each CEE.

S2 Fig. Plots of raw per-second whistle detections for all included CEEs. CEE-ID (year and number) is given in the bottom right of each page. The red dashed line indicates the start of the exposure period, with dashed grey lines indicating the timing of each ping (in an MFAS exposure) or control treatment. The solid gray vertical line indicates the start of the post-exposure period.

S3 Fig. Boxplots of the change in whistle count from the 5 s before to the 5 s following each of the 24 pings for all CEEs (MFAS and controls). Boxplot shows median, 25th, and 75th percentiles, with raw whistle count changes as open gray circles. The change following the first ping is shown as a red star.

Author contributions

BLS acquired the funding for this project, and BLS, CC, and SF conceived, designed, and performed the experiment. CC and SF analyzed the data, while BLS, VMJ, and JNO provided analytical guidance. CC, SF, VMJ, JNO, and BLS wrote the manuscript.

Formatted: Font: Bold

Formatted: Font: Bold

Formatted: Don't add space between paragraphs of the same style, Line spacing: Double

Formatted: Font: Bold

Formatted: Font: Bold

Formatted: Font: Not Bold

Formatted: Font: Bold

Formatted: Line spacing: Double

Formatted: Font: Bold

Formatted: Font: Bold

Formatted: Font: Bold

Formatted: Font: Bold

Formatted: Don't add space between paragraphs of the same style, Line spacing: Double

Formatted: Font: 18 pt, Bold

Formatted: Don't add space between paragraphs of the same style
