# Supplementary material for: Common dolphin whistle responses to experimental mid-frequency sonar
Source: PLoS One. 2024 Apr 26;19(4):e0302035. doi: 10.1371/journal.pone.0302035 (PMC11051594; doi:10.1371/journal.pone.0302035)
Supplement: Attachment — Submitted filename: PLOS_dolphin whistle response to Navy sonar review.docx [file pone.0302035.s007.docx]

Reviewer

Common dolphin whistle response to experimental mid-frequency sonar

*Caroline Casey, *Selene Fregosi, Julie N. Oswald, Vincent M. Janik, Fleur Visserd, Brandon Southall

Casey et al. aimed to measure the impact of Navy MFAS sonar on free-ranging dolphins in comparison to baseline variability data of acoustics. They utilize a network of acoustic buoys and controlled exposure experiments measure acoustic disturbance of free ranging short-beaked and long-beaked common dolphin whistles. CEEs were conducted off the coast of Santa Catalina Island and data were analyzed for vocal state changes. The authors found a significant vocal state change in the 5 seconds post CEEs. Interestingly, they give information on potential habituation of the animals as well.

Overall, it is a well written paper looking at vocal state changes as a tool for measuring disturbance. There are a few errors and areas that transition phrases are redundant. There are numerous areas where the authors have accidentally put in an extra space between words. I have highlighted most of what I found. I would suggest another pass over critically looking at punctuations and spacing.

Line 19: Considering we do not have data that states that millions hear and are affected by Navy MFAS in particular, I would suggest rephrasing this first sentence or adding a citation.

Line 222-223: While the author’s state that the distance between the drone and the octocopter were estimated every thirty minutes, it would be beneficial for the authors to state how high the drone was flying on average.

Line 427: Consider deleting “however” after Note.

Line 448: consider replacing en dash with an em dash.

Line 449: 10 MFAS CEEs were conducted but only 2 in Dolphinus delphis, why? I would suggest at least one line of explanation on this.

Line 483: cee is not capitalized in this line but is in front of the word type in the line before. I would suggest making this congruent in the paragraph.

Line 618: There is an extra space between the last word of the sentence and the period.

Line 647: There is an extra space between the words “sizes” and “where”.
