# Supplementary material for: Common dolphin whistle responses to experimental mid-frequency sonar
Source: PLoS One. 2024 Apr 26;19(4):e0302035. doi: 10.1371/journal.pone.0302035 (PMC11051594; doi:10.1371/journal.pone.0302035)
Supplement: Attachment — Submitted filename: Response to Reviewers_2.docx [file pone.0302035.s009.docx]

Response to Reviewers – 2

To the PLOS ONE editorial team,

Thank you for the additional feedback that Reviewer 2 provided most recently. We have addressed their concerns in the updated version of the manuscript, and these changes can be seen within the document “Revised Manuscript with Track Changes_2.” We hope that these edits satisfy PLOS ONE and the editorial team. Below, we highlight the specific changes that have been made to the manuscript to address Reviewer 2’s suggestions.

Line 21: **Reviewer’s comment:** “Do you mean “that occur in and around Navy operating areas?”

**Response:** Yes, we have changed the wording slightly here to make this clearer. It now reads, “Oceanic delphinids that occur in and around Navy operational areas…”

Line 50: **Reviewer’s comment:** Remove the word “activity.”

**Response:** This has been done.

Line 58: **Reviewer comment:** Change the order of wording in the sentence.

**Response:** This has been done; the sentence now reads, “Among cetaceans, oceanic delphinids represent an essential and logistically challenging group to evaluate how anthropogenic noise impacts their vocal behavior.”

Line 61: **Reviewer comment:** Delete extra space.

**Response**: This has been done.

Line 76: **Reviewer comment:** Replace the word “have” with “has.”

**Response:** This has been done.

Line 93: **Reviewer comment:** Change the order of words in this sentence.

**Response:** This has been done. The sentence now reads, “Previous studies on acoustic responses of oceanic dolphins to Navy sonar have observed shifts in frequency-specific components of whistle contours, increasing or decreasing calling rate, increasing call amplitude, and even mimicry of MFAS elements.”

Line 102: **Reviewer comment:** sp. Shouldn’t be italicized.

**Response:** This has been changed.

Line 167: **Reviewer comment:** 1 μPa/V should be revised to include a space throughout the ms.

**Response:** This has been addressed throughout the ms, including Table 1.

Line 243: **Reviewer comment:** change to 1.6-km threshold.

**Response:** This has been done.

Line 454: **Reviewer comment:** change the wording of the sentence.

**Response:** This has been done; the sentence now reads, “However, for the assessment of changes in whistle behavior across different time scales, CEE 2021_11 (conducted with long-beaked common dolphins) had to be excluded because the limited number of detected whistles could not be successfully modeled.”

Line 516: **Reviewer comment:** I believe, based on the caption format for PLOS ONE, this should be unbolded (similar to B), and a bolded general title of the figure should be added.

**Response:** This has been done, the figure caption now reads “**Fig 5. Acoustic response of common dolphins to the first ping of experimental sonar.** (A) Spectrogram example of 5 s before and 5 s after the first ping for MFAS CEE 2021_08 illustrates the significant increase in whistle count immediately following the cessation of the ping. The focal group was comprised of approximately 30 long-beaked common dolphins. The MFAS signal can be seen between 3 and 4 kHz. (B) Boxplot of the change in whistle count from the 5 s before to the 5 s following each of the 24 pings for CEE 2021_08. Boxplot shows median, 25th, and 75th percentiles, with raw whistle count changes as open gray circles. The change following the first ping is shown as a red star. “

Line 601: **Reviewer comment:** change to 5 s based on the format of the rest of the manuscript.

**Response:** This has been done.

Line 635: **Reviewer comment:** The date is missing, and the 57 should be placed after the date rather than at the end of the sentence.

**Response:** This has been changed. The sentence now reads “However, recent work by Oswald et al. 2021 [57] discovered unique whistle contours in short-beaked common dolphins, suggesting that these distinctive acoustic signals could help facilitate recognition between these *Delphinus* subspecies.”
